# GWAS of epigenetic aging rates in blood reveals a critical role for *TERT*

**DOI:** 10.1038/s41467-017-02697-5

**Published:** 2018-01-26

**Authors:** Ake T. Lu, Luting Xue, Elias L. Salfati, Brian H. Chen, Luigi Ferrucci, Daniel Levy, Roby Joehanes, Joanne M. Murabito, Douglas P. Kiel, Pei-Chien Tsai, Idil Yet, Jordana T. Bell, Massimo Mangino, Toshiko Tanaka, Allan F. McRae, Riccardo E. Marioni, Peter M. Visscher, Naomi R. Wray, Ian J. Deary, Morgan E. Levine, Austin Quach, Themistocles Assimes, Philip S. Tsao, Devin Absher, James D. Stewart, Yun Li, Alex P. Reiner, Lifang Hou, Andrea A. Baccarelli, Eric A. Whitsel, Abraham Aviv, Alexia Cardona, Felix R. Day, Nicholas J. Wareham, John R. B. Perry, Ken K. Ong, Kenneth Raj, Kathryn L. Lunetta, Steve Horvath

**Affiliations:** 1 Human Genetics, David Geffen School of Medicine, University of California, Los Angeles, Los Angeles, CA 90095 USA; 2 Department of Biostatistics, Boston University School of Public Health, Boston, MA 02118 USA; 3 Department of Medicine, Stanford University School of Medicine, Stanford, CA 94305 USA; 4 Intramural Research Program, National Institute on Aging, National Institutes of Health, Baltimore, MD 21224 USA; 5 National Heart, Lung and Blood Institute, Bethesda, MD 20824-0105 USA; 6 Department of Medicine, Section of General Medicine, Boston University School of Medicine, Boston, MA 02118 USA; 7 Institute for Aging Research, Hebrew SeniorLife, Beth Israel Deaconess Medical Centre, Harvard Medical School, Boston, MA 02215 USA; 8 Department of Twin Research and Genetic Epidemiology, Kings College London, London, SE1 7EH UK; 9 Institute for Molecular Bioscience, The University of Queensland, Brisbane, 4072 QLD Australia; 10 Queensland Brain Institute, The University of Queensland, Brisbane, 4072 QLD Australia; 11 Centre for Cognitive Aging and Cognitive Epidemiology, Department of Psychology, University of Edinburgh, 7 George Square, Edinburgh, EH8 9JZ UK; 12 Medical Genetics Section, Centre for Genomic and Experimental Medicine, Institute of Genetics and Molecular Medicine, University of Edinburgh, Edinburgh, EH4 2XU UK; 13 VA Palo Alto Health Care System, Palo Alto, CA 94304 USA; 14 HudsonAlpha Institute for Biotechnology, Huntsville, AL 35806 USA; 15 Department of Epidemiology, Gillings School of Global Public Health, University of North Carolina, Chapel Hill, NC 27599 USA; 16 Department of Genetics, School of Medicine, University of North Carolina, Chapel Hill, NC 27599 USA; 17 Department of Biostatistics, Gillings School of Global Public Health, University of North Carolina, Chapel Hill, NC 27599 USA; 18 Fred Hutchinson Cancer Research Center Box 358080, WHI Clinical Coordinating Ctr/Public Health Sciences M3-A4, Seattle, WA 98109 USA; 19 Department of Preventive Medicine, Feinberg School of Medicine, Northwestern University Chicago, Evanston, IL 60611 USA; 20 Center for Population Epigenetics, Robert H. Lurie Comprehensive Cancer Center, Feinberg School of Medicine, Northwestern University Chicago, Evanston, IL 60611 USA; 21 Laboratory of Environmental Epigenetics, Departments of Environmental Health Sciences Epidemiology, Columbia University Mailman School of Public Health, New York, NY 10032 USA; 22 Department of Medicine, School of Medicine, University of North Carolina, Chapel Hill, NC 27516 USA; 23 The Center for Human Development and Aging, University of Medicine and Dentistry, New Jersey Medical School, Rutgers, Newark, NJ 07103 USA; 24 Medical Research Council (MRC) Epidemiology Unit, University of Cambridge School of Clinical Medicine, Institute of Metabolic Science, Cambridge Biomedical Campus, Cambridge, CB2 0SL UK; 25 Department of Paediatrics, University of Cambridge School of Clinical Medicine, Cambridge Biomedical Campus, Cambridge, CB2 0SP UK; 26 Radiation Effects Department, Centre for Radiation, Chemical and Environmental Hazards, Public Health England, Chilton, Didcot, Oxfordshire OX11 0RQ UK; 27 Biostatistics, School of Public Health, University of California, Los Angeles, Los Angeles, CA 90095 USA

## Abstract

DNA methylation age is an accurate biomarker of chronological age and predicts lifespan, but its underlying molecular mechanisms are unknown. In this genome-wide association study of 9907 individuals, we find gene variants mapping to five loci associated with intrinsic epigenetic age acceleration (IEAA) and gene variants in three loci associated with extrinsic epigenetic age acceleration (EEAA). Mendelian randomization analysis suggests causal influences of menarche and menopause on IEAA and lipoproteins on IEAA and EEAA. Variants associated with longer leukocyte telomere length (LTL) in the telomerase reverse transcriptase gene (*TERT*) paradoxically confer higher IEAA (*P* < 2.7 × 10^−11^). Causal modeling indicates *TERT*-specific and independent effects on LTL and IEAA. Experimental hTERT-expression in primary human fibroblasts engenders a linear increase in DNA methylation age with cell population doubling number. Together, these findings indicate a critical role for hTERT in regulating the epigenetic clock, in addition to its established role of compensating for cell replication-dependent telomere shortening.

## Abstract (web summary)

Epigenetic clocks based on DNA methylation levels are estimators of chronological age. Here, the authors perform a GWAS of epigenetic aging rates in blood and find SNP variants in the *TERT* locus associated with increased intrinsic epigenetic age are also associated with longer telomeres.

## Introduction

DNA methylation (DNAm) profiles of sets of cytosine phosphate guanines (CpGs) allow one to develop accurate estimators of chronological age which are referred to as “DNAm age”, “epigenetic age”, or the “epigenetic clock”. Across the life course the correlation between DNAm age and chronological age is >0.95^1,2^. Individuals whose leukocyte DNAm age is older than their chronological age (“epigenetic age acceleration”) display a higher risk of all-cause mortality after accounting for known risk factors^3–6^, and offspring of centenarians exhibit a younger DNAm age^7^. Epigenetic age acceleration in blood is associated with cognitive impairment, neuro-pathology in the elderly^8,9^, Down syndrome^10^, Werner syndrome^11^, Parkinson’s disease^12^, obesity^13^, HIV infection^14^, and frailty^15^, menopause^16^ but it only displays weak correlations with clinical biomarkers^17,18^. DNAm age shows no apparent correlation with leukocyte telomere length (LTL), whose pace of shortening in cultured somatic cells has been referred to as the ‘mitotic clock’. In vivo, DNAm age and telomere length appear to be independent predictors of mortality^19^.

Here we examine two widely used measures of epigenetic age acceleration: (a) intrinsic epigenetic age acceleration (IEAA), based on 353 CpGs described by Horvath^2^, which is independent of age-related changes in blood cell composition, and (b) extrinsic epigenetic age acceleration (EEAA), an enhanced version of that based on 71 CpGs described by Hannum (2013) which up-weights the contribution of blood cell count measures^1,6^. IEAA and EEAA are only moderately correlated (*r* = 0.37). IEAA measures cell-intrinsic methylation changes, exhibits greater consistency across different tissues, appears unrelated to lifestyle factors and probably indicates a fundamental cell aging process that is largely conserved across cell types^2,6^. By contrast, EEAA captures age-related changes in leukocyte composition and correlates with lifestyle and health-span related characteristics, yielding a stronger predictor of all-cause mortality^6,18^. To dissect the genetic architecture underlying DNAm age of blood, we performed genome-wide association studies (GWAS) of IEAA and EEAA based on leukocyte DNA samples from almost 10,000 individuals. Our GWAS identifies a total of five loci associated with IEAA and three loci associated with EEAA at genome-wide significance. One of the loci associated with IEAA co-locates with the Telomerase Reverse Transcriptase (*TERT*) gene on chromosome 5. Variants in *TERT* that are associated with increased IEAA are also associated with longer telomeres. Our in vitro experiments indicate that hTERT expression is required for DNAm aging in human primary fibroblast cells. Finally, our Mendelian randomization analyses reveal that age at menarche and age at menopause have a causal effect on IEAA.

## Results

### GWAS meta-analyses for IEAA and EEAA

**Fig. 1:**
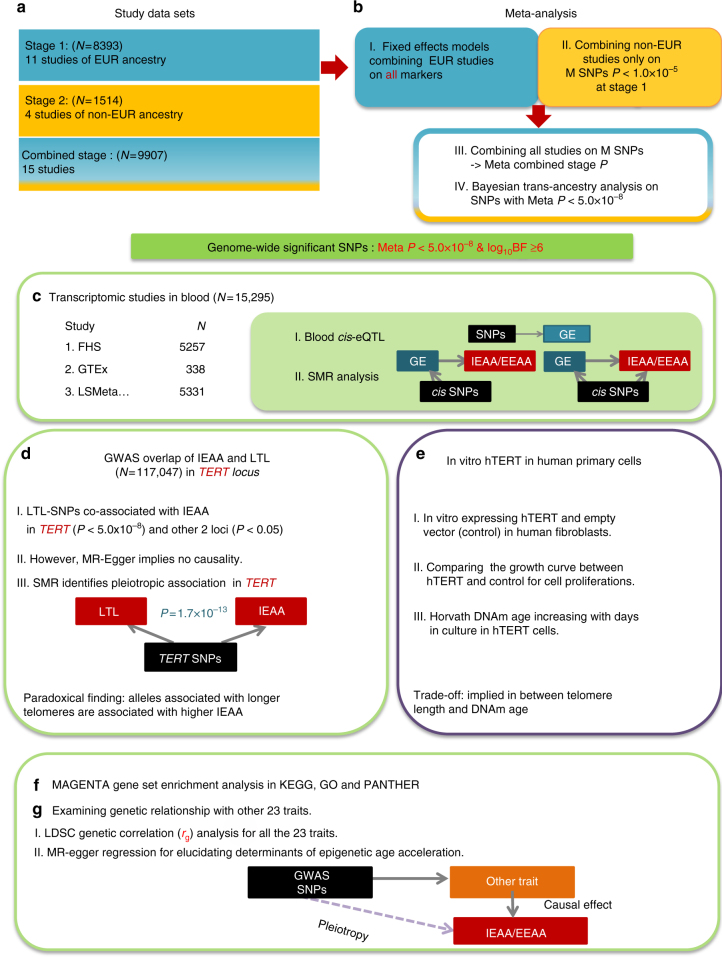
Roadmap for studying genetic variants associated with epigenetic age acceleration in blood. The roadmap depicts our analytical procedures. **a** The study sets were divided into two stages according to European (EUR) and non-European ancestry. **b** Stage 1 yielded GWAS summary data on all QC SNPs and the combined stage yielded GWAS summary data on the SNPs with Meta EUR *P* < 1.0 × 10^−5^ at stage 1. Genome-wide significant loci were determined based on the association results from the combined stage. **c** Describes our transcriptomic studies: (I) blood *cis*-eQTL to identify potential functional genes, (II) summary statistics based Mendelian randomization (SMR) to assess the causal associations between expression levels and IEAA (or EEAA). **d** Describes our detailed analysis in the TERT locus, which was implicated by our GWAS of IEAA. Bidirectional Mendelian randomization via MR-Egger analysis did not reveal a direct causal effect between leukocyte telomere length and IEAA. Our in vitro studies validate our genetic findings by demonstrating that hTERT over-expression promotes epigenetic aging in **e**. To explore molecular pathways underlying epigenetic age acceleration, we conducted gene set enrichment analysis, as listed in **f**. Finally, we performed LDSC genetic correlation between IEAA or EEAA and a broad category of complex traits, followed by MR-Egger regression analysis, as depicted in **g**. Abbreviations: GE = gene expression, LTL = leukocyte telomere length

Genomic analyses were performed in 9907 individuals (aged 10–98 years), from 15 data sets, adjusted for chronological age and sex (Supplementary Table 1, Fig. 1, and Supplementary Note 1). Eleven data sets comprised individuals of European ancestry (84.7%) and four comprised individuals of African (10.3%) or Hispanic ancestry (5.0%). GWAS genotypes were imputed to ~7.4 million variants using the 1000 genomes reference panel. To estimate the heritability of epigenetic age acceleration in blood, we used both pedigree- and SNP-based methods (Methods section). Our pedigree-based estimates of heritability were $h_{{\rm IEAA}}^2$ = 0.37 and $h_{{\rm EEAA}}^2$ = 0.33 in individuals of European ancestry, which is consistent with previous heritability estimates in twins^2^ and with those obtained in other tissues (e.g., adipose and brain)^9,13,20^. SNP-based estimates of heritability in our European ancestry cohorts were lower, $h_{{\rm IEAA}}^2$ = 0.19 and $h_{{\rm EEAA}}^2$ = 0.19 (Supplementary Table 2).

We first performed GWAS meta-analysis of IEAA and EEAA only in our European ancestry cohorts (*N* = 8393). Variants with suggestive associations (*P* < 1.0 × 10^−5^) were then evaluated in non-European ancestry cohorts (*N* = 1514), followed by a combined meta-analysis across the two strata (Fig. 1a, b). We found no evidence for genomic inflation in individual studies ($\lambda _{{\rm GC}} = 0.99$ ~ 1.06, Supplementary Tables 3 and 4) or in the European ancestry meta-analysis (*λ*_GC_ = 1.03 ~ 1.05; LD score regression (LDSC) intercept terms $\beta _{0,{\rm IEAA}} = 1.004$ and $\beta _{0,{\rm EEAA}} = 1.004$; Supplementary Fig. 1 and Supplementary Table 2). Variant associations that were genome-wide significant (*P* < 5.0 × 10^−8^) according to the fixed-effect meta-analysis were also evaluated with the trans-ethnic heterogeneity test that is implemented in the MANTRA software^21^. The MANTRA software provides a meta-analysis estimate, known as Bayes Factor (BF), that is arguably more stringent than the fixed-effects meta-analysis because it accounts for genetic heterogeneity between different study populations. Our study focused on genetic variants that met two criteria: fixed effects meta *P* < 5.0 × 10^−8^ and MANTRA ${\rm log}_{10}{\rm BF} \ge 6$ (≅*P* < 5 × 10^−8^).

For IEAA, we identified 264 associated variants, mapping to five genomic loci (3q25.33, 5p15.33, 6p22.3, 6p22.2, and 17q22, Table 1, Supplementary Data 1, Fig. 2, and Supplementary Fig. 2). Conditional analyses revealed a secondary signal for IEAA at 6p22.3 (Table 1, Supplementary Figs. 3c and h). For EEAA, we identified 440 associated variants, mapping to three loci (4p16.3, 10p11.1, and 10p11.21; Table 1, Supplementary Data 1, Fig. 2, and Supplementary Fig. 4); however, the two lead SNPs, rs71007656 and rs1005277 at 10p11.1 and 10p11.21, respectively, are moderately correlated ($r_{{\rm EUR}}^2$ = 0.35, Table 1). Conditional analysis showed that the association of the INDEL variant rs71007656 partially derived from its association with rs1005277 because the *P*-value of rs71007656 was no longer genome-wide significant in a conditional model of rs1005277. By contrast, the SNP rs1005277 remained genome-wide significant in a conditional model of rs71007656 (Supplementary Fig. 5b, c and e, f). Associations were consistent across studies (Supplementary Figs. 6 and 7), except for one locus (6p22.3: Cochran’s *I*^2 ^= 58%, MANTRA posterior probability of heterogeneity = 0.64, Table 1 and Supplementary Data 1). At each of the five IEAA related loci, the risk alleles conferred between 0.41 and 1.68 years higher IEAA (Table 1). Of the five loci associated with IEAA, four also exhibited at least suggestive and sign-consistent associations with EEAA (most significant *P* = 6.6 × 10^−7^, Supplementary Data 2. By contrast, SNPs in the three loci associated with EEAA were not associated (*P* > 0.05) with IEAA (Supplementary Data 2). Analysis of published chromatin state marks^22^ showed that most lead variants are in chromosomal regions that are transcribed in multiple cell lines (Supplementary Fig. 8). Two loci, 6p22.2 and 6p22.3, co-locate (within 1 Mb) with CpGs that contribute to the Horvath estimate of DNAm age (Table 1 and Supplementary Data 1), and it is possible that these genotypic associations with IEAA arise from direct SNP effects on local methylation (Supplementary Note 2 and Supplementary Figs. 9 and 10).

**Table 1:** Meta-analysis of GWAS of epigenetic age acceleration in blood

							Fixed-effects			Trans-ethnic		
Band	*N*_GWAS_	SNP	Gene	Mb	A1/A2	MAF	Stage	Beta (SE)	*P*-value	*I* ^2^	${{\rm log}}_{10}{{\rm BF}}$	*P*_HET._
*IEAA*												
3q25.33	23	rs11706810	*TRIM59*	160.2	C/T	0.45	EUR	0.40 (0.07)	2.8 × 10^−8^			
							Non-EUR	0.44 (0.19)	1.8 × 10^−2^			
							Combined	0.41 (0.07)	1.6 × 10^−9^	3%	7.5	0.26
5p15.33	11	rs2736099	*TERT*	1.3	A/G	0.36	EUR	0.64 (0.09)	4.7 × 10^−12^			
							Non-EUR	0.50 (0.30)	9.9 × 10^−2^			
							Combined	0.63 (0.09)	1.3 × 10^−12^	0%	10.6	0.47
6p22.3	104	rs143093668^a^	*KIF13A-NHLRC1*	18.1	T/C	0.05	EUR	−1.78 (0.10)	1.9 × 10^−21^			
							Non-EUR	−1.37 (0.33)	2.4 × 10^−5^			
							Combined	−1.68 (0.16)	4.2 × 10^−25^	58%	23.1	0.64
		rs6915893^b,c^	*KIF13A-NHLRC1*	18.1	T/C	0.39	EUR	0.56 (0.08)	5.1 × 10^−13^			
							Non-EUR	0.33 (0.17)	5.8 × 10^−2^			
							Combined	0.52 (0.07)	1.6 × 10^−13^	27%	11.4	0.34
6p22.2	108	rs73397619^d^	*LRRC16A*-*SCGN*	25.6	C/T	0.29	EUR	−0.46 (0.08)	5.9 × 10^−9^			
							Non-EUR	−0.46 (0.18)	1.2 × 10^−2^			
							Combined	−0.46 (0.03)	2.3 × 10^−10^	18%	8.3	0.32
17q22	18	rs78781855	*STXBP4*	53.1	G/T	0.22	EUR	−0.42 (0.09)	1.6 × 10^−6^			
							Non-EUR	−0.88 (0.23)	1.5 × 10^−4^			
							Combined	−0.47 (0.08)	5.6 × 10^−9^	26%	7.2	0.45
*EEAA*												
4p16.3	59	rs10937913	*TNIP2*	2.8	A/G	0.46	EUR	−0.60 (0.01)	3.8 × 10^−10^			
							Non-EUR	−0.56 (0.23)	1.4 × 10^−2^			
							Combined	−0.59 (0.09)	1.7 × 10^−11^	0%	9.2	0.24
10p11.21	59	rs71007656	*ANKRD30A-ZNF248*	40.0	R/I^e^	0.49	EUR	0.61 (0.1−)	1.1 × 10^−9^			
							Non-EUR	0.52 (0.22)	2.0 × 10^−2^			
							Combined	0.59 (0.09)	7.5 × 10^−11^	0%	8.5	0.29
10p11.1	322	rs1005277	*ZNF248-ZNF25*	38.2	A/C	0.28	EUR	0.78 (0.11)	2.6 × 10^−13^			
							Non-EUR	0.45 (0.29)	1.1 × 10^−1^			
							Combined	0.74 (0.10)	1.2 × 10^−13^	32%	11.5	0.32

Position Mb based on Hg19 assembly

Lead SNPs at genome-wide significant (*P*  < 5.0 × 10^−8^) loci for IEAA or EEAA. Epigenetic Clock CpGs that co-locate within  ±1 Mb of the leading variant are listed in the footnote. Fixed effects meta-analysis was used to estimate the effect size (Beta) and standard error (SE) on IEAA or EEAA per minor allele. Trans-ethnic analyses using MANTRA^21^ present ethnicity-adjusted associations (log_10_ Bayes’ Factor (BF) and probability of heterogeneity across studies (*P*_HET._)

*N*_GWAS_ = number of GWAS markers, A1/A2 = minor/major alleles, MAF = mean of minor allele frequency estimates across studies weighted by study sample sizes, *I*^2^ = Cochran’s *I*^2^. Beta estimate is the regression coefficient with respect to each extra minor allele

^a^ The CpG predictor cg22736354 for epigenetic clock is located 8.5 kb from the leading variant

^b^ The CpG predictor cg22736354 for epigenetic clock is located 12.2 kb from the leading variant

^c^ Conditional analysis on rs143093668 (LD EUR *r*^2^ = 0.02): Beta(SE) = 0.39 (0.069) with effect size dropped 26% and conditional Meta *P*-value at combined phase = 2.6 × 10^−8^

^d^ The CpG predictor cg06493994 for epigenetic clock is located 27.8 kb from the leading variant

^e^ Reference/insertion alleles = C/CGGCTG

**Fig. 2:**
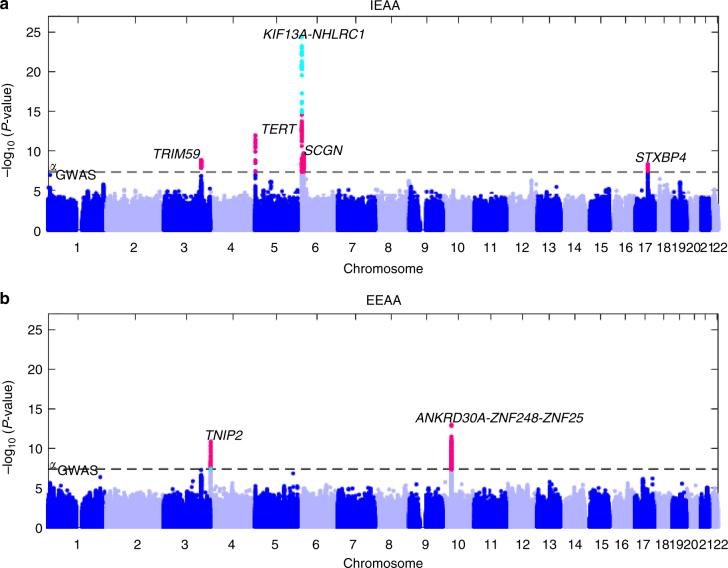
Genome-wide meta-analysis for intrinsic and extrinsic age acceleration in blood. Manhattan plots for the meta-analysis *P*-values resulting from 15 studies comprised of 9907 individuals. The *y*-axis reports log transformed *P*-values for **a** intrinsic epigenetic age acceleration (IEAA) or **b** extrinsic epigenetic age acceleration (EEAA). The horizontal dashed line corresponds to the threshold of genome-wide significance (*P* = 5.0 × 10^−8^). Genome-wide significant common SNPs (MAF ≥ 5%) and low frequency SNPs (2% ≤ MAF < 5%) are colored red and cyan, respectively

### Transcriptomic studies in leukocytes

To learn about potential functional consequences of these associations, we conducted *cis*-eQTL analysis for each locus associated with IEAA or EEAA, using data on leukocyte mRNA expression in up to 15,295 samples from five studies (Fig. 1c; Methods section). We identified 11 putative *cis*-eQTLs located in seven of the eight associated loci (Supplementary Data 3). Each putative *cis*-eQTL was then analyzed by summary data-based Mendelian randomization (SMR), which infers the association between gene transcript levels and the outcome trait^23^ (Methods section). Three transcripts were associated with IEAA: *KPNA4* at 3q25.33, *TPMT* at 6p22.3 and *STXBP4* at 17q22; and three transcripts were associated with EEAA: *RNF4* at 4p16.3, *ZNF25* at 10p.21, and *HSD17B7P2* at 10p11.21 and 10p11.1 (Table 2 and Supplementary Table 5). Notably, *STXBP4*, encoding the syntaxin-binding protein, is a reported locus for age at menarche^24^, and our lead SNP for IEAA was also associated with age at menarche (rs78781855, *P* = 0.0002).

**Table 2:** Summary of transcriptomic studies for loci associated with epigenetic age acceleration

		Meta GWAS	*cis*-eQTL			SMR
Band	SNP	*P*-value (sign)	Database	Gene	*P*-value (sign)	*P*-value (sign)
*IEAA*						
3q25.33	rs11706810	1.6 × 10^–9^ (+)	FHS	*KPNA4*	4.2 × 10^–23^ (+)	5.1 × 10^−5^ (+)
6p22.3	rs6915893	1.6 × 10^–13^ (+)	FHS	*TPMT*	6.0 × 10^–14^ (+)	1.1 × 10^−7^ (+)
			GTEx		1.5 × 10^–4^* (+)	2.2 × 10^−6^ (+)
			LSMeta		2.3 × 10^–27^ (+)	3.5 × 10^−7^ (+)
17q22	rs78781855	5.6 × 10^–9^ (–)	FHS	*STXBP4*	1.0 × 10^–88^ (–)	6.2 × 10^−4^ (+)
*EEAA*						
4p16.3	rs2341303^a^	6.5 × 10^–11^ (–)	LSMeta	*RNF4*	1.6 × 10^–10^ (–)	2.0 × 10^−3^ (+)
10p11.21	rs71007656	7.5 × 10^–11^ (+)	FHS	*ZNF25*	7.9 × 10^–7^ (+)	2.8 × 10^−3^ (+)
			GTEx	*HSD17B7P2*	3.0 × 10^–8^ (+)	6.9 × 10^−6^ (+)^b^
10p11.1	rs1005277	1.2 × 10^–13^ (+)	GTEx	*HSD17B7P2*	1.1 × 10^–5^ (+)	6.9 × 10^−6^ (+)^b^

Bands corresponding the position of Meta GWAS SNP

The table presents a total of six *cis* genes highlighted from transcriptomic study using three large-scale databases (*N* = 10,906), including (1) FHS (*N* = 5257), (2) GTEx (*N* = 338), and (3) LSMeta (5311). Each *cis* gene exhibited significant *cis*-eQTL with several nearby GWAS SNPs at FDR *q* < 0.05 in at least one study and also showed a significant pleiotropic association in SMR analysis at *P* < 0.05 after Bonferroni correction. For each gene, we list the unadjusted *P*-values (sign) from Meta GWAS for IEAA (or EEAA), *cis*-eQTL and SMR analysis. The column (sign) indicates the sign of *Z*-statistic at each test while the test alleles were converted to the same alleles (with minor variants) for both Meta GWAS and *cis*-eQTL tests. The summary statistics of Meta GWAS and *cis*-eQTL are both based on the leading marker with the most significant *P*-value in a given locus, according to the GWAS results

* FDR > 0.05 but FDR < 0.05 associated with other GWAS SNPs (Supplementary Data 3)

^a^ The SNP rs2341303 is a surrogate of the leading marker rs10937913 in 4p16.3 (LD $r_{{\rm EUR}}^2$ = 0.98)

^b^ The SMR results are derived from the same model as the analysis used the *cis* SNPs of the gene *HSD17B7P2*

### Paradoxical SNP association between IEAA and LTL in *TERT*

**Fig. 3:**
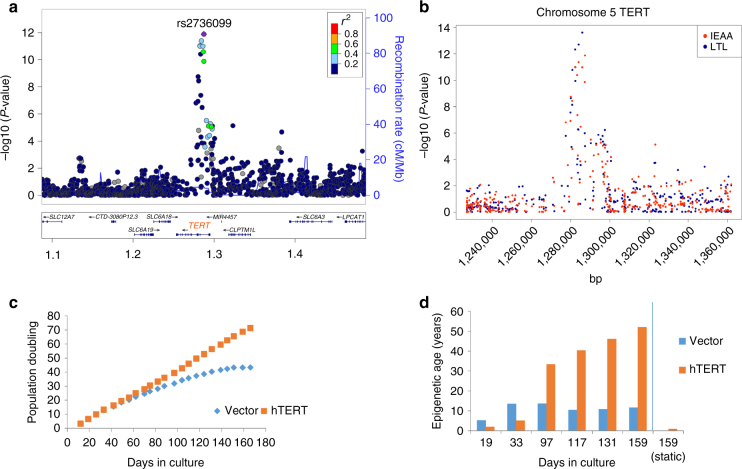
Genetic analysis of the 5p15.33 *TERT* locus and in vitro studies of hTERT in fibroblasts. **a** Regional association plot of locus associated with IEAA. The *y*-axis depicts log-transformed meta-analysis *P-*values across all studies 1–15. The colors visualize linkage disequilibrium (LD) *r*^2^ between rs2736099 (colored in purple) and neighboring SNPs. **b** *TERT*-locus association with IEAA (marked in red) overlaid with the association with leukocyte telomere length (LTL) given by Bojesen et al.^25^ (marked in blue). Note that several SNPs in the *TERT* locus are associated with both IEAA and leukocyte telomere length at a genome-wide significant level. **c** Growth of human primary fibroblasts (*n* = 46) represented as population doublings (*y*-axis) vs. days in culture (*x*-axis). **d** Adjusted epigenetic age of *n* = 14 individual samples (*y*-axis) vs. days in culture (*x*-axis). The adjusted age estimate was defined as difference between Horvath DNAm age minus 28 years, since the former exhibited a substantial offset in fibroblasts. DNAm ages are increased in the hTERT expressing cells in the four later time points (days 97, 117, 131, and 159) resulting in a paired Student's *t*-test *P*-value of 0.043. However, no increase in DNAm age can be observed in cells that are static (last bar)

The *TERT* locus (in 5p15.33) harbored 11 genome-wide significant SNPs for IEAA but conditional analysis did not reveal any secondary signal (Table 1, Fig. 3a, and Supplementary Data 1). The leading SNP, rs2736099, was located in a region transcribed in human embryonic stem cells, induced pluripotent stem cells, and hematopoietic stem cells (Supplementary Fig. 8b), and each minor allele conferred 0.6 years higher IEAA (*P* = 1.3 × 10^−12^; Table 1, Supplementary Fig. 6b). Our IEAA locus at *TERT* closely overlaps the reported GWAS locus for LTL^25–27^ (Fig. 3b). To further demonstrate that our GWAS findings surrounding *TERT* and IEAA are not mediated by LTL, we carried out an additional GWAS analysis of an LTL adjusted measure of IEAA in the subset (*n* = 785) of individuals for whom both IEAA and LTL were available. As expected, the LTL adjusted measure of IEAA continued to exhibit significant associations with the variants in the *TERT* locus (Methods section and Supplementary Fig. 11).

SMR analysis indicated that the association signals for LTL and IEAA at this *TERT* locus share the same underlying causal variant (as indicated by a non-significant HEIDI test, Supplementary Table 6, Supplementary Fig. 12c). Intriguingly, *TERT* alleles associated with a longer LTL were robustly associated with increased IEAA (*P* ~ 1.0 × 10^−11^, Table 3, Supplementary Table 7, and Supplementary Fig. 13).

**Table 3:** Several leukocyte telomere length associated SNPs are also associated with intrinsic epigenetic age acceleration in blood

Study	Chr	Gene	SNP	A1	MAF	Effect size (*β*)			Meta *P*-value		
						LTL	IEAA	EEAA	LTL	IEAA	EEAA
II	2	*ACYP2*	rs11125529	A	0.14	0.06	−0.06	−0.15	4.5 × 10^−8^	0.5	2.5 × 10^−1^
III	3	*PXK*	rs6772228	A	0.06	−120	0.26	0.20	3.9 × 10^−10^	1.3 × 10^−1^	3.7 × 10^−1^
II	3	*TERC*	rs10936599	T	0.24	−0.10	0.04	−0.07	2.5 × 10^−31^	0.7	4.7 × 10^−1^
III	3		rs1317082	G	0.23	−77	0.04	−0.08	1.3 × 10^−19^	0.7	4.6 × 10^−1^
II	4	*NAF1*	rs7675998	A	0.22	−0.07	−0.07	0.04	4.4 × 10^−16^	4.4 × 10^−1^	0.7
III	5	*TERT*	rs7726159	A	0.33	73	0.67	0.27	4.7 × 10^−17^	**9.5** × **10**^−**12**^	6.4 × 10^−2^
I	5		rs7705526	A	0.33	0.51	0.61	0.18	2.3 × 10^−14^	**1.0** × **10**^−**11**^	1.9 × 10^−1^
II	5		rs2736100	C	0.50	0.08	0.49	0.19	4.4 × 10^−19^	**2.7** × **10**^−**11**^	8.2 × 10^−2^
III	10	*OBFC1*	rs2487999	T	0.14	100	0.22	0.36	4.2 × 10^−14^	3.7 × 10^–2^	8.8 × 10^–3^
II	10		rs9420907	C	0.20	0.07	0.26	0.20	6.9 × 10^−11^	4.1 × 10^–3^	9.9 × 10^−2^
II	16	*MPHOSPH6*	rs2967374	A	0.22	0.05	0.23	0.12	2.7 × 10^−7^	7.8 × 10^–3^	2.9 × 10^−1^
II	19	*ZNF208*	rs8105767	G	0.32	0.05	−0.05	−0.13	1.1 × 10^−9^	0.5	1.7 × 10^−1^
III	20	*BCL2L1*	rs6060627	T	0.34	36	−0.13	0.11	6.5 × 10^−7^	7.7 × 10^−2^	2.6 × 10^−1^
II	20	*RTEL1*	rs755017	G	0.15	0.06	0.03	−0.08	6.7 × 10^−9^	0.8	0.5

*P*-values associated with age acceleration marked in italic if <0.05 and bold if <5.0 × 10^−8^

The table relates genome-wide significant association results of leukocyte telomere length (LTL) to two epigenetic age acceleration measures, IEAA and EEAA. We queried the results of 14 SNPs across 10 distinct susceptibility loci associated with LTL from three large-scale studies: (I) meta-analysis association of LTL in chromosome 5 *TERT* only (*N* = 53,724)^25^, (II) a genome-wide meta-analysis of LTL (*N* = 37,684)^26^, and (III) a genome-wide meta-analysis of LTL (*N* = 26,089)^27^. Each row presents a genome-wide significant locus associated with LTL in a given study, except chromosome 16 *MPHOSPH6* and chromosome 20 *BCL2L1* just slightly below genome-wide significance and highlighted by the corresponding studies as major findings. The listed markers are the leading SNPs with the most significant *P*-values associated with LTL at a given study and locus, sorted by chromosome, and position. Effect sizes of LTL association refer to the change in telomere lengths (ΔTL). Telomere lengths were measured based on the relative telomere to single copy gene (T/S) ratios using standard qualitative PCR methods. A wide range of effect sizes for ΔTL across studies was due to different scaling approaches applied to the measurements. The effect sizes for each age acceleration measure are in units of year

Chr = chromosome, A1 = reference allele, MAF = minor allele frequency; effect sizes corresponding additive models

Other known GWAS LTL signals (at 10q24.33 near *OBFC1* and at 16q23.3 near *MPHOSPH6*), also exhibited modest associations with IEAA (4.1 × 10^−3^ ≤ *P* ≤ 3.7 × 10^−2^), but others, such as the gene *TERC* (on 3q26.2) encoding the telomerase RNA component, showed no association (Table 3). Using the lead variants for each trait in MR-Egger analyses^28^, we found pleiotropic effects specific to *TERT* on LTL and IEAA without evidence for a causal relationship between LTL and IEAA (*P* = 0.7), Table 4 and Supplementary Table 8.

**Table 4:** Genetic correlations with and causal effects of other complex traits on epigenetic age acceleration

		IEAA				EEAA			
		Genetic correlation		MR-Egger regression		Genetic correlation		MR-Egger regression	
Trait	*N*	*r*_g_	${P}_{{r}_{{\rm g}}}$	${\beta }_{{\rm {causal}}}$	${P}_{{\rm {causal}}}$	*r*_g_	${P}_{{r}_{\rm {g}}}$	${\beta }_{{\rm {causal}}}$	${P}_{{\rm {causal}}}$
*Category (I)*									
Waist circumference (cm)	232,101	0.10	**2.2** × **10**^−**2**^	1.99	9.6 × 10^−2^	0.15	**9.0** × **10**^−**4**^	−1.48	0.3
Waist-to-hip ratio	212,243	0.06	0.20	−0.31	0.9	0.14	**2.7** × **10**^−**3**^	−3.36	0.3
BMI (SD)	339,224	−0.01	0.9	1.40	5.6 × 10^−2^	0.08	0.3	0.40	0.7
Height (cm)	133,453	−0.02	0.7	0.004	0.1	0.13	**2.8** × **10**^−**3**^	0.004	0.2
*Category (II)*									
HDL (SD)	188,577	−0.08	0.1	0.37	0.1	−0.12	**2.0** × **10**^−**2**^	0.11	0.7
Triglyceride (SD)	188,577	0.10	**3.4** × **10**^−**2**^	0.61	**3.0** × **10**^−**2**^	0.16	**1.0** × **10**^−**4**^	0.37	8.0 × 10^−2^
Type 2 diabetes	69,033	0.16	**3.5** × **10**^−**2**^	0.08	0.8	0.09	0.2	0.07	0.8
IBD	34,652	0.12	**2.5** × **10**^−**2**^	0.13	0.5	0.08	0.1	−0.35	0.2
Crohn’s disease	20,883	0.12	**4.7** × **10**^−**2**^	0.10	0.4	0.10	6.9 × 10^−2^	−0.29	7.6 × 10^−2^
*Category (III and IV)*									
AMD subtype	45,818	0.03	0.2	−0.05	0.6	0.05	**2.1** × **10**^−**2**^	−0.04	0.7
Edu. attainment (years)	328,917	−0.01	0.8	2.35	0.3	−0.13	**1.0** × **10**^−**3**^	−4.63	7.8 × 10^−2^
*Category (V)*									
Age at menarche (years)	252,514	−0.02	0.8	−1.03	**4.1** × **10**^−**3**^	−0.03	0.7	−0.17	0.7
Age at menopause (years)	69,360	−0.12	5.4 × 10^−2^	−0.43	**3.5** × **10**^−**3**^	−0.17	**2.0** × **10**^−**3**^	−0.21	0.3
Telomere length (T/S)	37,684	0.18	9.7 × 10^−2^	1.80	0.7	−0.16	0.1	3.20	0.3

Units of the traits associated with quantitative measures are displayed within parentheses. *P*-values <0.05 marked in bold

Results from cross-trait LDSC genetic correlation and Mendelian randomization Egger regression (MR-Egger) analyses for IEAA and EEAA are presented. The traits are ordered by category (I) GWAS of anthropometric traits conducted by GIANT consortium, (II) GWAS of lipid, metabolic, and inflammatory outcomes and diseases, (III) GWAS of neurodegenerative and neuropsychiatric disorders, (IV) cognitive functioning and educational attainment traits, and (V) longevity, reproductive aging and mitotic clock related traits. Complete results are presented in Supplementary Tables 12, 13 and 14. We list the sample size of a study trait, Genetic correlation (*r*_g_) and its *P-*value (${P}_{{r}_{\rm {g}}}$) as well the estimate of causal effect (${\beta }_{{\rm {causal}}}$) and its *P*-value (${P}_{{\rm {causal}}}$) from MR-Egger regression

HDL = high-density lipoprotein, IBD = inflammatory bowel disease, AMD (subtype) = age-related macular degeneration (geographic atrophy), Edu. attainment = educational attainment, telomere length refers to leukocyte telomere length

### Blood cell composition vs. variants in *TERT*

By definition, IEAA is independent of various blood cell count estimates, i.e., it does not correlate with imputed blood cell abundance measures (Methods section). By contrast, LTL exhibits the expected positive correlation with the abundance of naive CD8^+^ T cells (*r* = 0.22, *P* = 4.6 × 10^−10^) and naive CD4^+^ T cells (*r* = 0.13, *P* = 0.00026) even after adjusting for chronological age (Supplementary Fig. 14). Consistent with our paradoxical association between LTL-related SNPs and IEAA, we find that *TERT* variants associated with a higher abundance of naive T cells (indicative of a younger adaptive immune system) are positively associated with higher values of IEAA and higher values of EEAA (indicative of an older epigenetic age, Supplementary Figs. 15 and 16, and Supplementary Data 4). However, none of the SNPs in the *TERT* locus was significantly associated with cell counts after correcting for multiple comparisons (uncorrected *P* > 0.003, Supplementary Data 4) despite of the large sample size *N* > 5000.

### hTERT is required for DNAm aging in human primary cells

As we were unable to functionally link IEAA to *TERT* through *cis*-eQTL analysis at 5p15.33, we examined the effects of experimentally induced hTERT expression on IEAA in a primary human cell culture model. We introduced a *TERT-*expressing vector or empty vector (as control) into primary fibroblasts isolated from human neonatal foreskin. Transduced *TERT*-expressing and non-*TERT* cells were cultured in parallel. Upon reaching confluence, the cells were collected, counted, seeded into fresh plates, and profiled using the Illumina Infinium 450K DNA methylation array.

While non-*TERT* cells senesced after ~150 days, *TERT*-expressing cells continued to proliferate unabated at a constant rate with time in culture (Fig. 3c). Single-time point analyses (Fig. 3d) showed that *TERT*-expressing cells exhibited a linear relationship between time in culture and the Horvath estimate of DNAm age (equivalent to a DNAm age of 50 years at 150 days), whereas in non-*TERT* cells DNAm age plateaued (equivalent to a DNAm age of 13 years) in spite of continued proliferation to the point of replicative senescence. Notably, DNAm age did not increase in *TERT*-expressing cells that received regular media change but were not passaged throughout the entire observation period of 170 days (right most bar in Fig. 3d). These cells were not senescent, given that their subsequent passaging resulted in normal proliferation. In multivariable regression analysis, the associations of DNAm age with cell passage number and cell population doubling number were highly modified by *TERT*-expression (*P*-interaction: *P* = 1.6 × 10^−6^ and *P* = 4.0 × 10^−5^, respectively; Supplementary Table 9). In the absence of *TERT*-expression, DNAm age did not increase with cell passage number, cell population doubling number, or time in culture.

### Other putative determinants of epigenetic age acceleration

To systematically elucidate possible further biological processes that influence epigenetic age acceleration, we tested our full genome-wide association statistics for IEAA and EEAA using a number of approaches. First, we used MAGENTA^29^ (Methods section) to identify biological pathways that are enriched for genes that harbor associated variants. For IEAA, nuclear transport (FDR = 0.017), Fc epsilon RI signaling (FDR = 0.027), and colorectal cancer processes (FDR = 0.042) were implicated. For EEAA, mRNA elongation (FDR = 0.011), mRNA transcription (FDR = 0.018), and neurotrophin signaling pathway (FDR = 0.042) were implicated (Supplementary Table 10). The GO gene set involved in telomere maintenance showed nominally significant enrichment with IEAA (*P* = 0.04, Supplementary Table 11), which is consistent with our results surrounding the overlap between IEAA and telomere length associated genes.

Second, we explored the genetic correlations (*r*_g_) between IEAA or EEAA and 27 phenotypes using LD score regression analysis of summary level GWAS data^30^ (Methods section and Fig. 1g). We observed moderate positive genetic correlation between IEAA and EEAA (*r*_g_ = 0.5, $P_{r_{\rm g}}$ = 8.9 × 10^−3^). IEAA showed weak positive genetic correlations with central adiposity (waist circumference) and metabolic disease-related traits, and EEAA showed stronger positive genetic correlations with central adiposity (*r*_g_ = 0.15 with waist circumference, *P* = 9.0 × 10^−4^) and metabolic disease-related traits (Table 4 and Supplementary Table 12). IEAA and EEAA also showed modest inverse genetic correlations with age at menopause.

Further, we performed a genetic correlation analysis using all available GWAS summary data from the LD Hub platform^31^ (see URL), identifying additional traits at nominal significance levels (*P* < 0.05). Lung function measures (forced expiratory volume) and brain volume measures exhibited negative genetic correlations with both IEAA and EEAA (Supplementary Data 5). Further, IEAA exhibited a positive genetic correlation *r*_g_ with primary sclerosing cholangitis and a negative genetic correlation with father’s age at death.

Third, we performed MAGENTA based hypergeometric analyses to test whether the top 2.5 and 10% of genes enriched for GWAS associations with IEAA or EEAA overlap with the top enriched genes for a range of complex traits (Methods section). This analysis suggested several additional possible genetic overlaps, including Huntington disease onset^32^ and bipolar disorder with IEAA, schizophrenia with EEAA, and age-related dementia^20^ with both IEAA and EEAA (Supplementary Data 6).

Finally, all the study traits were tested using MR-Egger regression which, by modeling the reported top genetic signals for each candidate trait, estimates the likely causal influence of that trait on IEAA or EEAA^28^ (Methods section). Nominally significant causal relationships on higher IEAA and EEAA were found for low-density lipoprotein (LDL) and total cholesterol levels (*P* < 0.05, Supplementary Tables 13 and 14) and for triglyceride levels on IEAA (*P* = 3.0 × 10^−2^, Table 4). Earlier menarche and menopause were associated with higher IEAA; each 1-year earlier age at menarche was associated +1.03 years higher IEAA (*P* = 4.1 × 10^−3^) and each 1-year earlier age at menopause was associated +0.43 years higher IEAA (*P* = 3.5 × 10^−3^) (Table 4). A sensitivity analysis shows that the observed causal associations do not result from instrumental variable SNPs co-locating with CpG sites from DNAm age estimators (Methods section and Supplementary Table 15). Our MR-Egger analysis revealed a causal effect of IEAA on a rough proxy of life span (father’s age at death) (*P* = 1.9 × 10^−2^, Supplementary Table 16), which is consistent with previous studies that demonstrated that epigenetic age acceleration predicts life span^3–7^.

## Discussion

This large genomic study provides several insights into the regulation of epigenetic aging, including apparently opposing roles for *TERT* on DNAm age and LTL. *TERT* encodes the catalytic subunit of telomerase, which counters telomere shortening during cell division^33^. *TERT* also possesses activities unrelated to telomere maintenance, such as in DNA repair, cell survival, protection from apoptosis or necrosis, stimulation of growth^34–36^ and cell proliferation, possibly by decreasing p21 production^37^. Here we show an additional pleiotropic role of *TERT* on advancing cell intrinsic DNAm age during cell proliferation. Our findings provide an explanation for the previously reported rapid rate of DNAm aging during embryonic development and early postnatal life, which are stages of rapid organismal growth accompanied by high levels of *TERT* activity and cell division^38–40^.

The paradoxical finding that *TERT* alleles associated with longer telomeres are associated with higher IEAA is supported by (a) significant meta-analysis *P*-values, (b) validation in multiple large cohort studies, (c) in vitro support using primary fibroblasts, (d) consistent associations of other LTL associated genes (*OBFC1* and *MPHOSPH6*), and (e) consistent genetic associations with select leukocyte subsets (e.g., naive T cells). While the paradoxical finding cannot be disputed on scientific grounds, its biological interpretation remains to be elucidated. In our genetic studies, we found no evidence for a broader causal inter-relationship between telomere length and IEAA consistent with the lack of phenotypic association between these traits in our studies (WHI: *r* = −0.05, *P* = 0.16; FHS: *r* = 0.0, *P* = 0.99, ref. ^41^) and in previous reports^15,19^. The lack of a phenotypic association between LTL and IEAA indicates that the shared genetic influence (due to *TERT* and, to a lesser extent, *OBFC1* and *MPHOSPH6*) is dwarfed by environmental influences acting on these traits.

While critically short telomere length is a well-established trigger of replicative senescence^38,42–44^, the functional consequences of epigenetic aging are yet unknown. Our experimental data suggest that epigenetic aging is not a determinant or marker of cell replicative senescence, since *TERT*-expressing cells continued to proliferate unabatedly despite well-advanced DNAm age, and non-*TERT*-expressing cells exhibited no DNAm age increase even at days 120–170 when proliferation had ceased and the cells had become senescent. Rather, *TERT* expression appears to allow cells to record their proliferation history. Our experiments show a clear association between DNAm age with cumulative population doubling (and days in culture) of cells with experimentally induced hTERT expression. This was not the case with proliferating cells that do not express hTERT. While the mechanism is yet to be elucidated, preliminary evidence suggests that DNAm age does indeed track the cell division of stem/progenitor cells, e.g., passage number is highly correlated with DNAm age of mesenchymal stem cells^2,45^.

Large-scale cross-sectional cohort studies have previously reported associations between epigenetic age acceleration (IEAA and EEAA) and body mass index and measures of insulin resistance^18^. Our genetic correlation analyses (between the measures of age acceleration and complex phenotypes) indicate that some of these associations may arise in part from shared genetic variants. The genetic correlation between IEAA and EEAA is quite high (*r*_g_ = 0.52, *P* = 0.0089) considering that these epigenetic biomarkers are based on distinct sets of CpGs and exhibit only modest phenotypic correlations (*r* = 0.37 in the WHI and *r* = 0.36 in the FHS). By contrast, IEAA/EEAA exhibit only relatively weak genetic correlations with many age related traits for a variety of reasons, including (i) technical variation/noise in the measures of epigenetic age acceleration, (ii) relatively low sample size in our GWAS of IEAA/EEAA, (iii) blood methylation data only provide insufficient information for capturing the dysfunction of other organ systems. The latter point is illustrated by the finding that obesity has a strong effect on the epigenetic age of liver tissue but only a weak effect on the epigenetic age of blood tissue^13,18^.

Our Mendelian randomization analyses provided tentative evidence for causal influences for blood lipid levels, but not for adiposity, on IEAA and EEAA. The suggestive causal effect of educational level on EEAA and the negative genetic correlation (*r* = −0.13, *P* = 1.0 × 10^−3^) are consistent with the previously observed phenotypic correlation between these traits^18^.

Finally, we found evidence for causal influences of earlier ages at menarche and menopause on higher IEAA. The directionally concordant influences of menarche and menopause, which signal the onset and cessation, respectively, of reproductive capacity, together with the lack of influence of any measure of adiposity on IEAA, suggest an effect of some yet identified driver of reproductive aging on DNAm aging. These findings, which suggest that sex steroids affect epigenetic aging, are consistent with previously reported associations regarding early menopause timing and higher IEAA in blood^16^. While menopausal hormone therapy was not found to be associated with IEAA in blood, it was found to be associated with younger epigenetic age acceleration in buccal epithelium^16^. The effect of menopause is consistent with reported anti-aging effects of sex hormone therapy on buccal cells and the pro-aging effect of surgical ovariectomy in blood^16^. Early age at menarche, a widely studied marker of the timing of puberty in females, is associated with higher risks for diverse diseases of aging^46^. Our findings indicate epigenetic aging as a possible intrinsic mechanism that underlies the recently described link between menarche age-lowering alleles and shorter lifespan^47^.

## Methods

### GWAS Cohorts

GWAS meta-analysis was performed on 9907 individuals across 15 studies (Supplementary Table 1) coming from eight cohorts: Framingham Heart Study (FHS), TwinsUK, Women’s Health Initiate (WHI), European Prospective Investigation into Cancer–Norfolk (EPIC-Norfolk), Baltimore Longitudinal Study of Aging (BLSA), Invecchiare in Chianti, aging in the Chianti Area Study (inCHIANTI), Brisbane Systems Genetics Study (BSGS), and Lothian Birth Cohorts of 1921 and 1936 (LBC) (Supplementary Note 1). Eleven data sets comprised individuals of European ancestry (EUR, 84.7%) and four data sets comprised individuals of African ancestry (AFR, 10.3%) or Hispanic ancestry (AMR, 5.0%). Age range was 10–98 years (69% females).

### DNA methylation age and measures of age acceleration

By contrasting the DNAm age estimate with chronological age, we defined measures of epigenetic age acceleration that are uncorrelated with chronological age. We evaluated two types of measures of epigenetic age acceleration in blood: cell-intrinsic and extrinsic epigenetic measures, which are independent of, or related to blood cell counts, respectively. Intrinsic epigenetic age acceleration (IEAA) is defined as the residual resulting from regressing the DNAm age estimate from Horvath (353 CpG markers) on chronological age and blood cell count estimates. By definition, IEAA does not depend on blood cell counts. By contrast, extrinsic epigenetic age acceleration (EEAA) depends on blood cell counts because it is defined by up-weighting the blood cell count contributions to the Hannum’s epigenetic age estimator (71 CpGs). Thus, EEAA captures both age-related changes in blood cell types, as well as cell-intrinsic age-related changes in DNAm levels.

IEAA and EEAA are based on the DNAm age estimates from Horvath^2^ (353 CpG markers) and from Hannum et al.^1^ (71 CpGs), respectively. Mathematical details and software tutorials for estimating epigenetic age can be found in Horvath^2^. By definition, IEAA is independent of blood immune cell counts (naive CD8^+^ T cells, exhausted CD8^+^ T cells, plasma B cells, CD4^+^ T cells, natural killer cells, monocytes, and granulocytes) estimated from DNA methylation data. EEAA is calculated using the following three steps. First, we calculated the DNAm age estimate from Hannum et al.^1^. Second, we increased the contribution of age related blood cell types to the DNAm age estimate by forming a weighted average of Hannum’s DNAm age estimate with the following 3 blood cell type estimate: naive (CD45RA^+^CCR7^+^) cytotoxic T cells, exhausted (CD28^−^CD45RA^−^) cytotoxic T cells, and plasmablasts using the Klemera-Doubal approach^48^. The weights used in the averaging procedure were chosen on the basis of the WHI data, i.e., the same weights were used in all data sets. EEAA is positively associated with exhausted CD8^+^ T cells, plasmablast cells, and negatively associated with naive CD8^+^ T cells. Thus, EEAA tracks both age related changes in blood cell composition and cell-intrinsic DNAm changes. Both the intrinsic and extrinsic DNAm age measures were correlated highly with chronological age within each contributing cohort (0.63 ≤ *r* ≤ 0.97, Supplementary Table 1), except for the two Lothian birth cohorts whose participants were born in one of two single years and hence had a small age range at testing. Conversely, by definition, our measures of DNAm age acceleration, IEAA and EEAA, are not associated with chronological age.

The measures of epigenetic age acceleration are implemented in our freely available software (https://dnamage.genetics.ucla.edu)^2^.

### Estimating blood cell counts based on DNA methylation levels

The blood cell abundance measures were estimated using two different methods. The proportions of cytotoxic (CD8^+^) T cells, helper (CD4^+^) T, natural killer, B cells, and granulocytes were estimated with the Houseman method^49^. The percentage of exhausted CD8^+^ T cells (defined as CD28^−^CD45RA^−^), the number (count) of naive CD8^+^ T cells (defined as CD45RA^+^CCR7^+^), and plasma blasts were estimated with the Horvath method^14^. These DNAm based estimates of blood cell counts are highly correlated with corresponding flow cytometric measures^50^.

### Heritability analysis

Both IEAA and EEAA measures were adjusted for sex prior to estimation of heritability. We conducted heritability analysis using three approaches: (1) SOLAR (Sequential Oligogenic Linkage Analysis Routine)^51^ based on pedigree data, (2) LDSC (LD score regression)^30^ based on GWAS summary data, and GCTA (Genome-wide Complex Trait Analysis)^52^ based on SNP array data to estimate narrow sense *h*^2^. For the Framingham heart pedigree offspring cohort, we used the additive polygenic model implemented in SOLAR to estimate the heritability of IEAA and EEAA. In this additive polygenic model, heritability is defined as the proportion of phenotypic variance attributable to genetic variation. To estimate the heritability of IEAA and EEAA using the meta-analysis results from all European individuals from stage 1 (Fig. 1a) (studies 1–11, *N* = 8393), we used LDSC analysis because it only requires GWAS summary statistics rather than genotype type data. We chose the following options for LD score regression: 1000 Genome European data (phase I) downloaded from LDSC for specifying independent variables (--ref-ld flag) and weights (--w-ld flag) in the regression. Prior to estimation, we filtered our markers to HapMap3 markers as downloaded and suggested by LDSC, which could help align allele codes of our GWAS results with other GWAS results for the genetic correlation analysis conducted later.

GCTA—REML analysis was used to estimate the heritability of IEAA and EEAA among the WHI participants of African (AFR) or Hispanic ancestry. The WHI sub-studies were genotyped on different platforms (Supplementary Table 2). In order to combine the genotype data across the studies from WHI EMPC and WHI BA23, we converted the MaCH dosage format into PLINK format and used both genotyped and imputed markers in the analysis. We only used SNP markers that could be found in all studies. We focused on high quality SNPs defined based on minor allele frequency (MAF) > 0.05, Hardy–Weinberg equilibrium (HWE) *P* > 0.0001, and MaCH *r*^2^ > 0.8, yielding ~4 million markers. In particular, we increased the threshold of MAF to 10% in estimating the heritability of the Hispanic group in order to ensure the convergence of the REML estimation procedure. All analyses were adjusted for 4 principal components (PC).

### GWAS meta-analysis

Our GWAS meta-analysis involved ~7.4 million SNPs or INDEL variants, which were genotyped and imputed markers with the 1000 genomes haplotype reference panel. Prior to imputation, SNP quality was assessed by MAF, HWE, and missingness rates across individuals (Supplementary Table 4). The individual studies used IMPUTE2^53^ with haplotypes phased using SHAPEIT^54^ or MaCH^55^ phased using Beagle^56^ or Minimac^53^ to impute SNP and INDEL markers based on the 1000 Genomes haplotypes released in 2011 June or 2012 March. The quality of imputed markers was assessed by the Info measure >0.4 (in IMPUTE2) or *R*^2^ > 0.3 (in Minimac), and HWE *P* > 1.0 × 10^−6^. To increase resolution for SNP association, a few genomic regions in the FHS cohort were also imputed based the Haplotype Reference Consortium (*N* = 64,976)^57^. FHS used linear mixed models to account for pedigree structure via a kinship matrix, as implemented in R “lmekin” package. The BSGS cohort used Merlin/QTDT^58^ for family-based association analysis. For other association analyses, we regressed the age acceleration trait values on estimated genotype dosage (counts of test alleles) or expected genotype dosage, implemented in Mach2QTL^59^, SNPTEST^60^, and PLINK. All association models were adjusted for sex, to account for the higher epigenetic age acceleration in men than women^50^, and also for PCs as needed. We included variants with MAF ≥ 2%. SNPs were removed from an individual study if they exhibited extreme effects (absolute regression coefficient *β* > 30, Supplementary Table 4).

We divided the meta-analysis into two since IEAA and EEAA differ across racial/ethnic groups^50^. In one arm, we performed GWAS meta-analysis of IEAA and EEAA, focusing on individuals of European ancestry (*N* = 8393, studies 1–11 in Supplementary Table 1). We required a marker present in at least 5 study data sets and combined the coefficient estimates *β* from each study using a fixed-effects meta-analysis model weighted by inverse variance, as implemented in the software Metal^61^. In the other arm, each SNP with suggestive association (*P* < 1.0 × 10^−5^) in Europeans was subsequently evaluated in individuals of non-European ancestry (*N* = 1514, studies 12–15 in Supplementary Table 1). A further meta-analysis combined the GWAS findings from the two ancestries. We removed SNPs from the meta-analysis if they exhibited highly significant heterogeneity across studies (Cochran Q *I*^2^ *P*-value ≤ 0.001), or co-located with CpG from the DNAm age predictors according to the Illumina annotation file for the Illumina Infinium 450K array. We analyzed additional SNPs across all study sets to arrive at summary statistics at the combined stage, which were needed for our summary statistics based Mendelian randomization analyses. The quality of SNPs was also assessed using the Cochran Q *I*^2^ *P*-value.

### Linkage disequilibrium analysis

Regional SNP association results were visualized with the software LocusZoom^62^. All linkage disequilibrium (LD) estimates presented in this article were calculated using individuals of European ancestry from the 1000 genomes reference panel (released in Oct 2012).

### Conditional analyses

The conditional analysis implemented in GCTA software^52^ was used to test whether a given genetic locus harbored multiple independent causal variants. We conditioned on the leading SNP with the most significant meta-analysis *P*-value (Table 1). As reference panel for inferring the LD pattern we used the *N* = 379 individuals with European ancestry from the 1000 genomes panel released in December 2013. We defined a SNP as having an additional association if it remained significant (*P* < 5 × 10^−8^) after conditioning on the leading SNP and also met the additional criterion ${\rm log}_{10}{\rm BF} \ge 6$ for a significant trans-ethnic association.

### Chromatin state annotations

For each leading SNP of a significant locus, we used the UCSC genome browser to display the primary chromatin states across 127 cell/tissue lines at 200 bp resolution (Supplementary Fig. 8). The *n* = 127 diverse cell or tissue lines were profiled by the NIH RoadMap Epigenomics^22^ (*n* = 111) and ENCODE projects^63^ (*n* = 16). We used a 15-state chromatin model (from ChromHMM) which is based on five histone modification marks^22^.

### Annotations for genome-wide significant variants

We used the HaploReg (version 4.1) tool^64^ to display characters of genome-wide significant variants including conserved regions by GERP and SiPhy scores, DNase tracks, involved proteins and motifs, GWAS hits listed in NHGRI/EBI and functional annotation listed in dbSNP database, as summarized in Supplementary Data 1.

### Leukocyte *cis*-eQTL analyses

To evaluate *cis*-eQTL in blood, our *cis*-eQTL study leveraged a large-scale blood expression data (*n* = 15,295) that came from five broad categories of data. The first category involved a large-scale eQTL analysis in 5257 individuals collected from the FHS pedigree cohort (of European ancestry)^65^. Linear mixed models were performed for the eQTL analysis, adjusted for family structure via random effects and adjusted gender, age, blood cell counts, PCs, and other potential confounders via fixed effects. The analysis was carried out using the “pedigreemm” package of R. The second category involved the significant *cis*-eQTL, released from GTEx (version 6 in 2015)^66^. The expression data from GTEx involve multiple tissues from 449 individuals of mostly (>80%) European ancestry. We used the *cis*-eQTL results evaluated in 338 blood samples. The downloaded *cis*-eQTL results only list significant results (FDR *q* < 0.05), according to a permutation test based threshold that corrected for multiple comparisons across genes and tissue types. The third category involved the *cis*-eQTL results from LSMeta^67^_,_ which was a large-scale eQTL meta-analysis in 5331 blood samples collected from 7 studies including our study cohort inChianti. We downloaded the *cis*-eQTL results from http://genenetwork.nl/bloodeqtlbrowser/. The fourth and fifth categories were discovery and replication samples from an eQTL analysis in peripheral blood^68^, respectively. The publicly released *cis*-eQTL results only involved the 9640 most significant (FDR *q* < 0.01) *cis*-eQTL results (corresponding to 9640 significant genes) from the discovery sample. The fourth category involved the expression data of 2494 twins from the NTR cohort. The fifth category involved 1895 unrelated individuals from the NESDA cohort.

For all five categories of blood data, the *cis*-window surrounding each SNP marker was defined as ±1 Mb. We defined a significant *cis*-eQTL relationship by imposing the following criteria: (a) FDR *q* < 0.05 for categories 1–3, (b) FDR *q* < 0.01 for categories 4 and 5.

### SMR analysis

SMR^23^ uses SNPs as instrumental variables to test for a direct association between an exposure variable and an outcome variable, irrespective of potential confounders. Unlike conventional Mendelian randomization analysis, the SMR test uses summary-level data for both SNP-exposure and SNP-outcome that can come from different GWAS studies^23^. We tested the expression levels of the eleven candidate genes identified in our leukocyte *cis*-eQTL analysis.

SMR defines a pleiotropic association as association between gene expression and a test trait due to pleiotropy or causality (Supplementary Fig. 12a, b). A significant SMR test *P*-value does not necessarily mean that gene expression and the trait are affected by the same underlying causal variant, as the association could possibly be due to the top associated *cis*-eQTL being in LD with two distinct causal variants. Zhu et al.^23^ define the scenario of several causal variants, which is of less biological interest than pleiotropy, as “linkage” and proposed a statistical test “HEIDI” for distinguishing it from pleiotropy. The null hypothesis of the HEIDI test corresponds to desirable causal scenarios. Thus, a non-significant *P*-value (defined here as *P* ≥ 0.01) of the HEIDI test is a desirable finding. Conversely, a significant HEIDI test *P*-value indicates that at least two linked causal variants affect both gene expression and epigenetic age acceleration (Supplementary Fig. 12c).

To test the association of a given gene expression with age acceleration, we used summary level *cis*-eQTL results from (1) FHS, (2) GTEx, and (3) LSMeta (total: *N* = 10,906).

We included the *cis*-SNPs (with MAF ≥ 0.10) within a test gene (±1 Mb). We selected the *cis*-SNPs as instrumental markers as follows: *cis*-eQTL FDR < .05 for GTEx, *cis*-eQTL *P* = 1 × 10^−6^ for the two large-scale studies: LSMeta and FHS. All significant SNP-gene pairs were subjected to the HEIDI analysis. The analysis involves the summary data of the *cis*-SNPs surrounding the instrumental markers and the LD pattern evaluated from a reference panel. We used the 1000 genome individuals with ancestry of European (*N* = 379) released in December 2013 as the reference panel, imposed an LD threshold of 0.9 and selected a default setting based on a chi-square ($\chi _1^2$) test statistic threshold of 10 for SNP pruning. The GWAS summary data were based on the meta-analysis results at the combined stage. As a sensitivity check, we repeated the HEIDI analysis using the summary GWAS data of individuals with European ancestry (studies 1–11).

### Mendelian randomization analysis for LTL and IEAA

We gathered the summary statistics from three large-scale meta-analysis studies for association with LTL, including (I) the association results of 484 SNPs located in *TERT* locus from the study conducted by Bojesen et al.^25^ (*N* = 53,724 individuals of European ancestry), (II) the GWAS summary data from the study conducted by Codd et al.^26^ (*N* = 37,684 individuals of European ancestry) downloaded from the European Network for Genetic and Genomic Epidemiology consortium (ENGAGE, see URL) and (III) the association results of 4 SNPs at *P* < 5.0 × 10^−8^ and 1 SNPs at *P* < 1.0 × 10^−6^ listed in Table 1 from the study conducted by Pooley et al.^27^ (*N* = 26,089 individuals of European ancestry). In the first study, the effect sizes were available for change in telomere length (ΔTL > 0 indicating a test allele associated with longer LTL) and fold change in telomere length (>1 indicating a test allele associated with longer LTL). We used the effect sizes with respect to ΔTL in our analysis. The other two studies reported ΔTL in their summary data. All telomere lengths refer to the relative telomere to single-copy gene (T/S) ratios using quantitative PCR methods with different scaling approaches applied to each study. The summary data of the second were used for bidirectional Mendelian randomization analysis. Summary statistics of IEAA and EEAA were based on the association results from the combined meta-analysis. Our SMR analysis used the summary data from studies I and II. To compare the patterns between LTL associations and IEAA associations at 5p15.33 *TERT* locus, we used the dense panel of SNP association results from study I (484 SNPs), as depicted in Fig. 3b.

### IEAA adjusted LTL in 5p15.33 *TERT* locus

To inspect the SNPs on *TERT* locus affecting IEAA independent of LTL, we re-conducted the association analysis on a subset (*N* = 785) individuals from the WHI for whom both DNA methylation data and leukocyte telomere length measures were available. The study involved 457 individuals with European ancestry (from studies 3 and 5) and 328 individuals with Africans ancestry (from study 12). For each study, association tests were performed on IEAA and IEAA adjusted LTL, respectively. The results were combined by fixed-effects meta-analysis models.

### Blood cell composition vs. variants in *TERT*

The imputed blood cell abundance measures were related to SNPs in the *TERT* locus using 8 studies from two cohorts: FHS (study 1) and WHI (studies 3–5 and 12–15, listed in Supplementary Table 1) involving *n* = 5373 individuals. The following imputed blood cell counts were analyzed: naive CD4^+^ T, naive CD8^+^ T, exhausted CD8^+^ T cells (defined as CD28-negative CD45R-negative), plasmablasts, CD4^+^ T, nature killer cells, monocytes, and granulocytes. Blood cell proportions (CD8^+^ T cells, CD4^+^ T cells, NK cells, B cells, and granulocytes) were estimated using Houseman’s estimation method which is based on DNA methylation signatures from purified leukocyte samples^49^. The percentage of exhausted CD8^+^ T cells (defined as CD28^−^CD45RA^−^ B cells) and the number of naive CD8^+^ T cells (defined as CD45RA^+^CCR7^+^ B cells) were estimated using the advanced analysis option of the epigenetic clock software^14^. To avoid confounding, the cell abundance measures were adjusted for chronological age by forming residuals. For each study, the SNP association analysis was performed using the same SNP makers from our genome-wide meta-analysis of IEAA and EEAA. The results were combined across studies using the same fixed-effects meta-analysis model.

### In vitro studies of hTERT in human fibroblasts

Foreskins were obtained from routine elective circumcision. The tissue was cut into small pieces and digested overnight at 4 °C with Liberase, after which the epidermis was peeled away from the dermis. Several pieces of dermis were placed face down in a plastic dish with DMEM supplemented with 10% foetal calf serum, penicillin, streptomycin, and gentamycin. After incubation at 37 °C with 5% carbon dioxide for a week, fibroblasts that emerged from the tissues were collected and expanded in fresh dishes.

Recombinant retroviruses bearing empty vector (pBabePuro) or the hTERT gene; pBabePurohTERT (kind gift from Robert Weinberg) were prepared by transfecting Phoenix A cells grown in DMEM supplemented with 10% foetal calf serum, using Profection Mammalian Transfection System (Promega Cat. No:E1200). Forty-eight hours post-transfection, recombinant viruses in the media were collected, filtered through 0.2 micrometer filter and mixed with Polybrene (Santa Cruz Biotechnology Cat. No: 134220) to a final concentration of 8 µg/ml. Media of primary fibroblasts were removed and replaced with the virus mix. After 24 h at 37 °C (5% carbon dioxide), the virus mix was removed and fresh media (DMEM +10% foetal calf serum) containing 1 µg/ml puromycin was added to kill uninfected cells. Puromycin selection was stopped after 4 days post-selection, when all cells in the un-infected control plates were killed. Successfully infected fibroblast were collected, counted and 200,000 were seeded into 10 cm cell culture plates containing DMEM supplemented with 10% foetal calf serum. When cells became confluent, they were collected by trypsinisation, counted and 200,000 were seeded in fresh 10 cm plates. This cycle of culture, collecting and seeding was repeated until the control cells senesced. The cell numbers were used to calculate population doubling according to the following formula: Population doubling = 3.32[log(final cell number)—log(number of cell seeded)]. In parallel to the proliferating culture of cells described above, separate plates of infected fibroblasts (pBabePuro and pBabePurohTERT) were grown without passaging throughout the experiment, but with regular media change three times a week for the duration of the experiment. These were regarded as “static” cultures as they were not passed since being seeded from the start of the experiment. DNA from all the collected cells were extracted according to the procedure described in the QIAamp DNA Mini Kit (Qiagen Cat. No. 51306). The DNA was subjected to DNA methylation analyses using the Illumina 450 arrays.

### GWAS based enrichment analysis with MAGENTA

We used the MAGENTA software^29^ to assess whether our meta-analysis GWAS results of epigenetic age acceleration are enriched for various gene sets, e.g., KEGG pathways, Gene Ontology (GO) terms, such as biological processes or molecular functions. To assign genes to SNPs, we extended gene boundaries to ±50 kb. For computational reasons, we removed categories that did not contain any genes related to age acceleration at a level of *P* < 1.0 × 10^−3^ or that contained fewer than 10 genes. The cutoffs of gene set enrichment analysis (GSEA) in the MAGENTA algorithm were set at 95th and 75th percentiles which are the default parameter values for a general phenotype and for a highly polygenic trait, respectively^29^.

Initially, empirical *P*-values were estimated based on 10,000 permutations. For significant gene sets (empirical *P* < 1.0 × 10^−4^), we estimated the final empirical *P-*value using one million permutations. We only report gene sets whose false discovery rate FDR (calculated by MAGENTA) was <0.20.

### LDSC genetic correlation analysis

We performed cross-trait LD score regression^30^ to relate IEAA/EEAA to various complex traits (27 GWAS summary data across 23 distinct phenotypes). The GWAS results for IEAA and EEAA were based on the summary data at stage 1 analysis.

The following is a terse description of the 27 published GWAS studies. Two GWAS results in individuals of European ancestry came from the GIANT consortium on body fat distribution: waist circumference and hip to waist ratio. GWAS results of BMI and height also from the GIANT consortium. Further, we used published GWAS results from inflammatory bowel disorder (IBD) and its two subtypes: Crohn’s disease and ulcerative colitis, lipid levels, metabolic outcomes and diseases: insulin and glucose levels, type 2 diabetes (stage 1 results) phenotype, age-related macular degeneration (neovascular and geographic atrophy), Alzheimer’s disease (stage 1 results), attention deficit hyperactivity disorder (ADHD), bipolar disorder, major depressive disorder, schizophrenia, education attainment, age at menarche, age at menopause, LTL and longevity. The summary data of LTL was based the GWAS conducted by Codd et al.^26^, as described in an earlier section. A description of other published GWAS study can be found in Supplementary Note 4.

As recommend by LDSC, we filtered to HapMap3 SNPs for each GWAS summary data, which could help align allele codes of our GWAS results with other GWAS results for the genetic correlation analysis. In our GWAS studies with GC-correction, we constrained the intercept terms with the --intercept–h2 and –intercept-gencov flags. Toward this end, we used the heritability analysis of the LDSC regression to estimate intercept terms. Additional genetic correlation analysis was performed via the LD Hub software tool^31^ that allowed us to analyze 233 traits. The built-in LDSC platform automatically removed all variants in the MHC region on chromosome 6 (26–34 MB).

### MR-Egger regression

Under a weaker set of assumptions than typically used in Mendelian randomization (MR), an adaption of Egger regression can be used to detect and correct for the bias due to directional pleiotropy^28^. While the standard method of MR estimation, two-stage least squares, may be biased when directional pleiotropy is present, MR-Egger regression can provide a consistent estimate of the causal effect of an exposure (e.g., age at menopause) on an outcome trait (e.g., epigenetic age acceleration). In testing the regression model, we used the leading variants (*P* < 5.0 × 10^−8^ or their surrogates) from each GWAS locus associated with the exposure, as instrumental variables. We performed LD-based clumping procedure in PLINK with a threshold of *r*^2^ set at 0.1 in a window size of 250 kb to yield the leading variants present in both GWAS summary data sets (for exposure and outcome), as needed. For those traits showing significant causal effects on IEAA (or EEAA), we performed a sensitivity analysis (stratified analysis) to check if the significant associations spuriously resulted from the instrumental variables (SNPs) co-locating (±1 Mb) with CpG sites from the DNAm age estimators. The random effects model meta-analysis was performed using “MendelianRandomization” R package.

### GWAS-based overlap analysis

Our GWAS-based overlap analysis related gene sets found by our GWAS of epigenetic age acceleration with analogous gene sets found by published GWAS of various phenotypes. We used the MAGENTA software to calculate an overall GWAS *P-*value per gene, which is based on the most significant SNP association *P-*value within the gene boundary (±50 kb) adjusted for gene size, number of SNPs per kb, and other potential confounders^29^. To assess the overlap between age acceleration and a test trait, we selected the top 2.5% (roughly 500 genes ranked by *P-*values) and top 10 % genes (roughly 1900 genes) for each trait and calculated one-sided hypergeometric *P*-values^20,69^. In contrast with the genetic correlation analysis, GWAS based overlap analysis does not keep track of the signs of SNP allele associations.

We performed the overlap analysis for all the 23 complex traits used in the genetic correlation LDSC analysis and a few more studies that we were not able to conduct the LDSC analysis due to small sample size (*N* < 5000), negative heritability estimates or the entire study population from non-European ancestry. The additional traits included modifiers of Huntington’s disease motor onset, Parkinson’s disease and cognitive functioning traits (Supplementary Note 4).

### URLs

URLs of databases and bioinformatics tools used throughout the masnucript are: 1000 genome project, http://www.1000genomes.org/, DNAm age, http://labs.genetics.ucla.edu/horvath/htdocs/dnamage/, ENGAGE, https://downloads.lcbru.le.ac.uk/, EIGENSTRAT, http://genepath.med.harvard.edu/~reich/Software.htm, GTEx, http://www.gtexportal.org/home/documentationPage#AboutGTEx, GIANT, https://www.broadinstitute.org/collaboration/giant/index.php/Main_Page, HaploReg, http://www.broadinstitute.org/mammals/haploreg/haploreg.php, HRC, http://www.sanger.ac.uk/science/collaboration/haplotype-reference-consortium, HRS, http://hrsonline.isr.umich.edu/, Illumina Infinium 450K array annotation, http://support.illumina.com/downloads/infinium_humanmethylation450_product_files.html.

IMPUTE2, https://mathgen.stats.ox.ac.uk/impute/impute_v2.html, LD score Regression, https://github.com/bulik/ldsc, LD Hub, http://ldsc.broadinstitute.org/ldhub/, Locuszoom, http://csg.sph.umich.edu/locuszoom/, METAL, http://csg.sph.umich.edu/abecasis/Metal/, MAGENTA, https://www.broadinstitute.org/mpg/magenta/, NESDA, http://www.nesda.nl/en/, NTR, http://www.tweelingenregister.org/en/, PLINK, http://pngu.mgh.harvard.edu/~purcell/plink/, R metafor, http://cran.r-project.org/web/packages/metafor/, R WGCNA, http://labs.genetics.ucla.edu/horvath/CoexpressionNetwork/, SHAPEIT, https://mathgen.stats.ox.ac.uk/genetics_software/shapeit/shapeit.html, SNPTEST, https://mathgen.stats.ox.ac.uk/genetics_software/snptest/snptest.html, YFS, http://youngfinnsstudy.utu.fi/index.html,

### Data availability

The FHS data are available at dbGaP under the accession numbers phs000342 and phs000724. The TwinsUK DNA methylation data are available through the NCBI Gene Expression Omnibus (GEO) under accession number GSE62992. Individual level genotype and phenotype data from TwinsUK are not permitted to be shared or deposited due to the original consent given at the time of data collection. However, genotype, DNA methylation, and phenotype data can be accessed through application to the TwinsUK data access committee (http://www.twinsuk.ac.uk/data-access/submission-procedure/). The WHI data are available at dbGaP under the accession numbers phs000200.v10.p3. The phenotypes of BLSA and inChianti data are available at dbGaP under the accession numbers phs000215.v2.p1. LBC methylation data have been submitted to the European Genome-phenome Archive under accession number EGAS00001000910; phenotypic data are available at dbGaP under the accession number phs000821.v1.p1. BSGS methylation data are available from GEO accession number GSE56105. Additional information can be found in Supplementary Note 1 and Supplementary Table 3.

## Electronic supplementary material

Supplementary Information

Description of Additional Supplementary Files

Supplementary Data 1

Supplementary Data 2

Supplementary Data 3

Supplementary Data 4

Supplementary Data 5

Supplementary Data 6
