# Supplementary material for: GWAS of epigenetic aging rates in blood reveals a critical role for TERT
Source: Nat Commun. 2018 Jan 26;9:387. doi: 10.1038/s41467-017-02697-5 (PMC5786029; doi:10.1038/s41467-017-02697-5)
Supplement: Supplementary file 1 — Supplementary Information [file 41467_2017_2697_MOESM1_ESM.pdf]

Supplementary Figures

Supplementary Figure 1: QQ plots for evaluating genomic inflation in the meta-analysis

The figure presents the QQ plots based on the GWAS results using blood tissues for (1) IEAA (on top) and (2) EEAA (on bottom). The association tests were performed using all the 11 studies of European ancestry for stage 1 analysis. The black and red lines represent the observed and expected meta P values, respectively. The genomic inflation factors were computed using all the SNPs (denoted by λ) and the SNPs limited with common variants with minor allele frequencies ≥ 5% (denoted by λ_{common}), respectively.

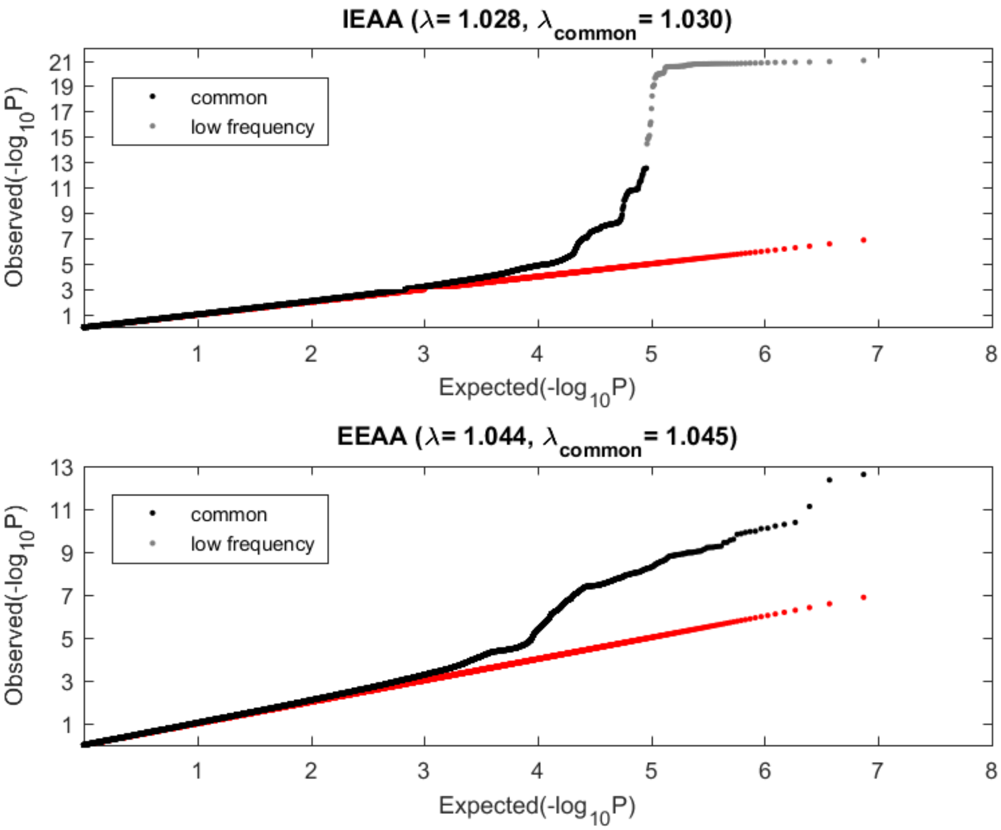

Supplementary Figure 2: Regional association results of susceptibility loci for IEAA

The plots display the association results surrounding the leading SNP at susceptibility locus for IEAA: (a) 3q25.33, (b) 6p22.3, (c) 6p22.2, and (d) 17q22. The colors visualize linkage disequilibrium (LD) r^2 between the leading marker and neighboring SNPs. In 6p22.3, we display both leading SNPs: rs14093668 (marked in red diamond) and rs6915893 (marked in blue diamond). The other SNPs are marked in red (blue) if they reveal higher LD with respect to rs14093668 (rs6915893).

(a) 3q25.33

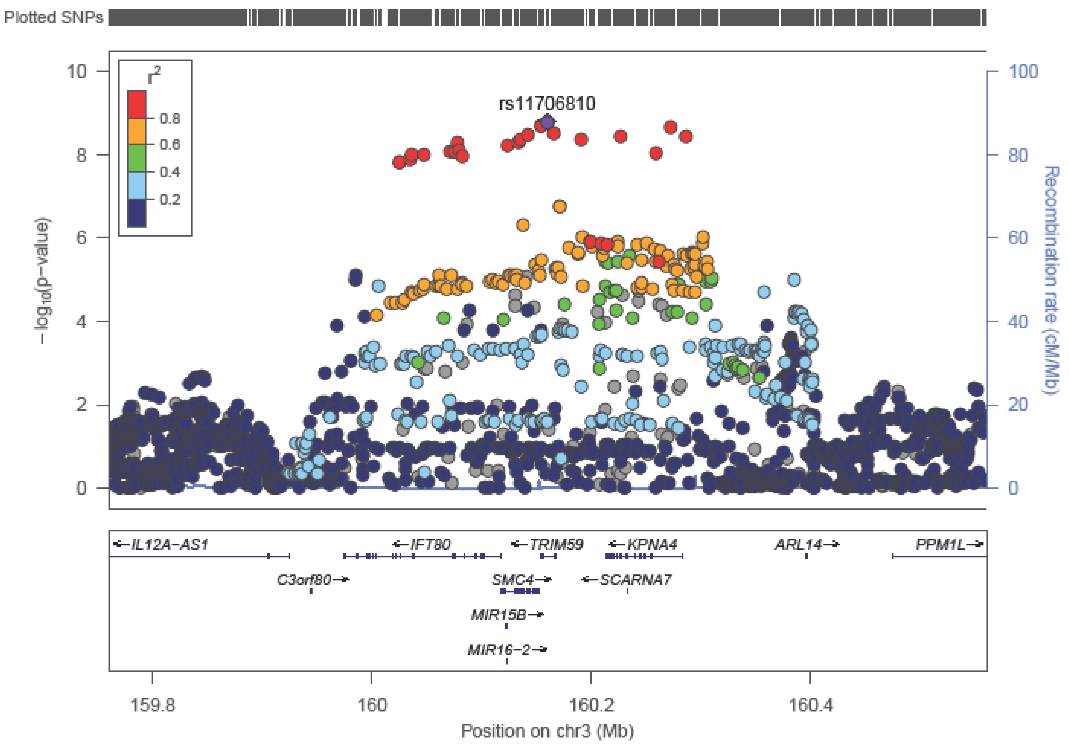

(b) 6p22.3

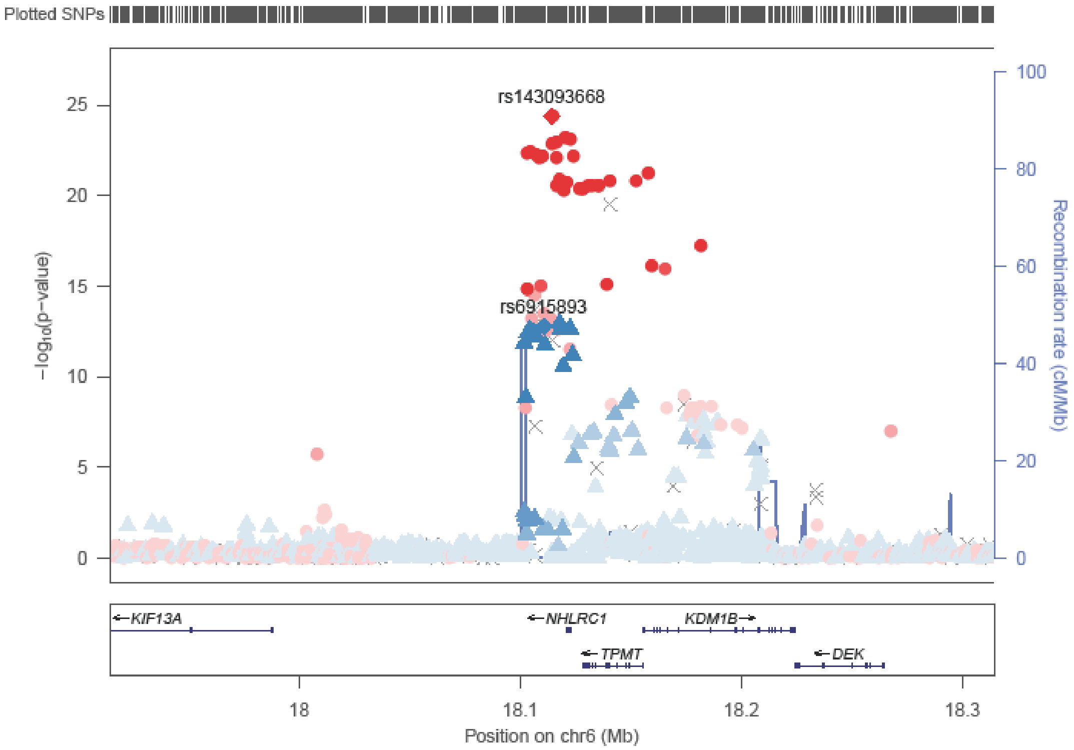

(c) 6p22.2

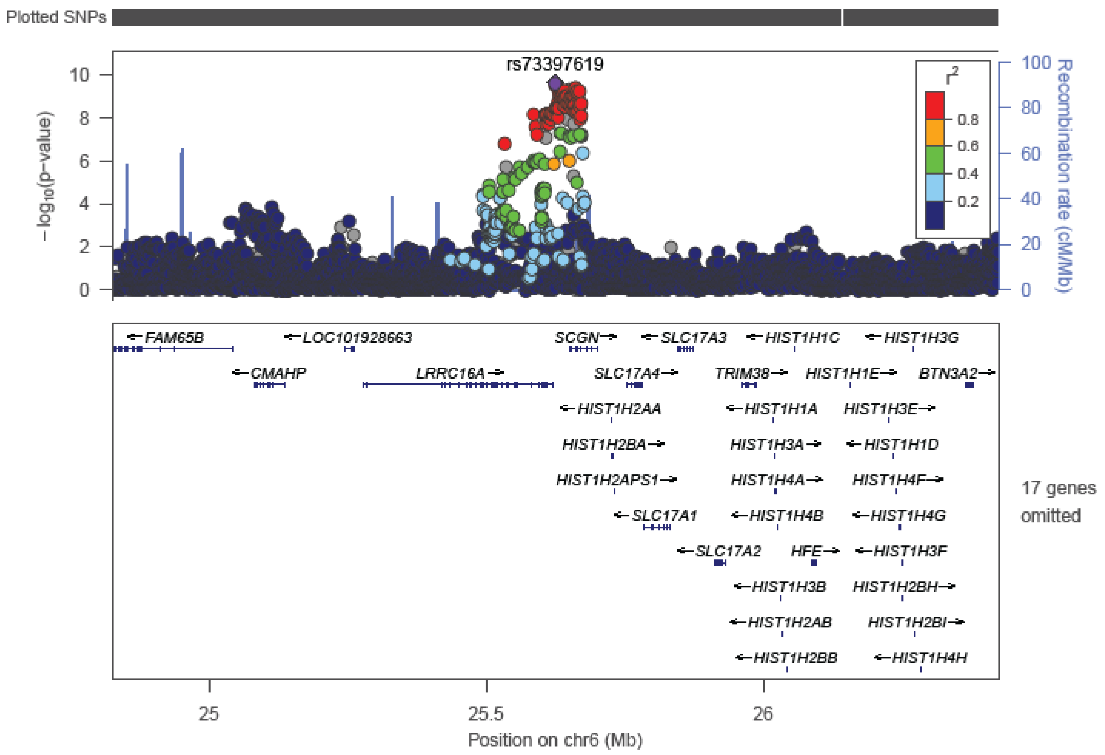

Plotted SNPs
rs73397619
r2
0.8
0.6
0.4
0.2
−log10(p−value)
Recombination rate (cM/Mb)
FAM65B
LOC101928663
SCGN
SLC17A3
HIST1H1C
HIST1H3G
CMAHP
LRRC16A
SLC17A4
TRIM38
HIST1H1E
BTN3A2
HIST1H2AA
HIST1H1A
HIST1H3E
HIST1H2BA
HIST1H3A
HIST1H1D
HIST1H2APS1
HIST1H4A
HIST1H4F
SLC17A1
HIST1H4B
HIST1H4G
SLC17A2
HFE
HIST1H3F
HIST1H3B
HIST1H2BH
HIST1H2AB
HIST1H2BI
HIST1H2BB
HIST1H4H
17 genes omitted
Position on chr6 (Mb)

(d) 17q22

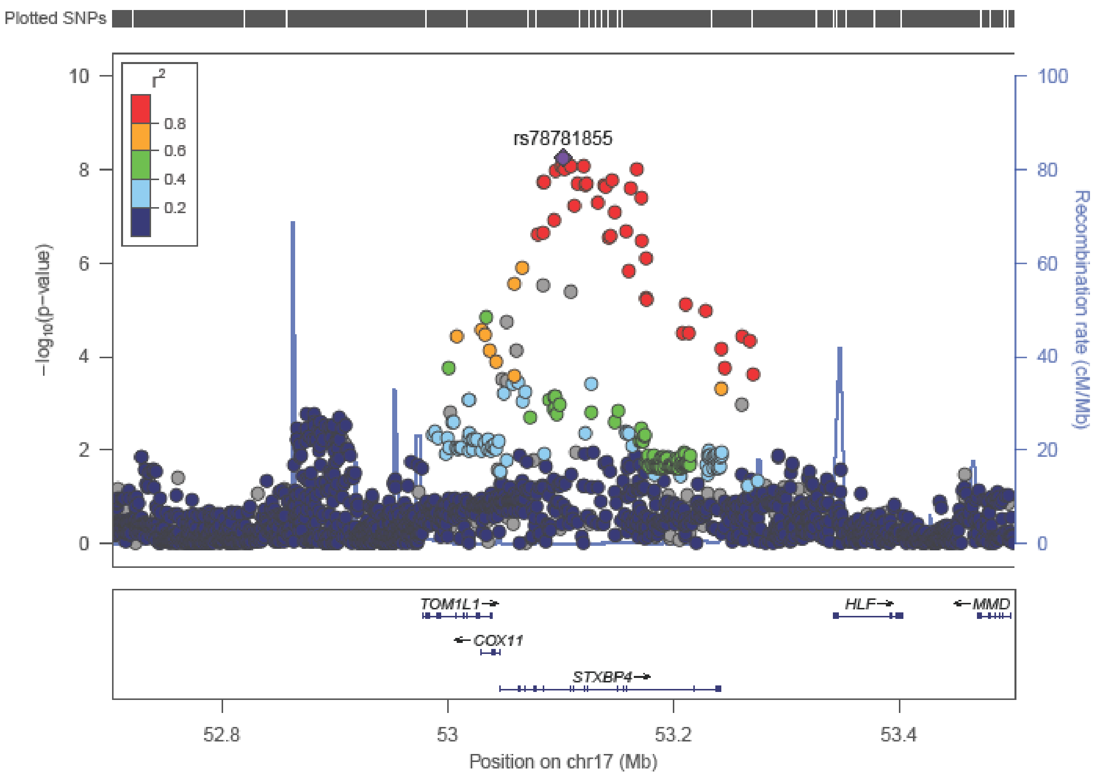

Plotted SNPs
rs78781855
r²
0.8
0.6
0.4
0.2
−log10(p-value)
Recombination rate (cM/Mb)
TOM1L1
COX11
STXBP4
HLF
MMD
52.8
53
53.2
53.4
Position on chr17 (Mb)

Supplementary Figure 3: GCTA conditional analysis for identifying multiple associations at IEAA susceptibility loci

The plots display the results from GCTA conditional analysis (a-e) using the GWAS summary data based on all studies. At each locus, the results present the association analysis conditional on the most significant SNPs: (a) rs11706830 in 3q25.33, (b) rs2736099 in 5p15.33, (c) rs143093668 in 6p22.3, (d) rs73397619 in 6p22.2, and (e) rs78781855 in 17q22. All the conditional association signals (marked in blue) are considerably lower than those in marginal associations except in the locus (c) 6p22.3, in which several SNPs remain association P values < 5.0E-08 (marked in green triangular), led by the SNP rs6915893 (marked in black triangular). We conducted a subsequent GCTA association analysis conditional on the two leading SNPs, rs143093668 and rs6915893, and confirmed that there was no additional leading markers in 6p22.3.

Similar patterns can be observed in the panels (f-j). The plots display the results from sensitivity analysis that we used the GWAS summary data based on studies 1-11 comprising individuals of EUR ancestry in the GCTA analysis.

(a-e)

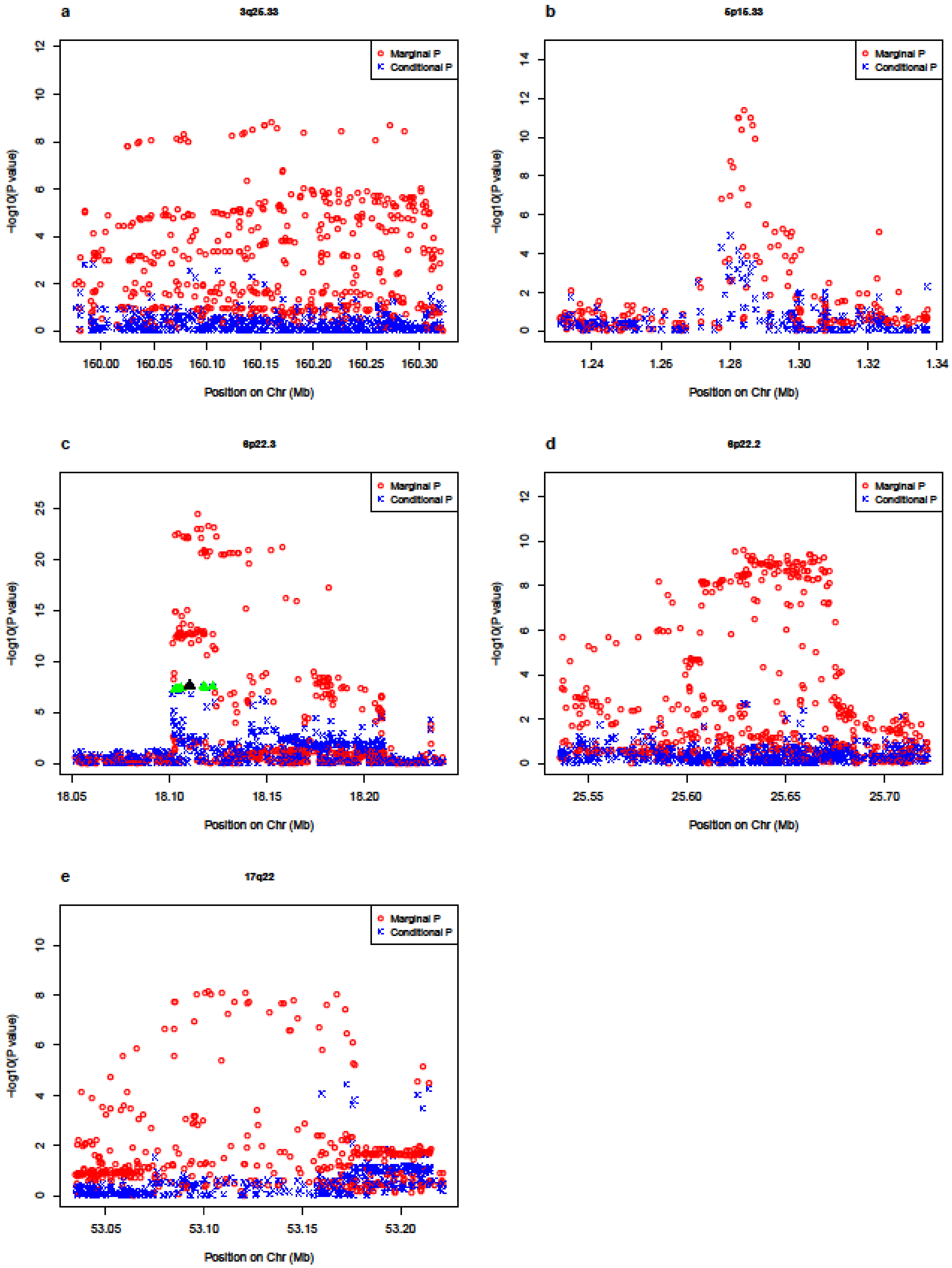

a
3q26.33
b
5p15.33
c
6p22.3
d
6p22.2
e
17q22
Marginal P
Conditional P
-log10(P value)
Position on Chr (Mb)

(f-j)

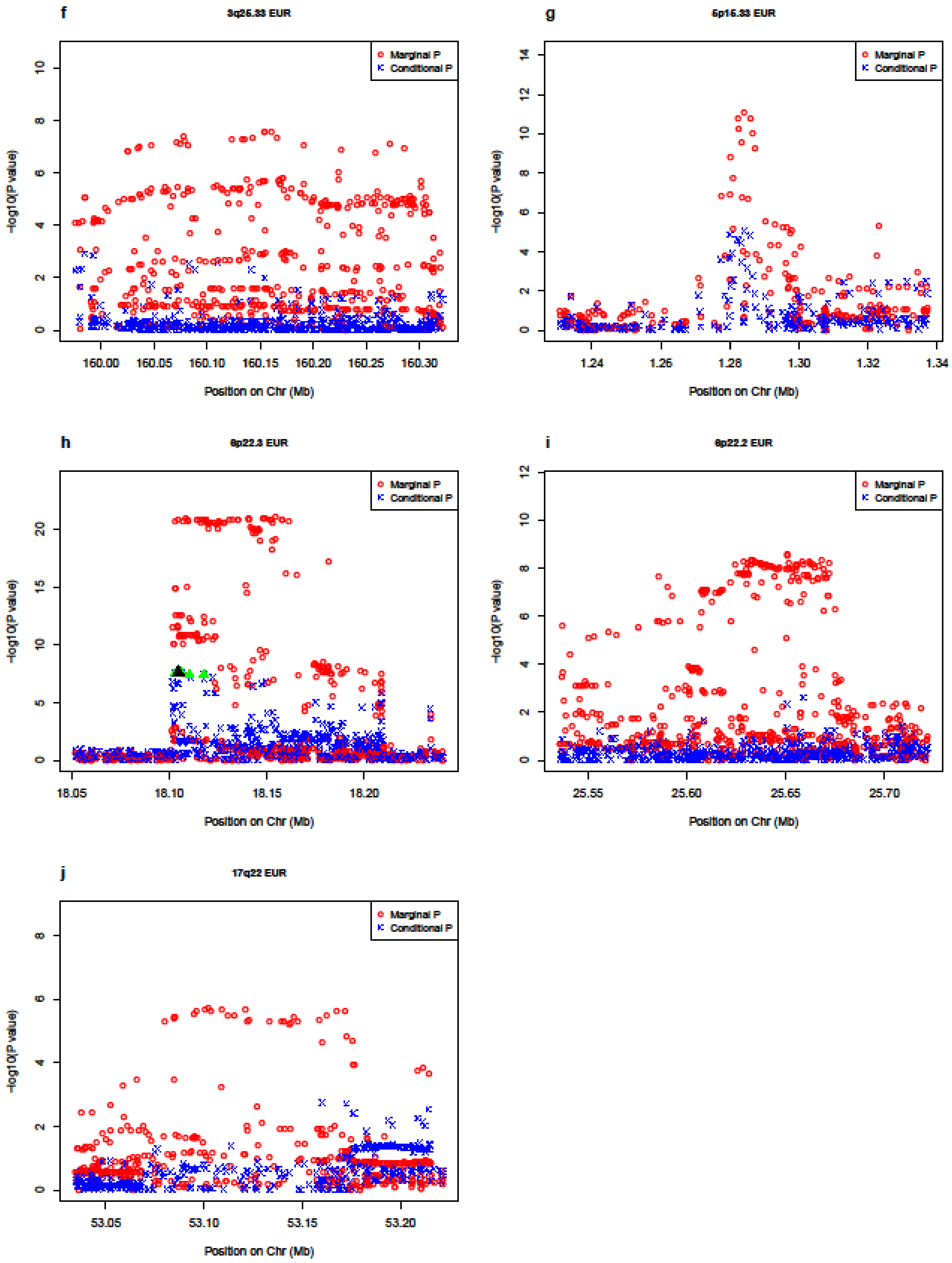
f
3q26.33 EUR
g
5p15.33 EUR
h
6p22.3 EUR
i
6p22.2 EUR
j
17q22 EUR
Marginal P
Conditional P
-log10(P value)
Position on Chr (Mb)

Supplementary Figure 4: Regional association results of susceptibility loci for EEAA

The plots display the association results surrounding the leading SNP at susceptibility locus for IEAA: (a) 4p16.3, (b) 10p11.21 and (c) 10p11.1. The colors visualize linkage disequilibrium (LD) r^2 between the leading marker and neighboring SNPs.

(a) 4p16.3

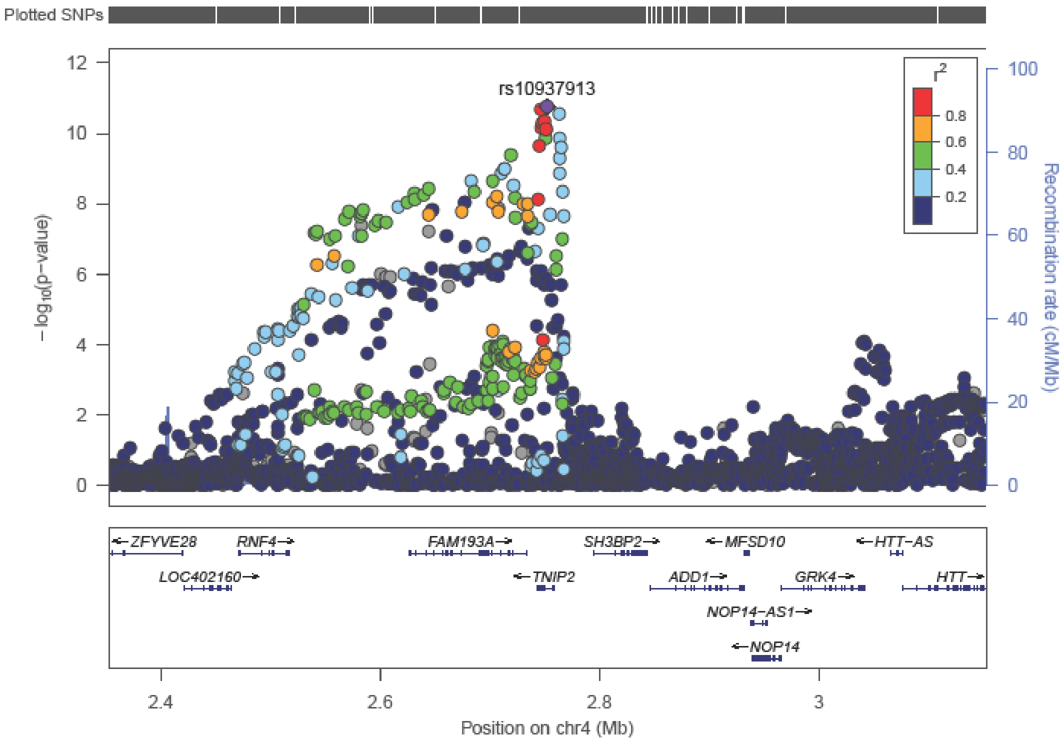

(b)10p11.21

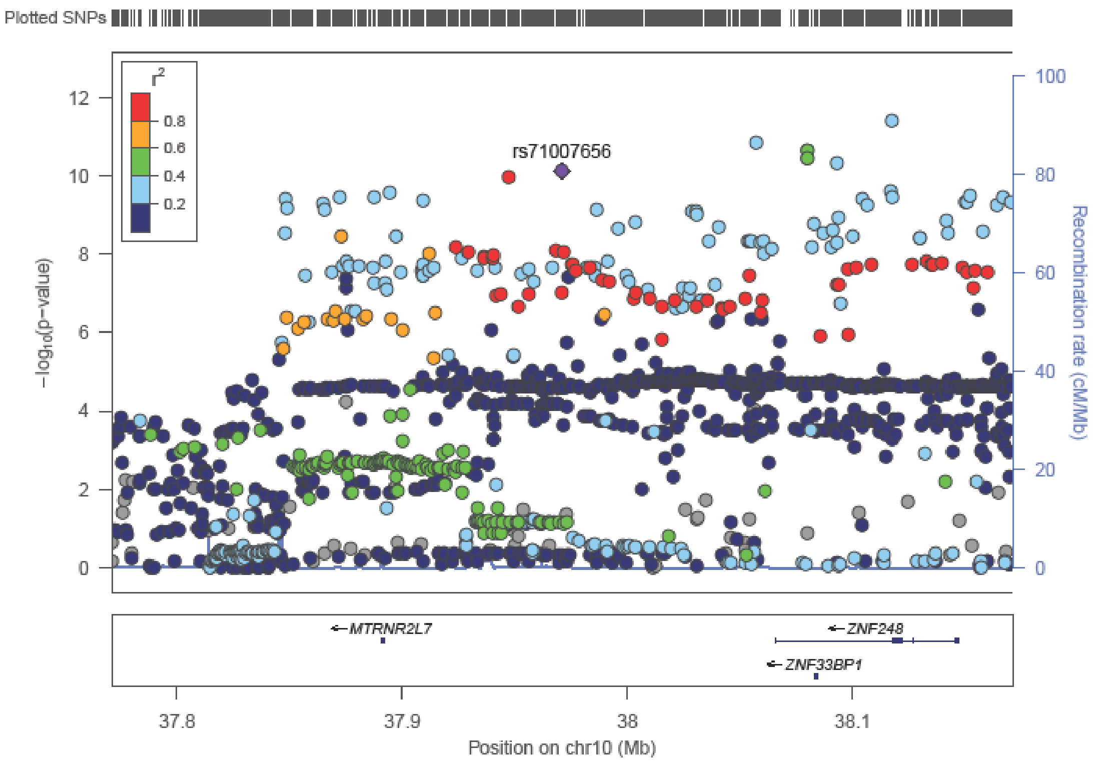

(c)10p11.1

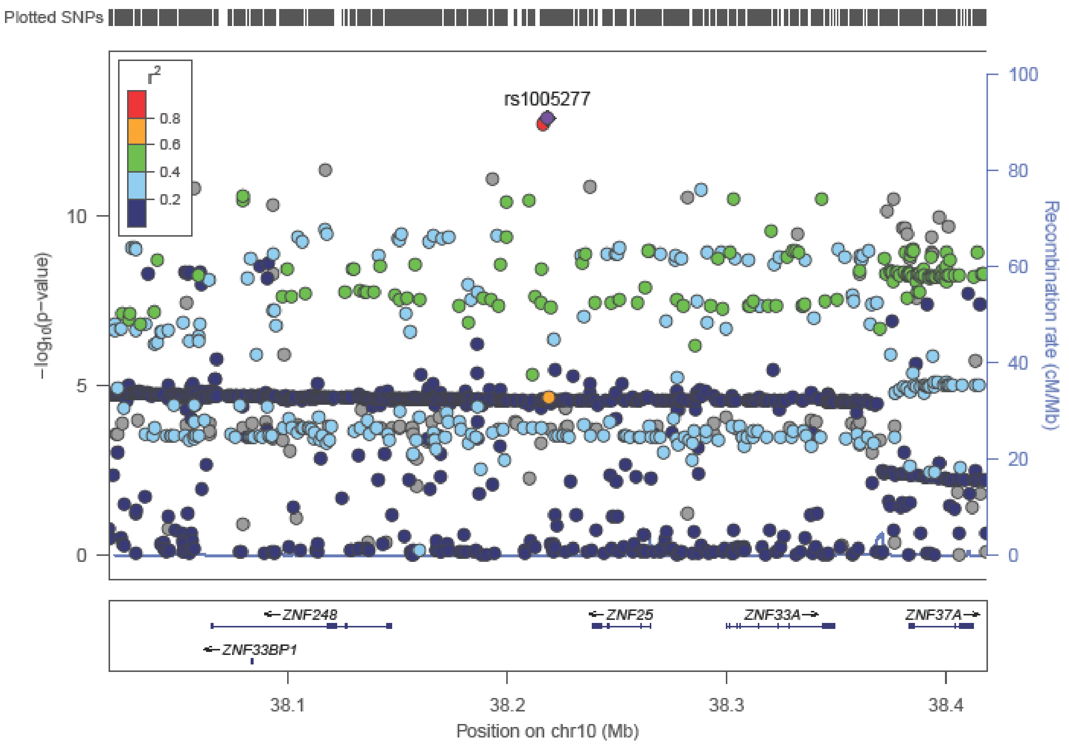

Supplementary Figure 5: GCTA conditional analysis for identifying multiple associations at EEAA susceptibility loci

The plots display the results from GCTA conditional analysis (a-c) using the GWAS summary data based on all studies. At each locus, the results present the association analysis conditional on the most significant SNPs: (a) rs10937913 in 4p16.33, (b) rs71007656 in 10p11.21, and (c) rs1005277 in 10p11. All the conditional association signals (marked in blue) are considerably lower than those in marginal associations except in the locus (b) 10p11.21, in which several SNPs remain association P values < 1.0e-07. We further examined those SNPs by conditioning on the neighboring leading SNP (rs1005277 in 10p11.1) and observed the significance of the conditional p values substantially dropped. Interestingly, the significance of the leading marker rs71007665 (in 10p11.21) substantially dropped to a *P*-value at 7.2e-03 (from a *P* value at 7.4e-11) when conditioned on rs1005277. This suggested that the association signals in the locus 10p11.21 were plausibly driven by the leading SNP rs1005377 in 10p11.1.

Similar patterns can be observed in the panels (d-f). The plots display the results from sensitivity analysis that we used the GWAS summary data based on studies 1-11 comprising individuals of EUR ancestry in the GCTA analysis.

(a-c)

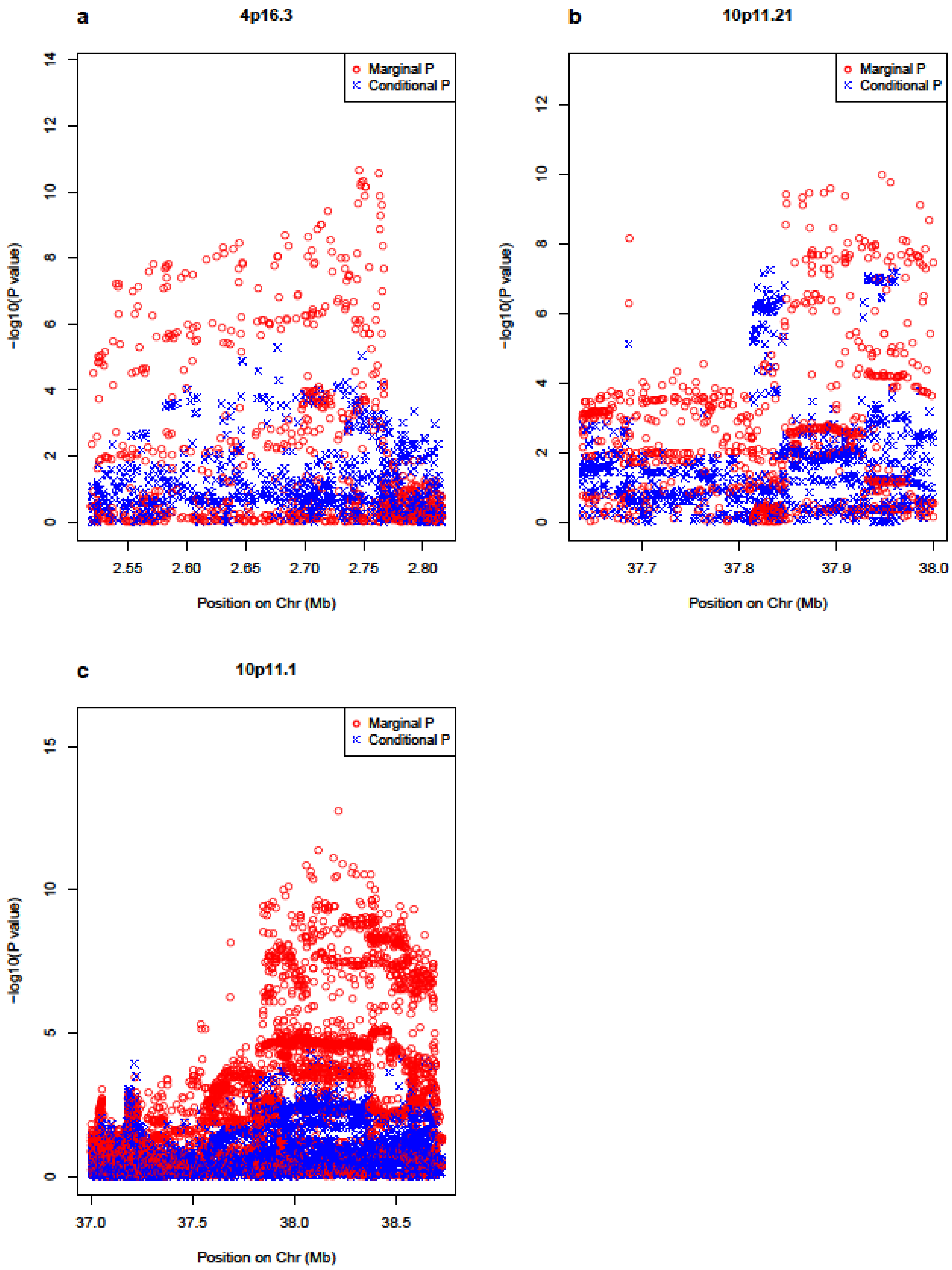
a
4p16.3
Marginal P
Conditional P
-log10(P value)
0
2
4
6
8
10
12
14
2.55
2.60
2.65
2.70
2.75
2.80
Position on Chr (Mb)
b
10p11.21
Marginal P
Conditional P
-log10(P value)
0
2
4
6
8
10
12
37.7
37.8
37.9
38.0
Position on Chr (Mb)
c
10p11.1
Marginal P
Conditional P
-log10(P value)
0
5
10
15
37.0
37.5
38.0
38.5
Position on Chr (Mb)

(d-f)

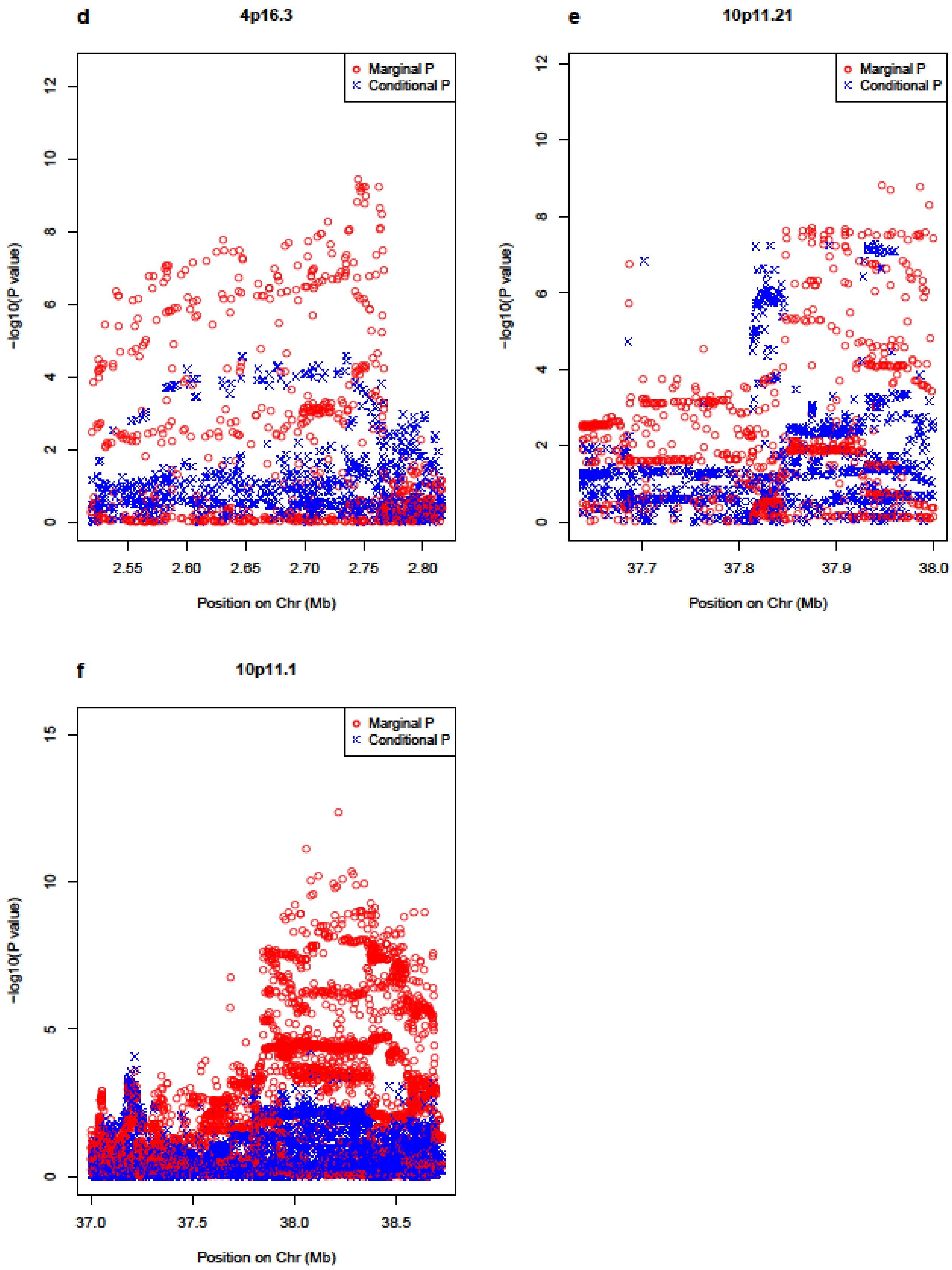

d
4p16.3
Marginal P
Conditional P
-log10(P value)
Position on Chr (Mb)
e
10p11.21
Marginal P
Conditional P
-log10(P value)
Position on Chr (Mb)
f
10p11.1
Marginal P
Conditional P
-log10(P value)
Position on Chr (Mb)

Supplementary Figure 6: Forest plots for genome-wide significant SNPs with IEAA

The forest plots present the results from fixed effects meta-analysis that combines GWAS results across all studies. The effect size is the estimate of beta slope in units of year (with respect to per minor allele) for association analysis with IEAA. The plots present the results of leading SNPs at (a) 3q25.33, (b) 5p15.33, (c-d) 6p22.3, (e) 6p22.2 and (f) 17q22, respectively.

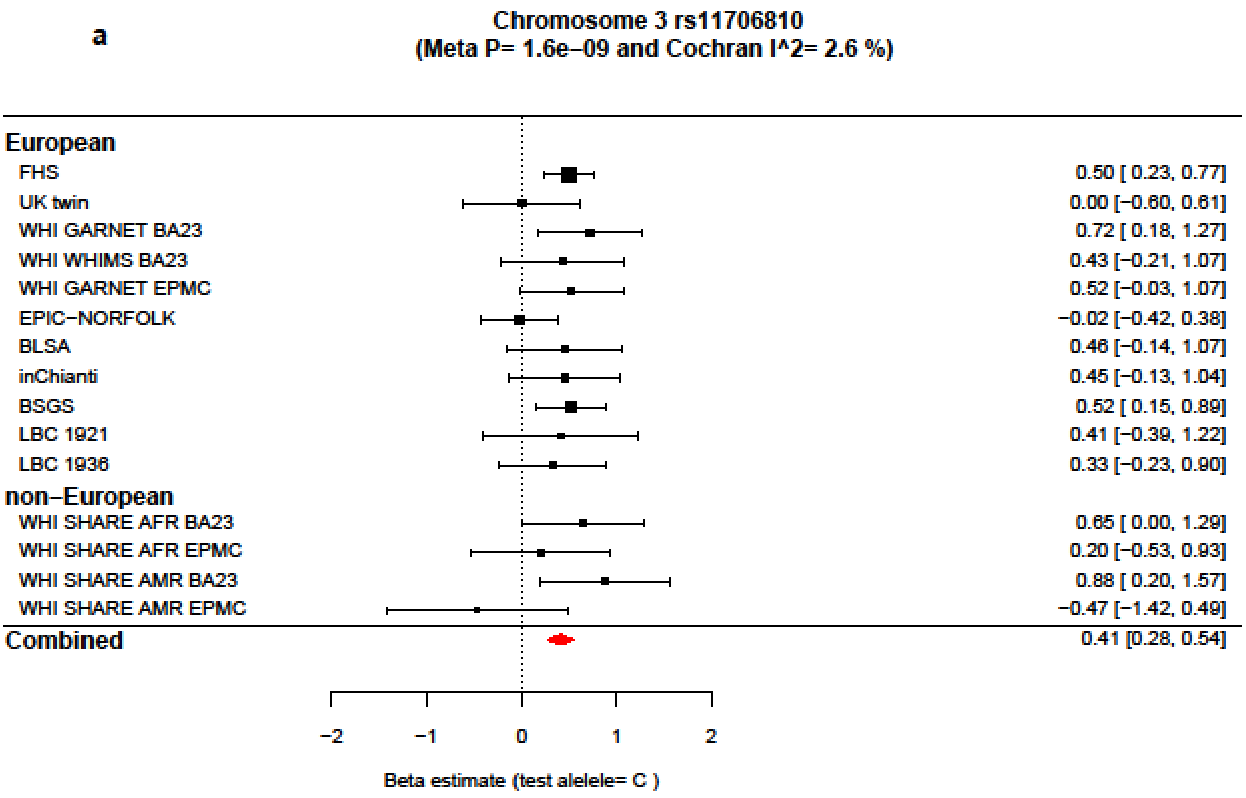
a
Chromosome 3 rs11706810
(Meta P= 1.6e−09 and Cochran I^2= 2.6 %)
European
FHS 0.50 [0.23, 0.77]
UK twin 0.00 [−0.60, 0.61]
WHI GARNET BA23 0.72 [0.18, 1.27]
WHI WHIMS BA23 0.43 [−0.21, 1.07]
WHI GARNET EPMC 0.52 [−0.03, 1.07]
EPIC−NORFOLK −0.02 [−0.42, 0.38]
BLSA 0.46 [−0.14, 1.07]
inChianti 0.45 [−0.13, 1.04]
BSGS 0.52 [0.15, 0.89]
LBC 1921 0.41 [−0.39, 1.22]
LBC 1936 0.33 [−0.23, 0.90]
non−European
WHI SHARE AFR BA23 0.65 [0.00, 1.29]
WHI SHARE AFR EPMC 0.20 [−0.53, 0.93]
WHI SHARE AMR BA23 0.88 [0.20, 1.57]
WHI SHARE AMR EPMC −0.47 [−1.42, 0.49]
Combined 0.41 [0.28, 0.54]
−2 −1 0 1 2
Beta estimate (test alelele= C)

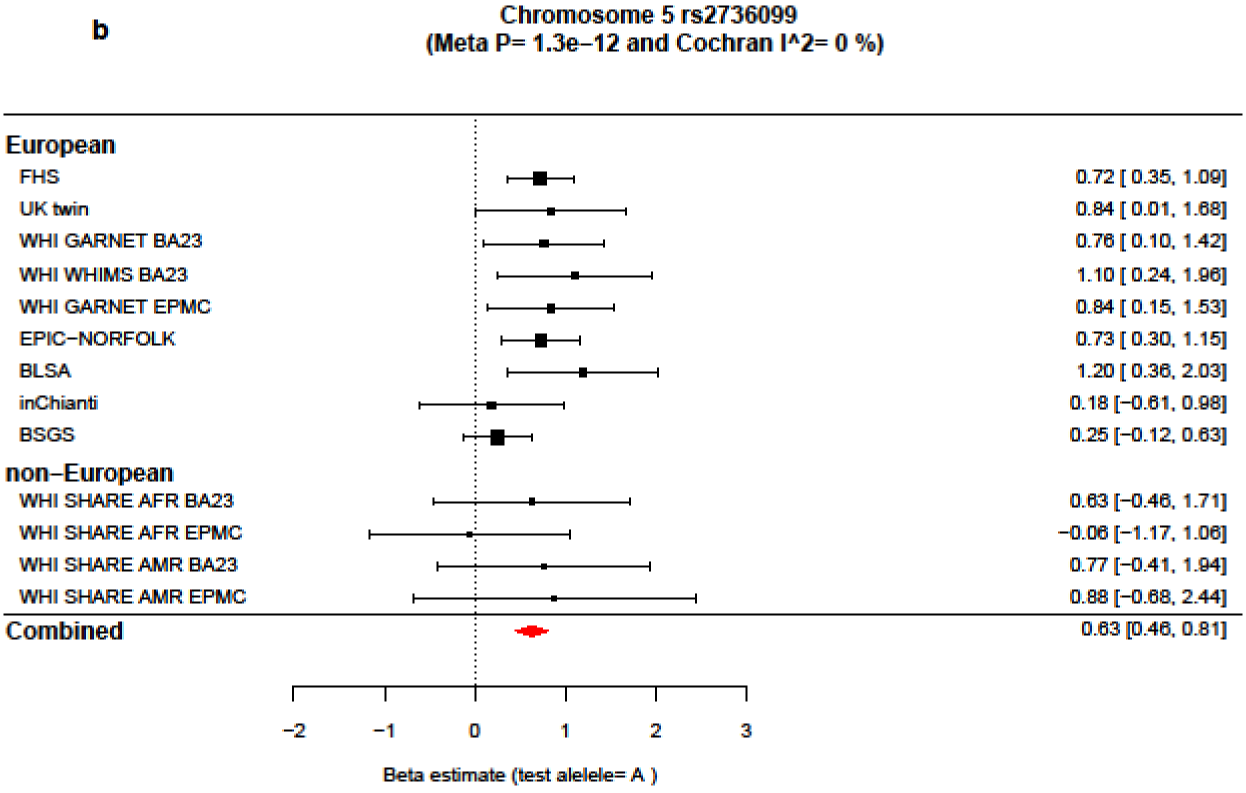
b
Chromosome 5 rs2736099
(Meta P= 1.3e−12 and Cochran I^2= 0 %)
European
FHS 0.72 [0.35, 1.09]
UK twin 0.84 [0.01, 1.68]
WHI GARNET BA23 0.76 [0.10, 1.42]
WHI WHIMS BA23 1.10 [0.24, 1.96]
WHI GARNET EPMC 0.84 [0.15, 1.53]
EPIC−NORFOLK 0.73 [0.30, 1.15]
BLSA 1.20 [0.36, 2.03]
inChianti 0.18 [−0.61, 0.98]
BSGS 0.25 [−0.12, 0.63]
non−European
WHI SHARE AFR BA23 0.63 [−0.46, 1.71]
WHI SHARE AFR EPMC −0.06 [−1.17, 1.06]
WHI SHARE AMR BA23 0.77 [−0.41, 1.94]
WHI SHARE AMR EPMC 0.88 [−0.68, 2.44]
Combined 0.63 [0.46, 0.81]
−2 −1 0 1 2 3
Beta estimate (test alelele= A)

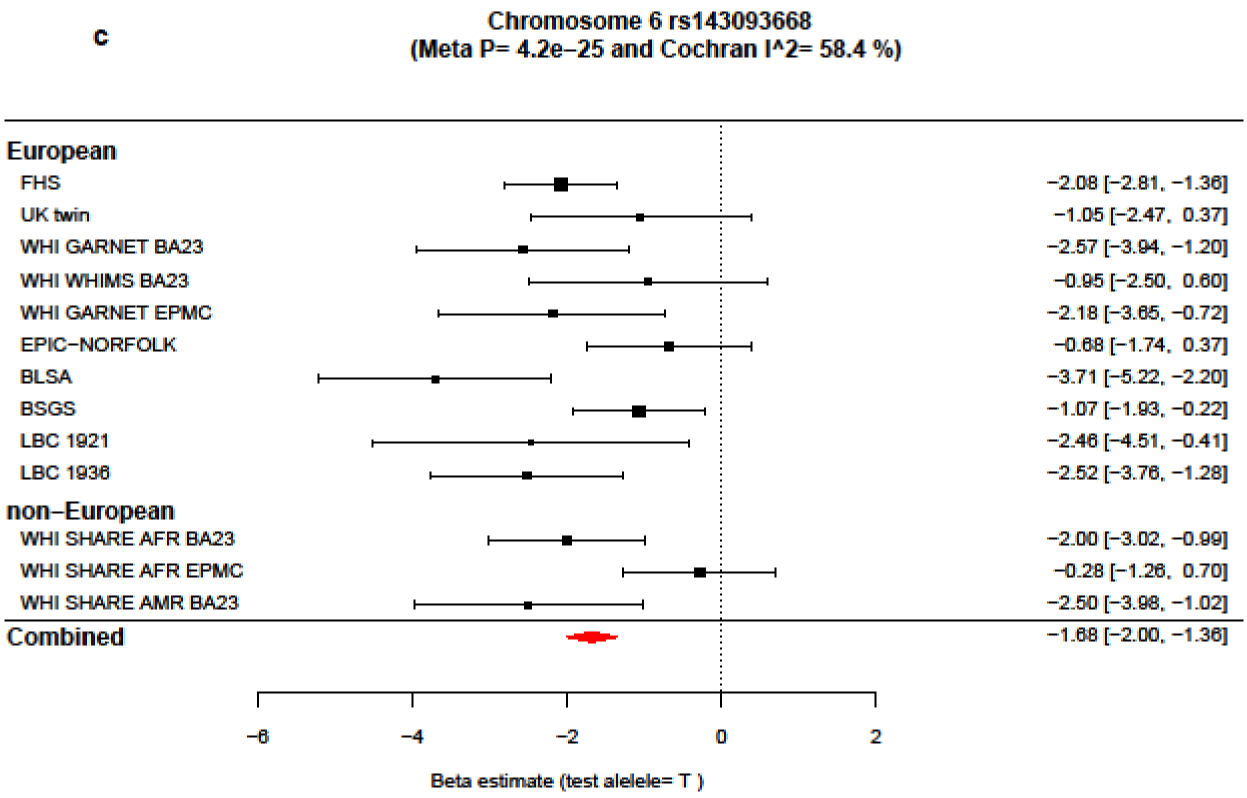
c
Chromosome 6 rs143093668
(Meta P= 4.2e−25 and Cochran I^2= 58.4 %)
European
FHS −2.08 [−2.81, −1.36]
UK twin −1.05 [−2.47, 0.37]
WHI GARNET BA23 −2.57 [−3.94, −1.20]
WHI WHIMS BA23 −0.95 [−2.50, 0.60]
WHI GARNET EPMC −2.18 [−3.65, −0.72]
EPIC−NORFOLK −0.68 [−1.74, 0.37]
BLSA −3.71 [−5.22, −2.20]
BSGS −1.07 [−1.93, −0.22]
LBC 1921 −2.46 [−4.51, −0.41]
LBC 1936 −2.52 [−3.76, −1.28]
non−European
WHI SHARE AFR BA23 −2.00 [−3.02, −0.99]
WHI SHARE AFR EPMC −0.28 [−1.26, 0.70]
WHI SHARE AMR BA23 −2.50 [−3.98, −1.02]
Combined −1.68 [−2.00, −1.36]
−6
−4
−2
0
2
Beta estimate (test alelele= T)

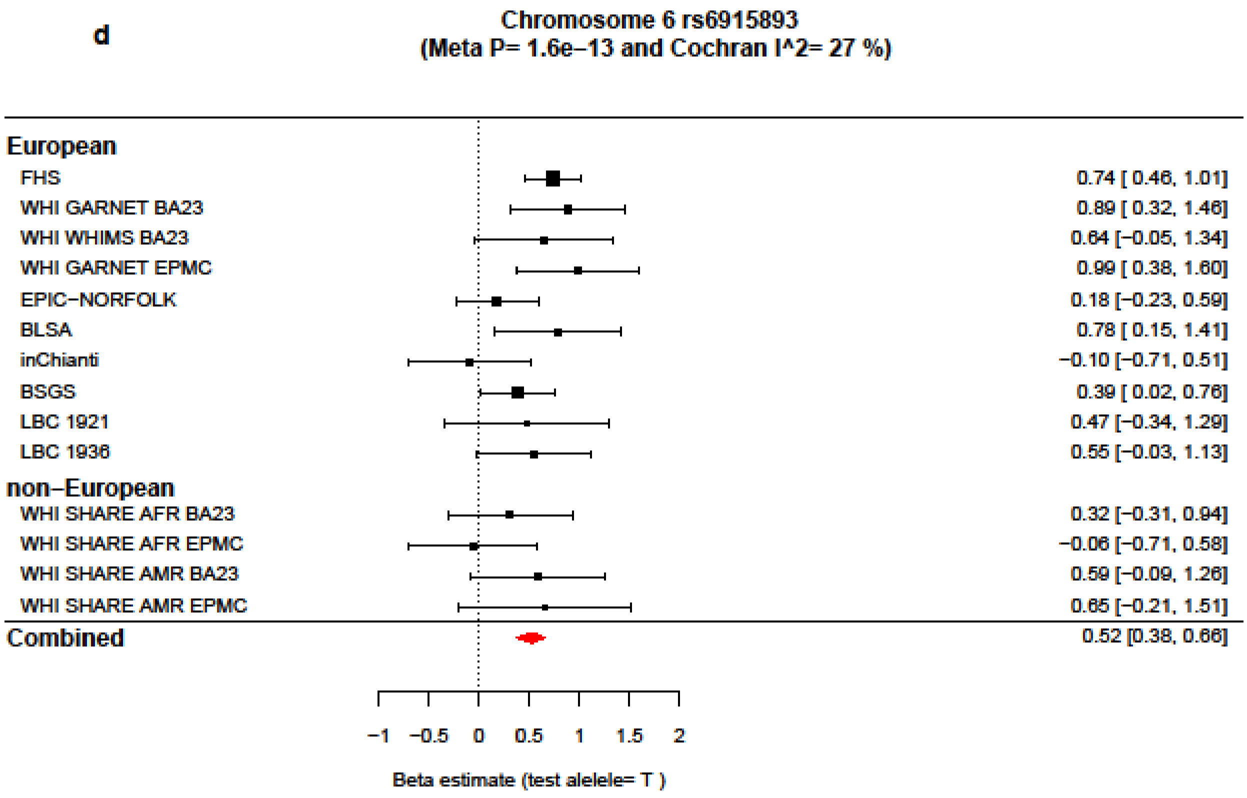
d
Chromosome 6 rs6915893
(Meta P= 1.6e−13 and Cochran I^2= 27 %)
European
FHS 0.74 [0.46, 1.01]
WHI GARNET BA23 0.89 [0.32, 1.46]
WHI WHIMS BA23 0.64 [−0.05, 1.34]
WHI GARNET EPMC 0.99 [0.38, 1.60]
EPIC−NORFOLK 0.18 [−0.23, 0.59]
BLSA 0.78 [0.15, 1.41]
inChianti −0.10 [−0.71, 0.51]
BSGS 0.39 [0.02, 0.76]
LBC 1921 0.47 [−0.34, 1.29]
LBC 1936 0.55 [−0.03, 1.13]
non−European
WHI SHARE AFR BA23 0.32 [−0.31, 0.94]
WHI SHARE AFR EPMC −0.06 [−0.71, 0.58]
WHI SHARE AMR BA23 0.59 [−0.09, 1.26]
WHI SHARE AMR EPMC 0.65 [−0.21, 1.51]
Combined 0.52 [0.38, 0.66]
−1
−0.5
0
0.5
1
1.5
2
Beta estimate (test alelele= T)

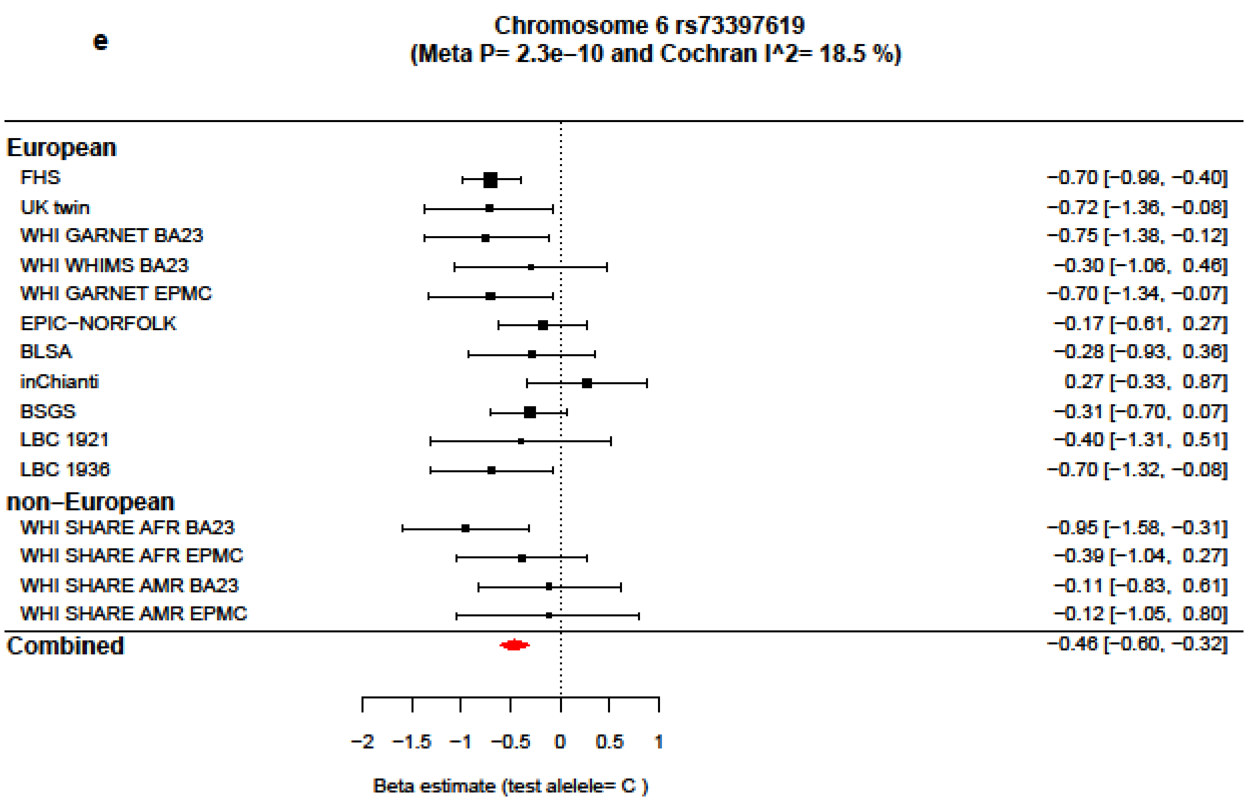
e
Chromosome 6 rs73397619
(Meta P= 2.3e−10 and Cochran I^2= 18.5 %)
European
FHS −0.70 [−0.99, −0.40]
UK twin −0.72 [−1.36, −0.08]
WHI GARNET BA23 −0.75 [−1.38, −0.12]
WHI WHIMS BA23 −0.30 [−1.06, 0.46]
WHI GARNET EPMC −0.70 [−1.34, −0.07]
EPIC−NORFOLK −0.17 [−0.61, 0.27]
BLSA −0.28 [−0.93, 0.36]
inChianti 0.27 [−0.33, 0.87]
BSGS −0.31 [−0.70, 0.07]
LBC 1921 −0.40 [−1.31, 0.51]
LBC 1936 −0.70 [−1.32, −0.08]
non−European
WHI SHARE AFR BA23 −0.95 [−1.58, −0.31]
WHI SHARE AFR EPMC −0.39 [−1.04, 0.27]
WHI SHARE AMR BA23 −0.11 [−0.83, 0.61]
WHI SHARE AMR EPMC −0.12 [−1.05, 0.80]
Combined −0.46 [−0.60, −0.32]
−2 −1.5 −1 −0.5 0 0.5 1
Beta estimate (test alelele= C)

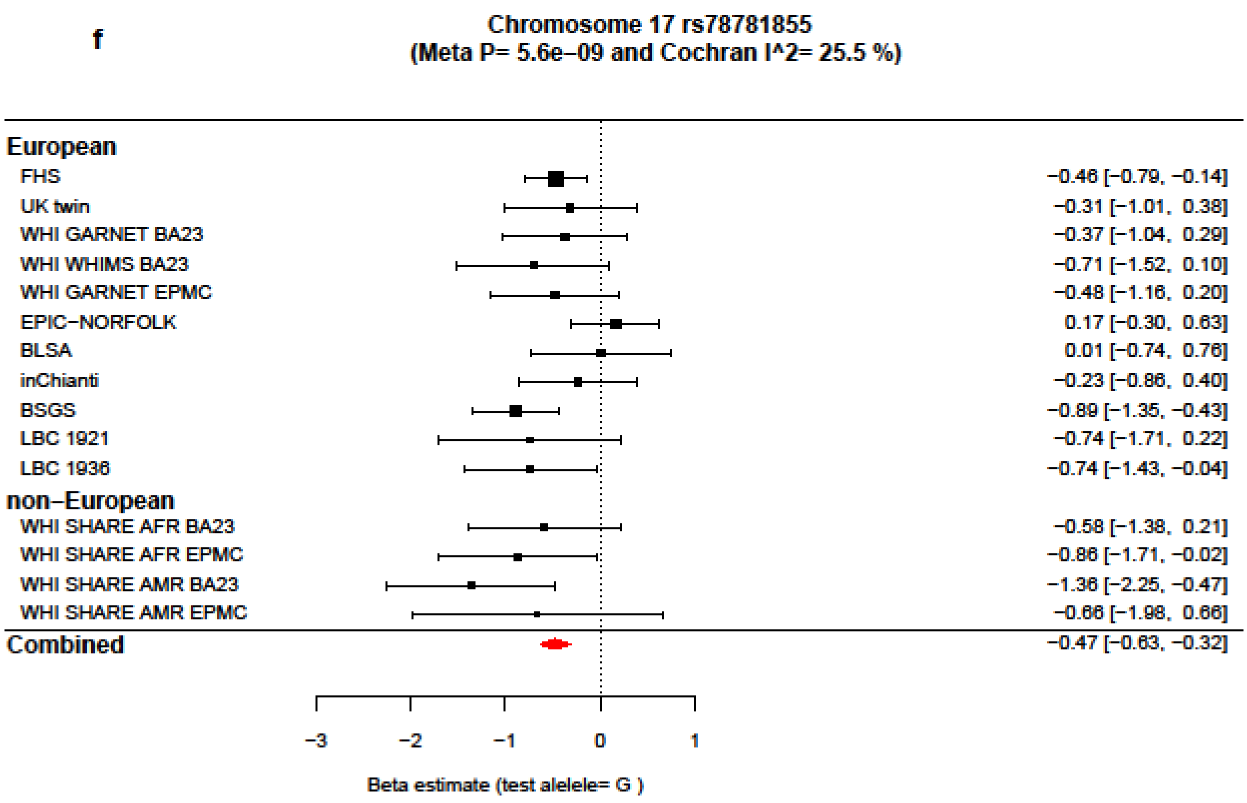
f
Chromosome 17 rs78781855
(Meta P= 5.6e−09 and Cochran I^2= 25.5 %)
European
FHS −0.46 [−0.79, −0.14]
UK twin −0.31 [−1.01, 0.38]
WHI GARNET BA23 −0.37 [−1.04, 0.29]
WHI WHIMS BA23 −0.71 [−1.52, 0.10]
WHI GARNET EPMC −0.48 [−1.16, 0.20]
EPIC−NORFOLK 0.17 [−0.30, 0.63]
BLSA 0.01 [−0.74, 0.76]
inChianti −0.23 [−0.86, 0.40]
BSGS −0.89 [−1.35, −0.43]
LBC 1921 −0.74 [−1.71, 0.22]
LBC 1936 −0.74 [−1.43, −0.04]
non−European
WHI SHARE AFR BA23 −0.58 [−1.38, 0.21]
WHI SHARE AFR EPMC −0.86 [−1.71, −0.02]
WHI SHARE AMR BA23 −1.36 [−2.25, −0.47]
WHI SHARE AMR EPMC −0.66 [−1.98, 0.66]
Combined −0.47 [−0.63, −0.32]
−3 −2 −1 0 1
Beta estimate (test alelele= G)

Supplementary Figure 7: Forest plots for genome-wide significant SNPs with EEAA

The forest plots present the results from fixed effects meta-analysis that combines GWAS results across all studies. The effect size is the estimate of beta slope in units of year (with respect to per minor allele) for association analysis with EEAA. The plots present the results of leading SNPs at (a) 4p16.3, (b) 10p11.21 and (c) 10p11.1, respectively.

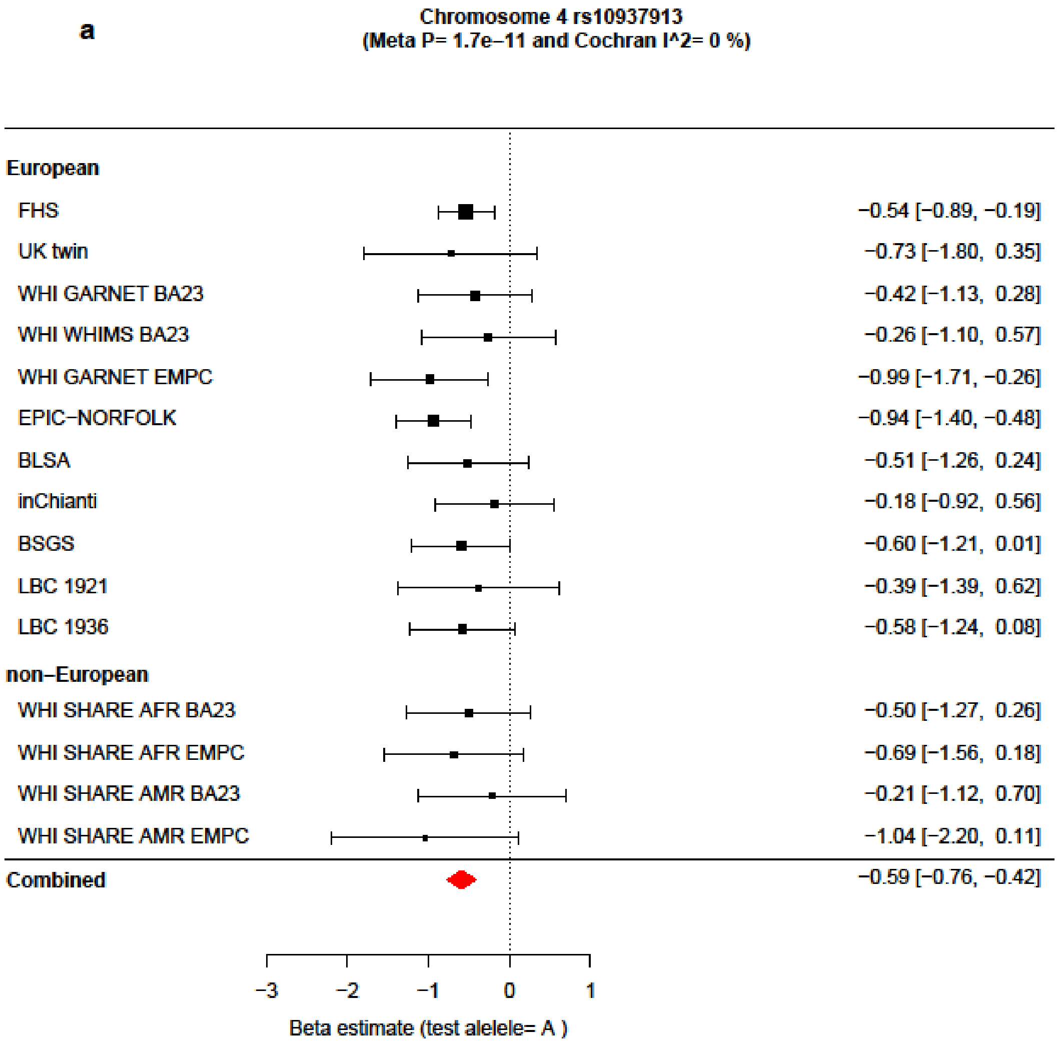

b

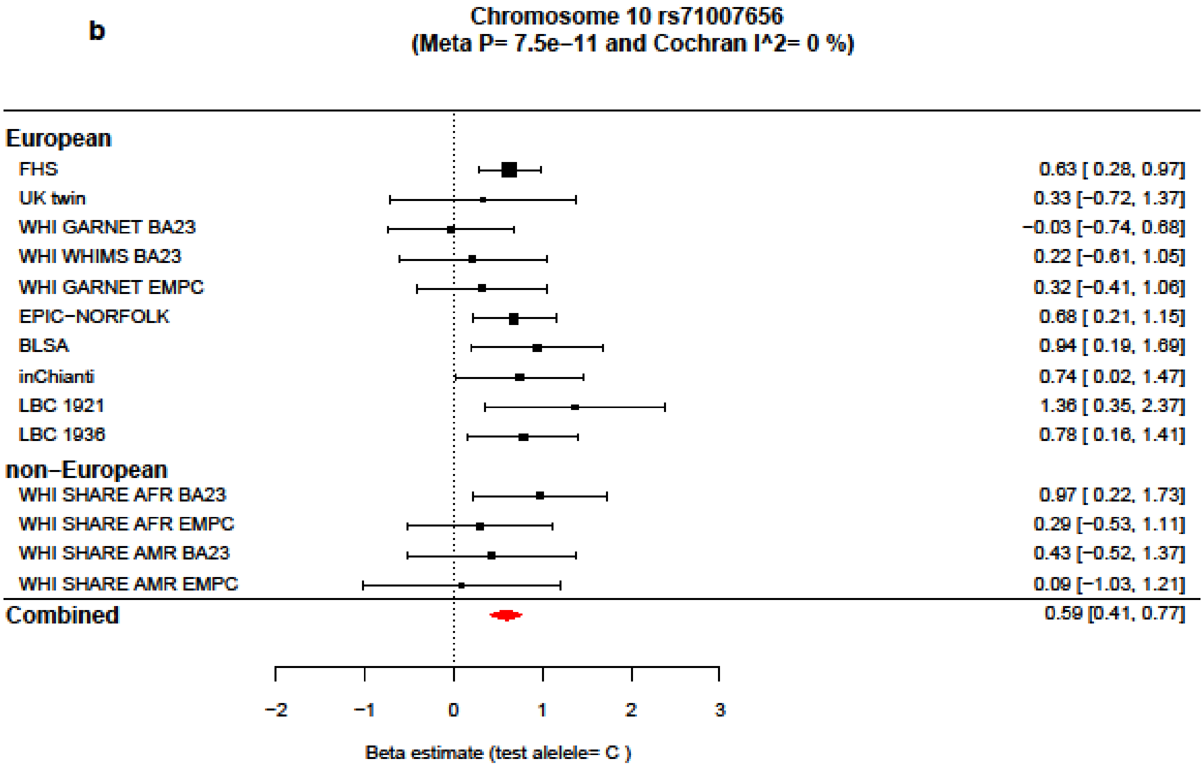

Chromosome 10 rs71007656
(Meta P= 7.5e−11 and Cochran I^2= 0 %)
European
FHS 0.63 [0.28, 0.97]
UK twin 0.33 [−0.72, 1.37]
WHI GARNET BA23 −0.03 [−0.74, 0.68]
WHI WHIMS BA23 0.22 [−0.61, 1.05]
WHI GARNET EMPC 0.32 [−0.41, 1.06]
EPIC−NORFOLK 0.68 [0.21, 1.15]
BLSA 0.94 [0.19, 1.69]
inChianti 0.74 [0.02, 1.47]
LBC 1921 1.36 [0.35, 2.37]
LBC 1936 0.78 [0.16, 1.41]
non−European
WHI SHARE AFR BA23 0.97 [0.22, 1.73]
WHI SHARE AFR EMPC 0.29 [−0.53, 1.11]
WHI SHARE AMR BA23 0.43 [−0.52, 1.37]
WHI SHARE AMR EMPC 0.09 [−1.03, 1.21]
Combined 0.59 [0.41, 0.77]
−2 −1 0 1 2 3
Beta estimate (test alelele= C)

c

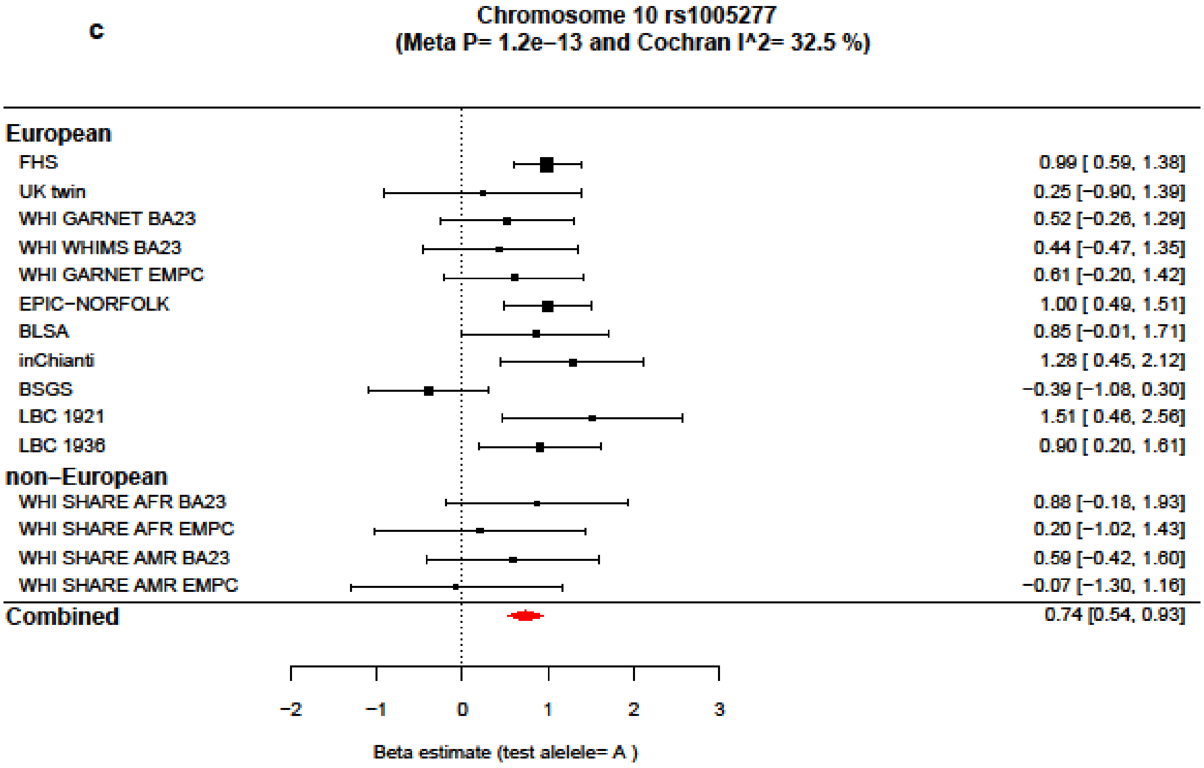

Chromosome 10 rs1005277
(Meta P= 1.2e−13 and Cochran I^2= 32.5 %)
European
FHS 0.99 [0.59, 1.38]
UK twin 0.25 [−0.90, 1.39]
WHI GARNET BA23 0.52 [−0.26, 1.29]
WHI WHIMS BA23 0.44 [−0.47, 1.35]
WHI GARNET EMPC 0.61 [−0.20, 1.42]
EPIC−NORFOLK 1.00 [0.49, 1.51]
BLSA 0.85 [−0.01, 1.71]
inChianti 1.28 [0.45, 2.12]
BSGS −0.39 [−1.08, 0.30]
LBC 1921 1.51 [0.46, 2.56]
LBC 1936 0.90 [0.20, 1.61]
non−European
WHI SHARE AFR BA23 0.88 [−0.18, 1.93]
WHI SHARE AFR EMPC 0.20 [−1.02, 1.43]
WHI SHARE AMR BA23 0.59 [−0.42, 1.60]
WHI SHARE AMR EMPC −0.07 [−1.30, 1.16]
Combined 0.74 [0.54, 0.93]
−2 −1 0 1 2 3
Beta estimate (test alelele= A)

Supplementary Figure 8: Roadmap Epigenomics chromatin state analysis

The plot summarizes the chromatin states for a study SNP across 127 diverse human cell/tissue types from primary hidden Markov model (HMM) analysis[1]. The HMM classifies DNA regions dynamically into one of the 15 states as described below.

- State 1 - Red - TssA (Active_TSS)
- State 2 - OrangeRed - TssAFlnk (Flanking_Active_TSS)
- State 3 - LimeGreen - TxFlnk (Transcr_at_gene_5_and_3primer)
- State 4 - Green - Tx (Strong_transcription)
- State 5 - DarkGreen - TxWk (Weak_transcription)
- State 6 - GreenYellow - EnhG (Genic_enhancers)
- State 7 - Yellow - Enh (Enhancers)
- State 8 - MediumAquamarine - ZNF/Rpts (ZNF_genes&repeats)
- State 9 - PaleTurquoise - Het (Heterochromatin)
- State 10 - IndianRed - TssBiv (Bivalent/Poised_TSS)
- State 11 - DarkSalmon - BivFlnk (Flanking_Bivalent_TSS/Enh)
- State 12 - DarkKhaki - EnhBiv (Bivalent_Enhancer)
- State 13 - Silver - ReprPC (Repressed_PolyComb)
- State 14 - Gainsboro - ReprPCWk (Weak_Repressed_PolyComb)
- State 15 - White - Quies (Quiescent/Low)

More details can be found in https://genome.ucsc.edu/cgi-bin/hgTrackUi?hgsid=433891379_Ri32gCKnmDLSQiE1ZgjJAgfHAo6H&c=chr16&g=hub_24125_RoadmapConsolidatedAssaya27004

a: Hidden Markov Model analysis for rs11706810 in chromosome 3 KPNA4

The lead SNP rs11706810 associated with IEAA is located in a chromosomal region transcribed in more than 100 cell lines according to the chromatin state analysis.

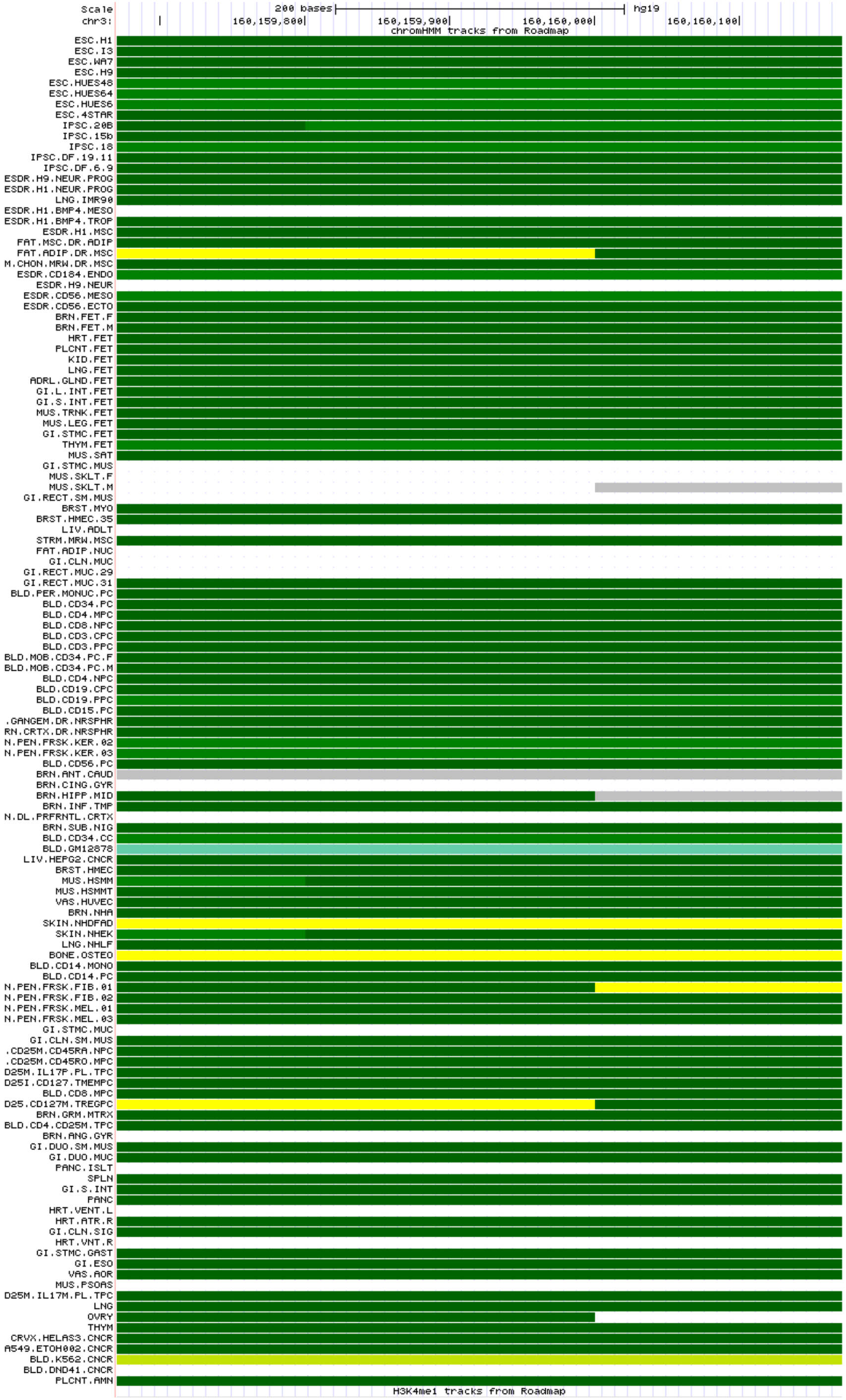

b: Hidden Markov Model analysis for rs2736099 in chromosome 5 TERT

The IEAA-associated SNP rs2736099 is located in chromosomal region that is known to be transcribed in human embryonic stem cells, induced pluripotent stem cells and hematopoietic stem cells

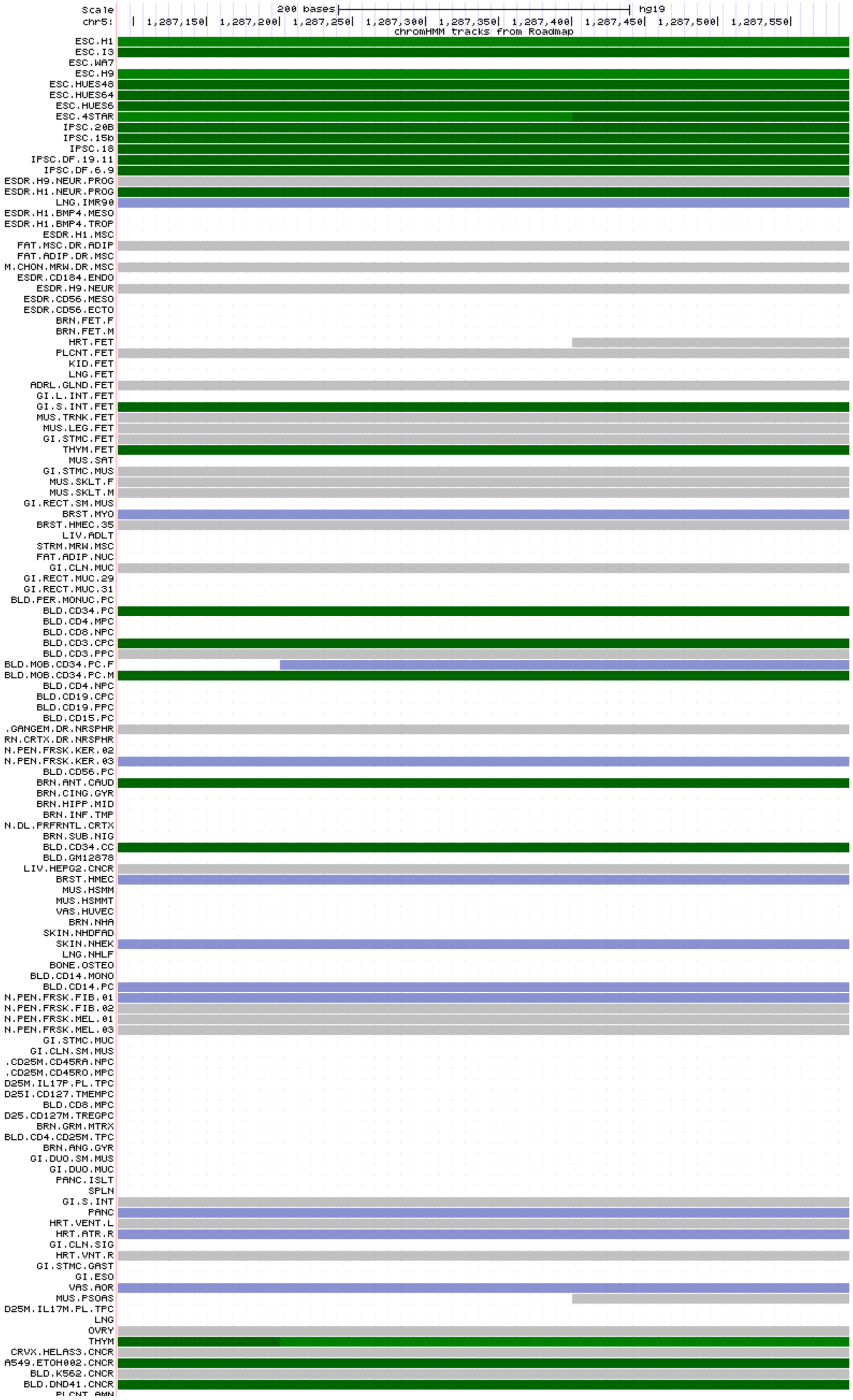

c: Hidden Markov Model analysis for rs143093668 in chromosome 6 KIF13A-NHLFC1

The plot below shows that the IEAA-associated SNP rs143093668 is located in a chromosomal region that (1) is weakly repressed by Polycomb proteins in several blood cell lines and (2) is transcribed in several breast or muscle cell lines

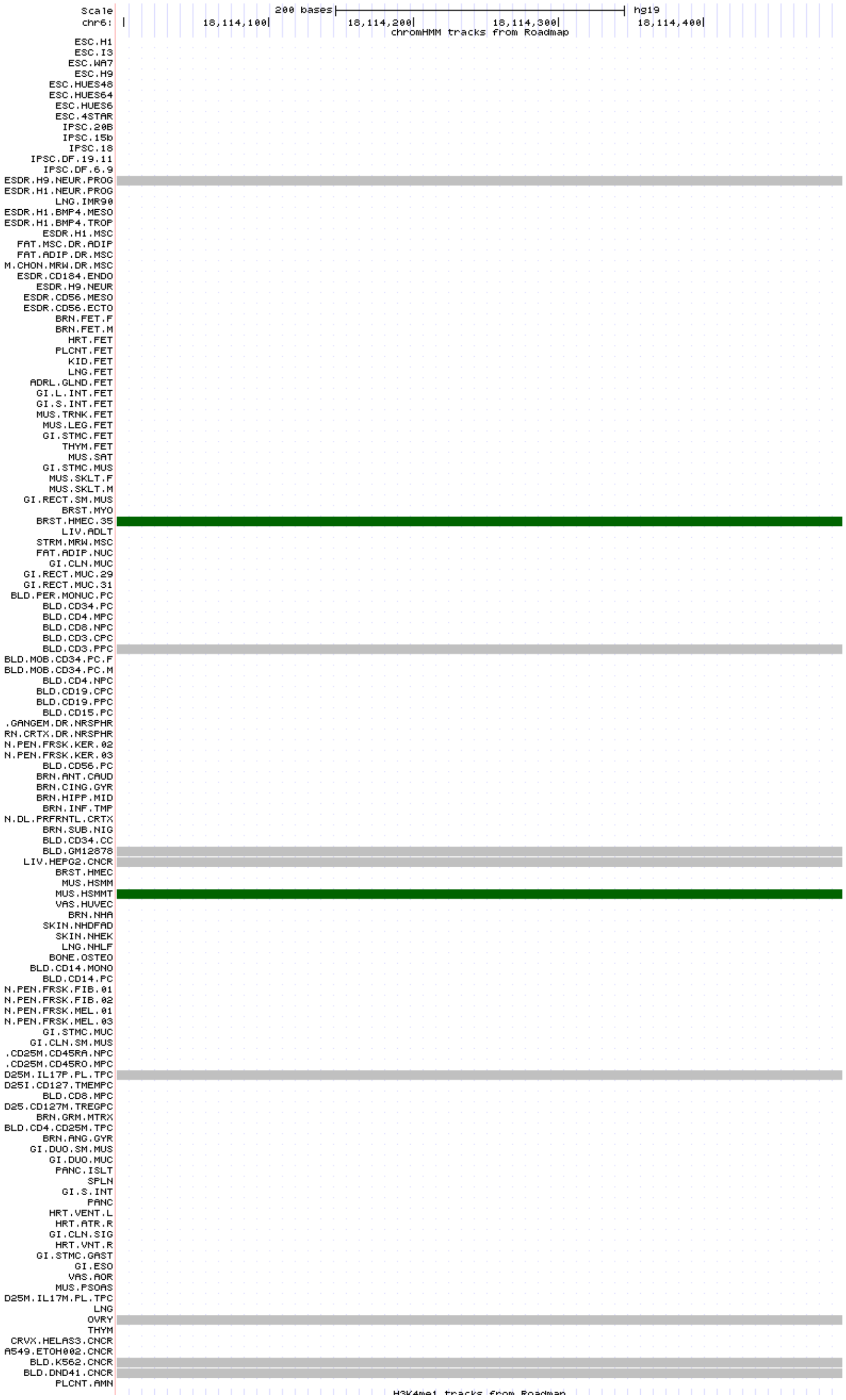
Scale
chr6:
200 bases
hg19
18,114,100
18,114,200
18,114,300
18,114,400
chromHMM tracks from Roadmap
ESC.H1
ESC.I3
ESC.WA7
ESC.H9
ESC.HUES48
ESC.HUES64
ESC.HUES6
ESC.4STAR
IPSC.20B
IPSC.15b
IPSC.18
IPSC.DF.19.11
IPSC.DF.6.9
ESDR.H9.NEUR.PROG
ESDR.H1.NEUR.PROG
LNG.IMR90
ESDR.H1.BMP4.MESO
ESDR.H1.BMP4.TROP
ESDR.H1.MSC
FAT.MSC.DR.ADIP
FAT.ADIP.DR.MSC
M.CHON.MRW.DR.MSC
ESDR.CD184.ENDO
ESDR.H9.NEUR
ESDR.CD56.MESO
ESDR.CD56.ECTO
BRN.FET.F
BRN.FET.M
HRT.FET
PLCNT.FET
KID.FET
LNG.FET
ADRL.GLND.FET
GI.L.INT.FET
GI.S.INT.FET
MUS.TRNK.FET
MUS.LEG.FET
GI.STMC.FET
THYM.FET
MUS.SAT
GI.STMC.MUS
MUS.SKLT.F
MUS.SKLT.M
GI.RECT.SM.MUS
BRST.MYO
BRST.HMEC.35
LIV.ADLT
STRM.MRW.MSC
FAT.ADIP.NUC
GI.CLN.MUC
GI.RECT.MUC.29
GI.RECT.MUC.31
BLD.PER.MONUC.PC
BLD.CD34.PC
BLD.CD4.MPC
BLD.CD8.NPC
BLD.CD3.CPC
BLD.CD3.PPC
BLD.MOB.CD34.PC.F
BLD.MOB.CD34.PC.M
BLD.CD4.NPC
BLD.CD19.CPC
BLD.CD19.PPC
BLD.CD15.PC
.GANGEM.DR.NRSPHR
RN.CRTX.DR.NRSPHR
N.PEN.FRSK.KER.02
N.PEN.FRSK.KER.03
BLD.CD56.PC
BRN.ANT.CAUD
BRN.CING.GYR
BRN.HIPP.MID
BRN.INF.TMP
N.DL.PRFRNTL.CRTX
BRN.SUB.NIG
BLD.CD34.CC
BLD.GM12878
LIV.HEPG2.CNCR
BRST.HMEC
MUS.HSMM
MUS.HSMMT
VAS.HUVEC
BRN.NHA
SKIN.NHDFAD
SKIN.NHEK
LNG.NHLF
BONE.OSTEO
BLD.CD14.MONO
BLD.CD14.PC
N.PEN.FRSK.FIB.01
N.PEN.FRSK.FIB.02
N.PEN.FRSK.MEL.01
N.PEN.FRSK.MEL.03
GI.STMC.MUC
GI.CLN.SM.MUS
.CD25M.CD45RA.NPC
.CD25M.CD45RO.MPC
D25M.IL17P.PL.TPC
D25I.CD127.TMEMPC
BLD.CD8.MPC
D25.CD127M.TREGPC
BRN.GRM.MTRX
BLD.CD4.CD25M.TPC
BRN.ANG.GYR
GI.DUO.SM.MUS
GI.DUO.MUC
PANC.ISLT
SPLN
GI.S.INT
PANC
HRT.VENT.L
HRT.ATR.R
GI.CLN.SIG
HRT.VNT.R
GI.STMC.GAST
GI.ESO
VAS.AOR
MUS.PSOAS
D25M.IL17M.PL.TPC
LNG
OVRY
THYM
CRVX.HELAS3.CNCR
A549.ETOH002.CNCR
BLD.K562.CNCR
BLD.DND41.CNCR
PLCNT.AMN
H3K4me1 tracks from Roadmap

d: Hidden Markov Model analysis for rs6915893 in chromosome 6 KIF13A-NHLFC1

Similar with the SNP rs143093668 , the second leading variant rs6915893 in 6p22.3 is located in a chromosomal region that is (1) weakly repressed by Polycomb proteins in several blood cell lines and (2) is transcribed in several breast or muscle cell lines

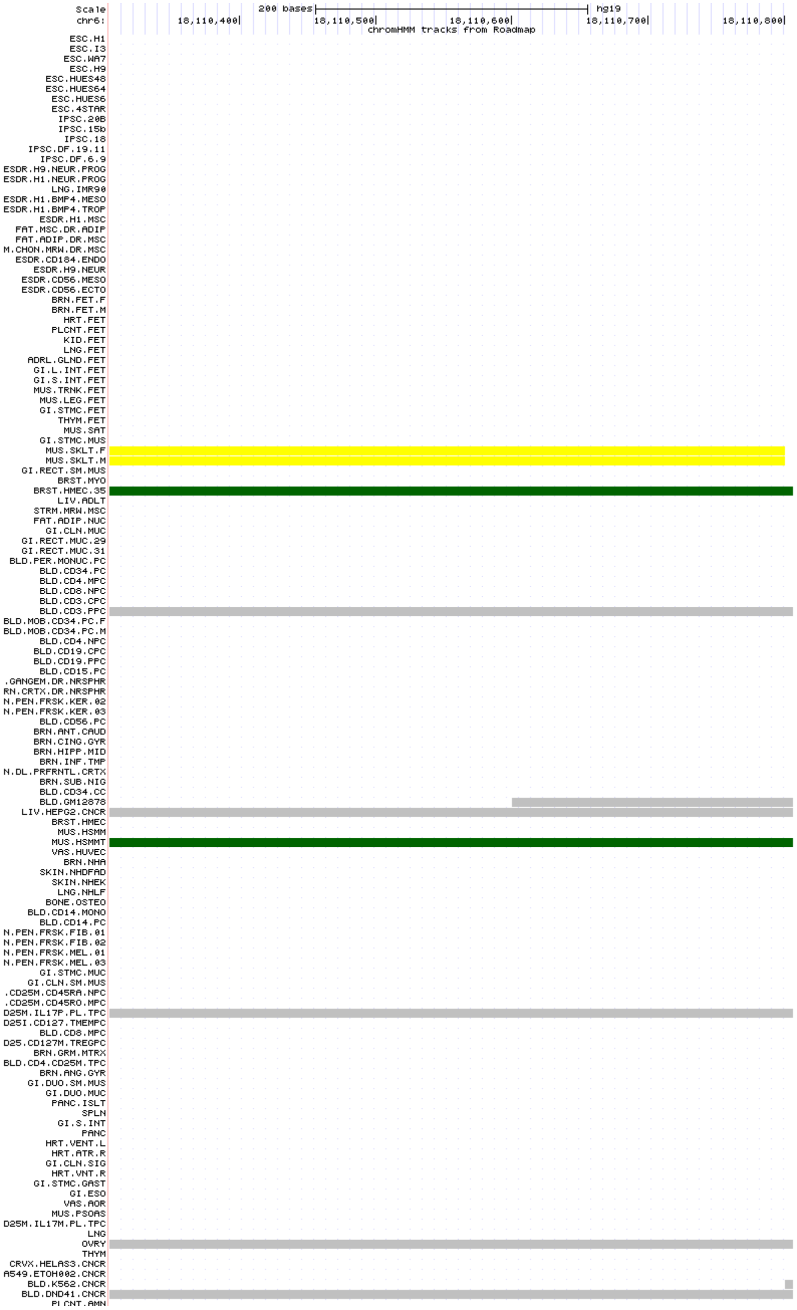

e: Hidden Markov Model analysis for rs73397610 in chromosome 6 LRRC16A-SCGN

The figure below shows that the leading SNP rs73397619 is located in a chromosomal region actively transcribed in several stem cell lines.

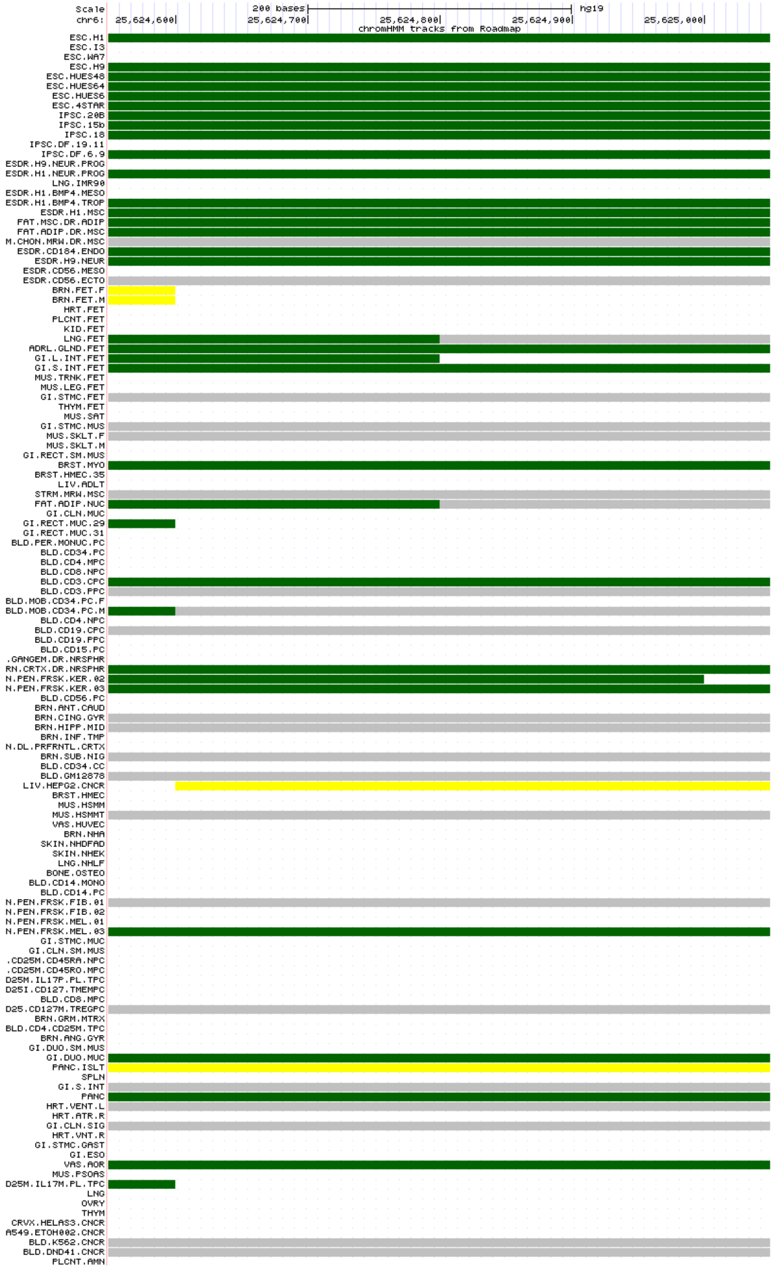

f: Hidden Markov Model analysis for rs78781855 in chromosome 17

In 17q22, the leading variant rs78781855 associated with IEAA is located in a chromosomal region that is known to be transcribed in more than 40% of cell lines.

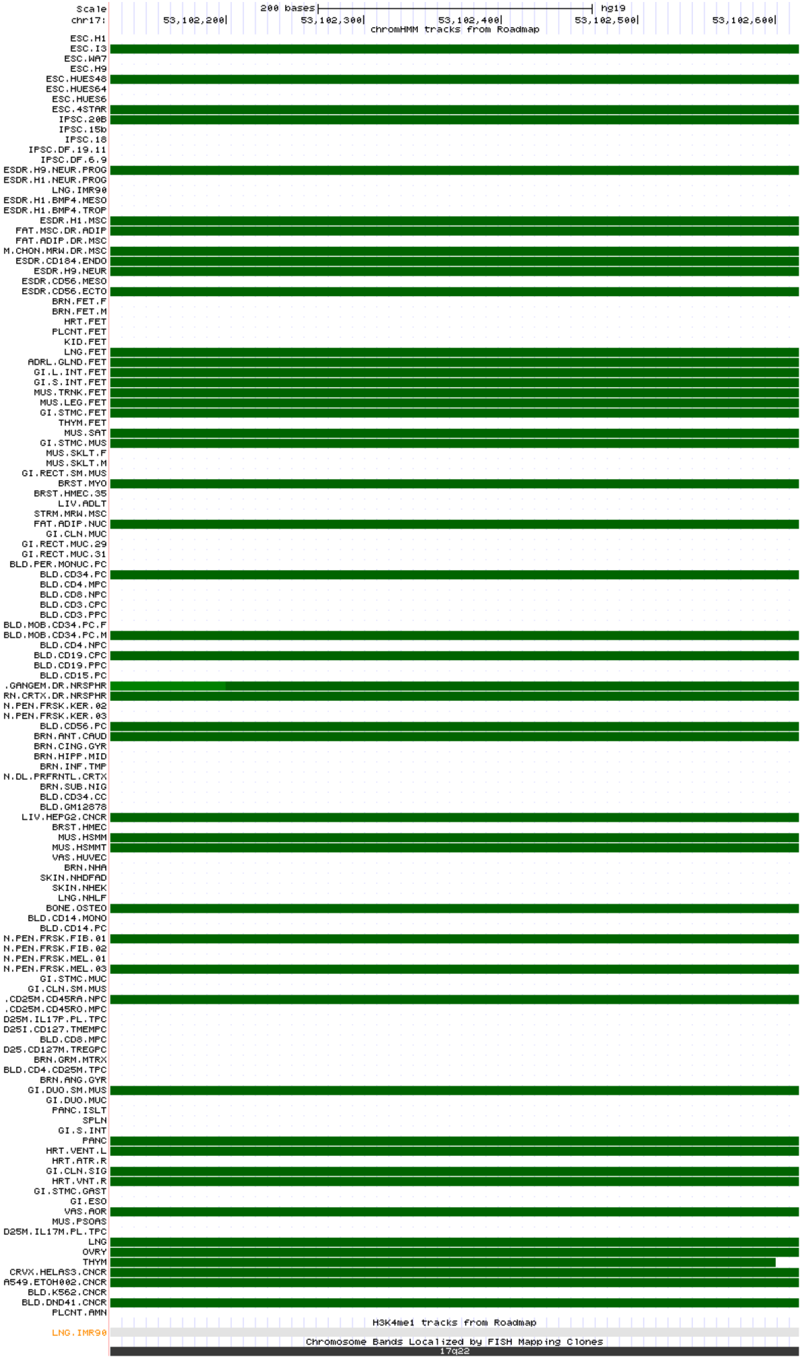

g: Hidden Markov Model analysis for rs10937913 in chromosome 4

The HMM analysis shows that the EEAA-associated SNP rs10937913 is located in an open chromosomal region that is known to be actively transcribed in almost all cell lines (> 99%).

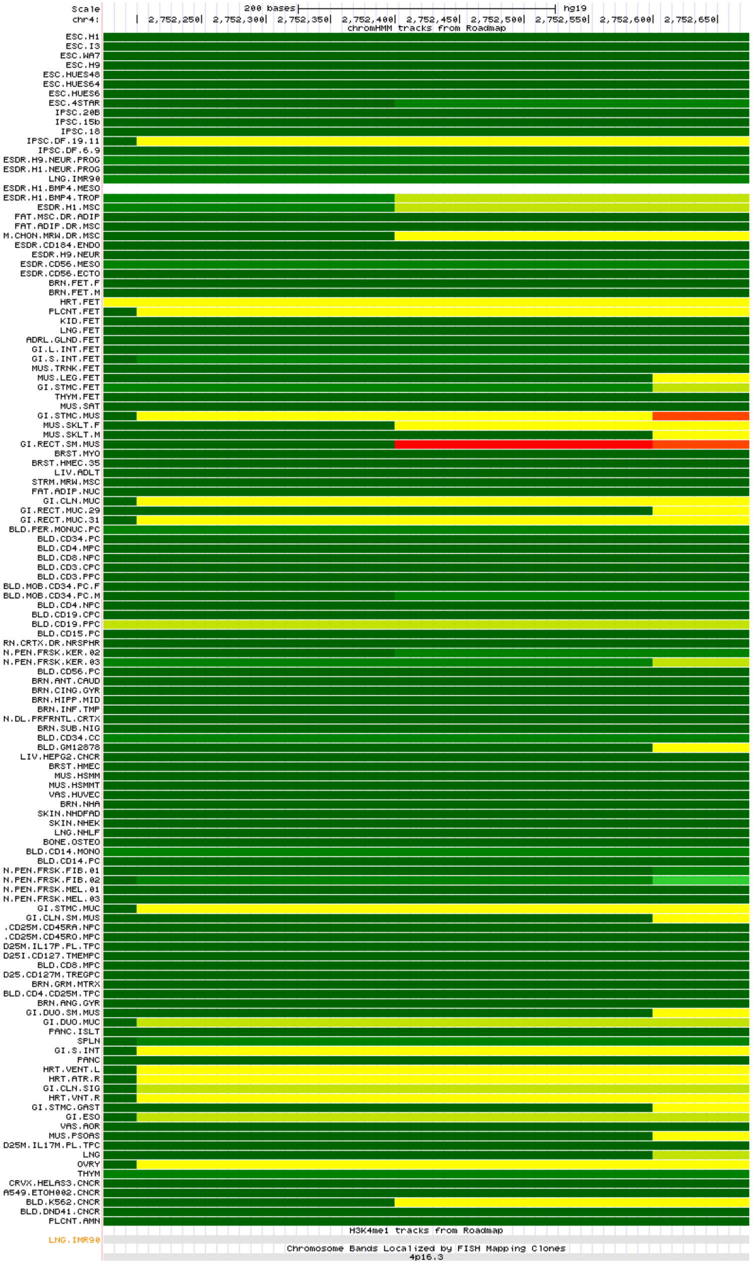

h: Hidden Markov Model analysis for rs71007656 in chromosome 10

The chromatin state analysis shows that the INDEL marker rs71007656 is located in a heterochromatin region for most of cell lines.

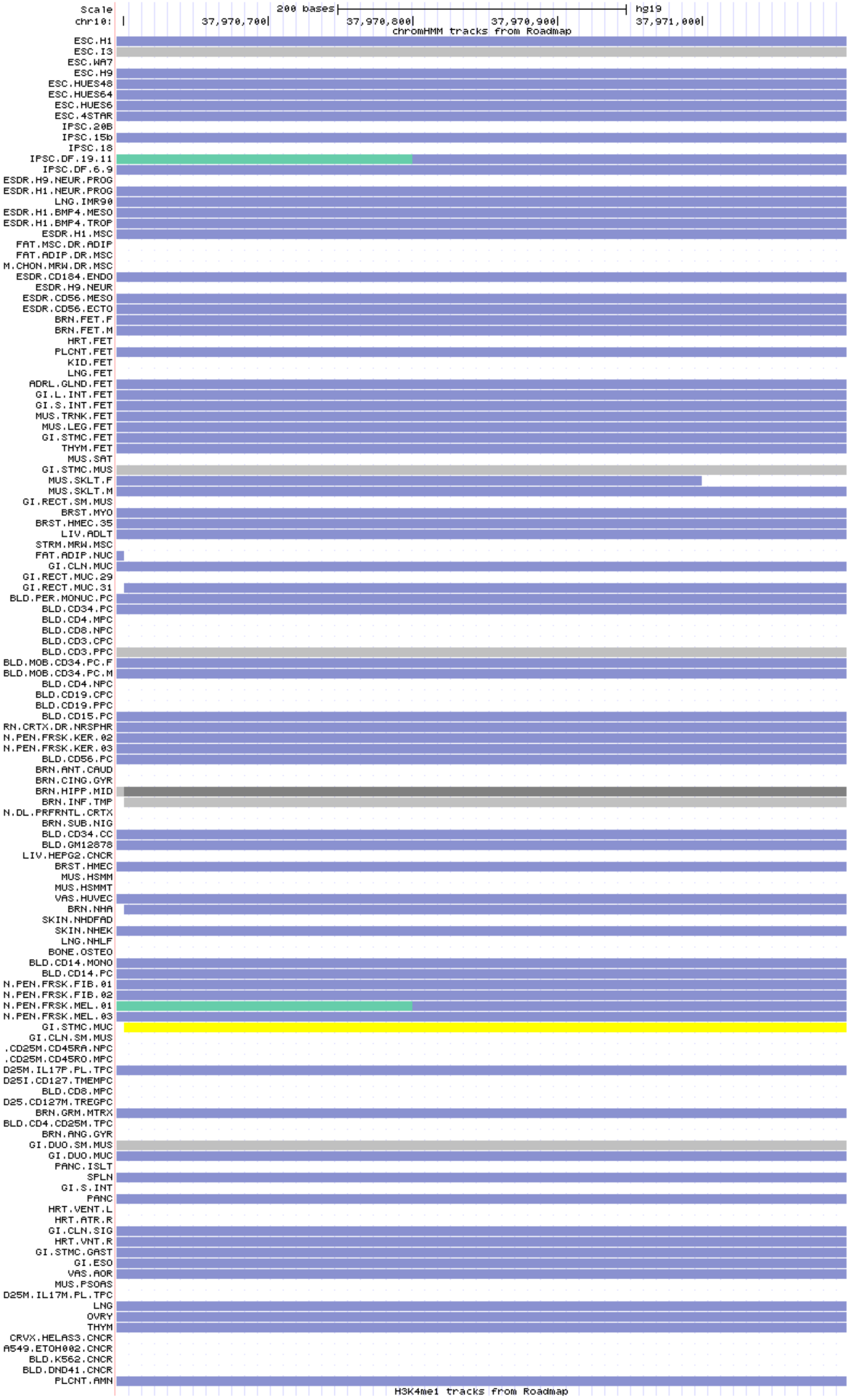

i: Hidden Markov Model analysis for rs1005277 in chromosome 10

The EEAA-associated leading SNP rs1005277 is located in a chromosomal region actively transcribed in a few cell lines or in a chromosomal region at heterochromatin state for several cell lines including human embryonic stem cells.

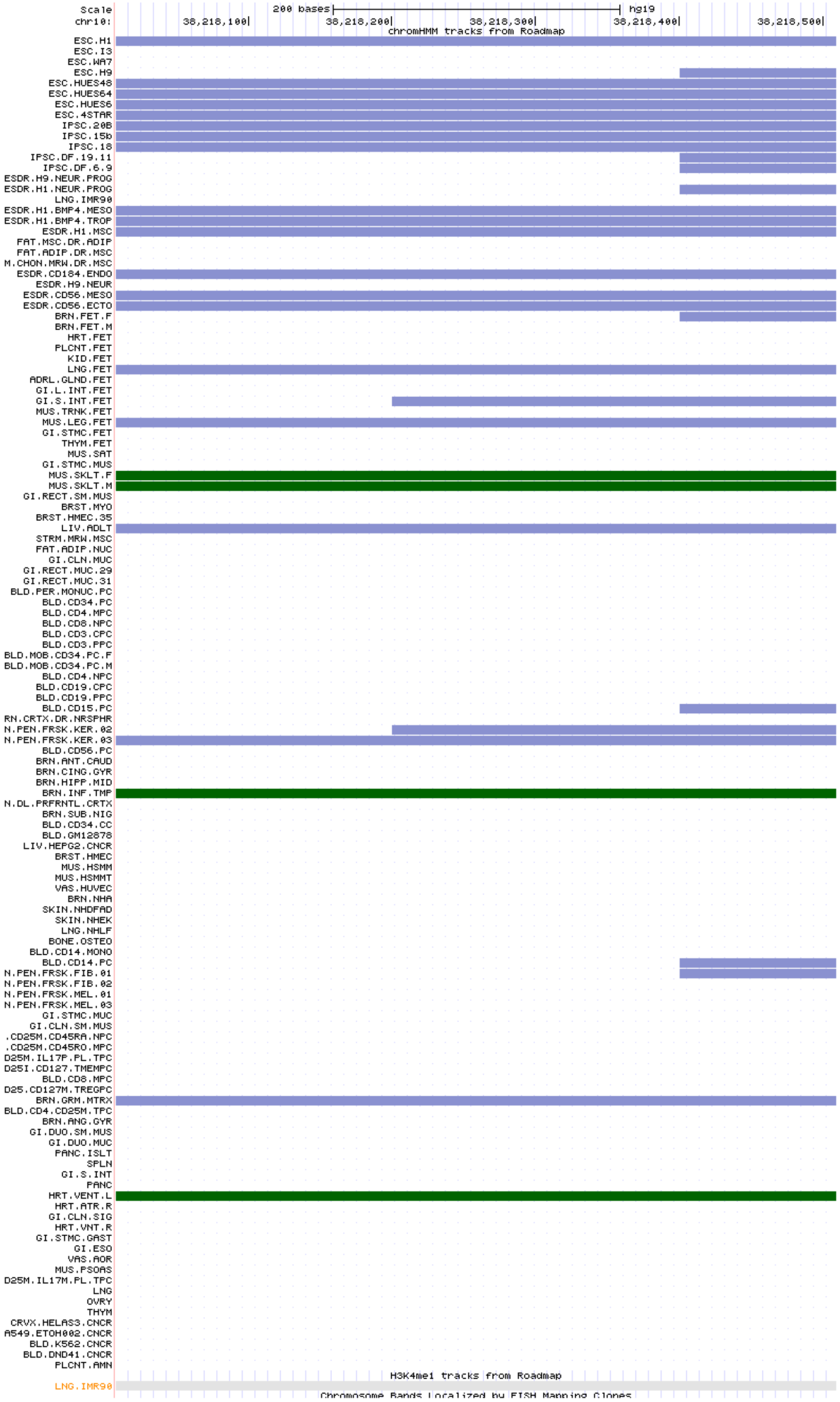

Supplementary Figure 9: Sensitivity analysis for IEAA susceptibility loci at 6p22.3

Below, we present the sensitivity analysis for assessing the association of GWAS SNPs with IEAA in 6p22.3. We compare the association results with and without adjustment for cg22736354. We performed the analyses using the WHI BA23 cohort stratified by study (studies 3, 4, 12 and 14) then combined the results by fixed effect models weighted by inverse variance. The panel (a) displays the 4 individual results: GARNET, WHIMs, SHARe AFR and SHARe AMR. The association results are marked in red with adjustment and marked in blue without adjustment. The CpG site is marked in black. The panel (b) displays the meta-estimates that combine the 4 results.

a: adding cg22736354 as a covariate

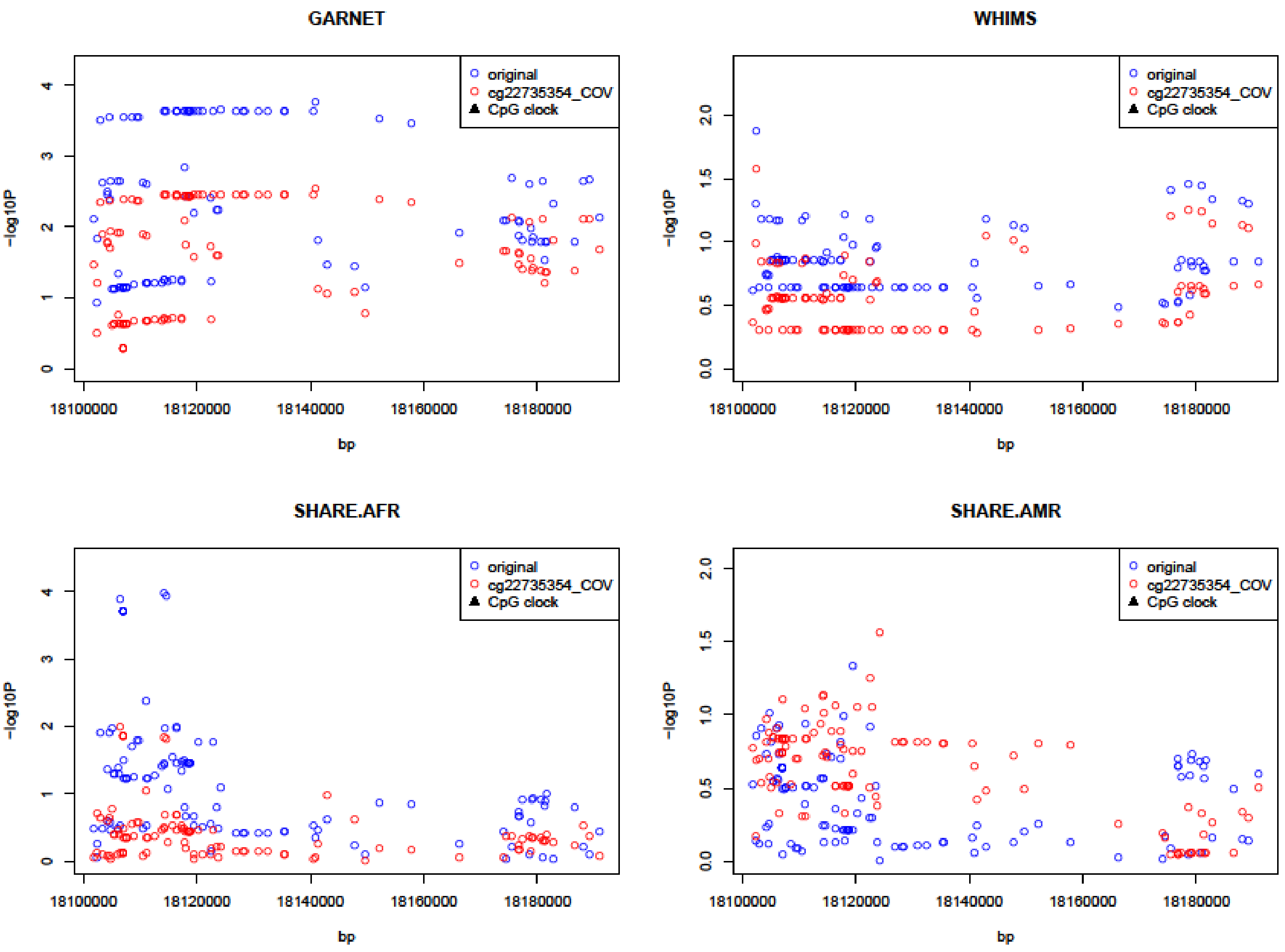

b: Meta-analysis using the WHI cohort

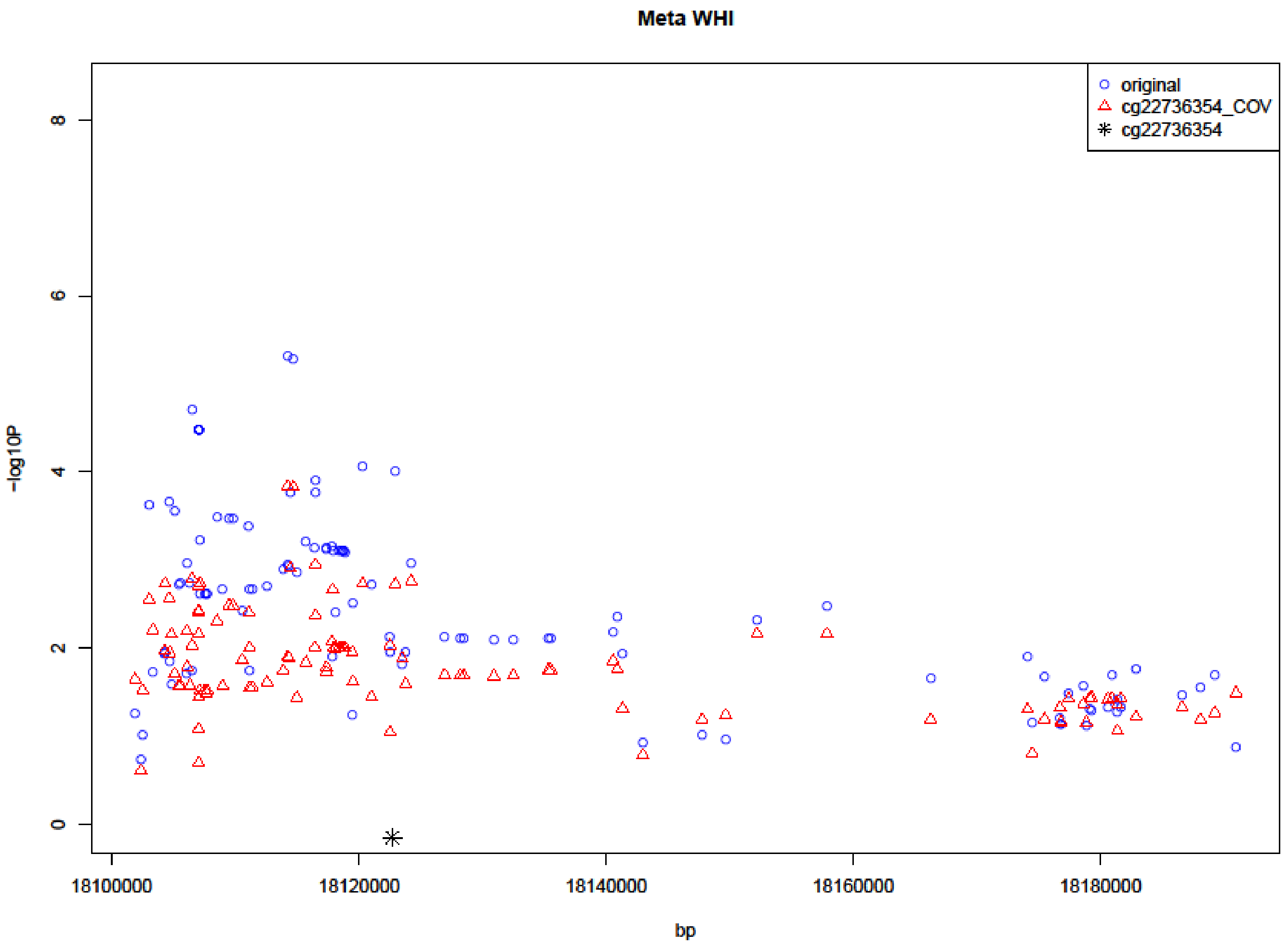

Supplementary Figure 10: Sensitivity analysis for IEAA susceptibility loci at 6p22.2

Below, we present the sensitivity analysis for assessing the association of GWAS SNPs with IEAA in 6p22.2. We compare the association results with and without adjustment for cg06493994. We performed the analyses using the WHI cohort stratified by study (studies 3, 4, 12 and 14) then combined the results by fixed effect models weighted by inverse variance. The panel (a) displays the 4 individual results: GARNET, WHIMs, SHARe AFR and SHARe AMR. The association results are marked in red with adjustment and marked in blue without adjustment. The CpG site is marked in black. The panel (b) displays the meta-estimates that combine the 4 results.

a: adding cg06493994 as a covariate

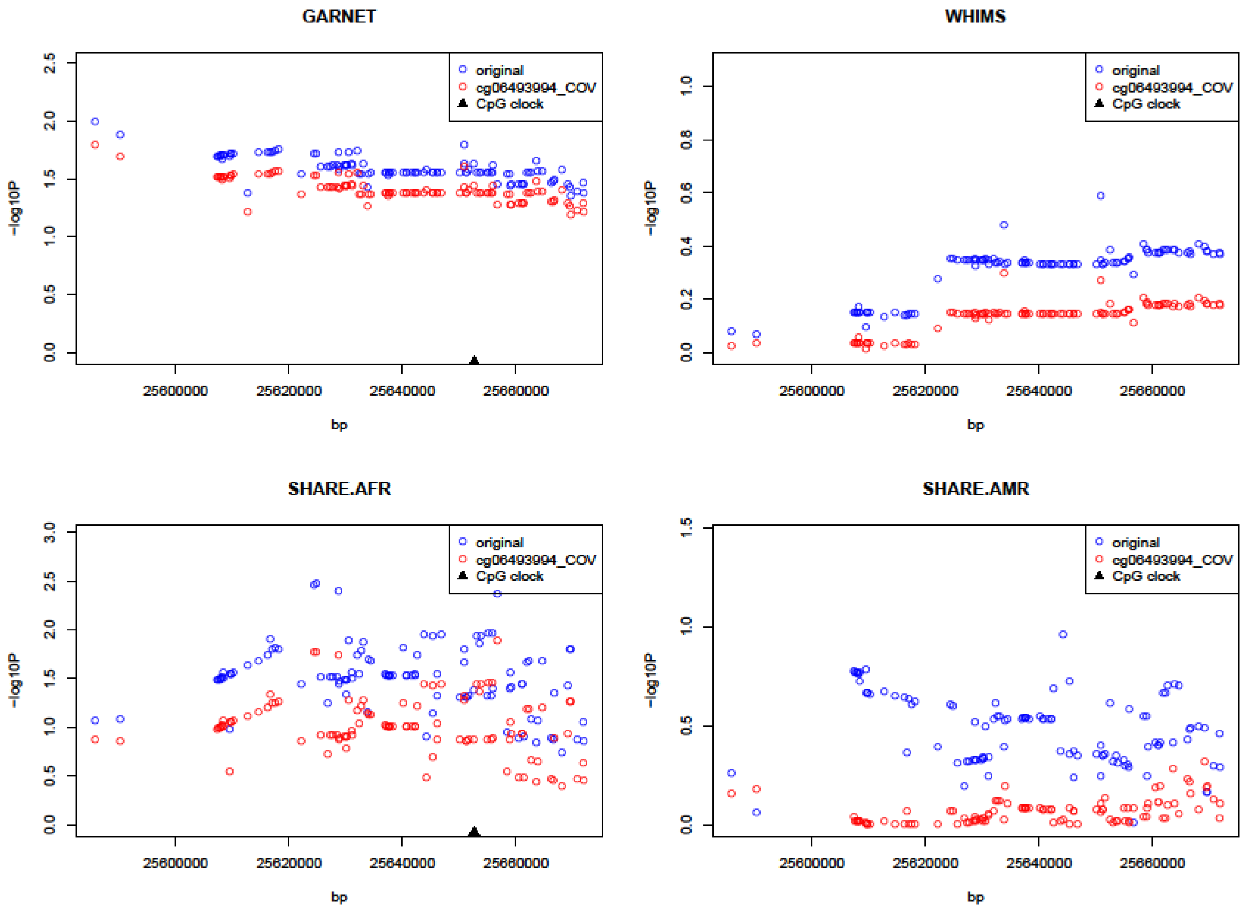

b: Meta-analysis using the WHI cohort

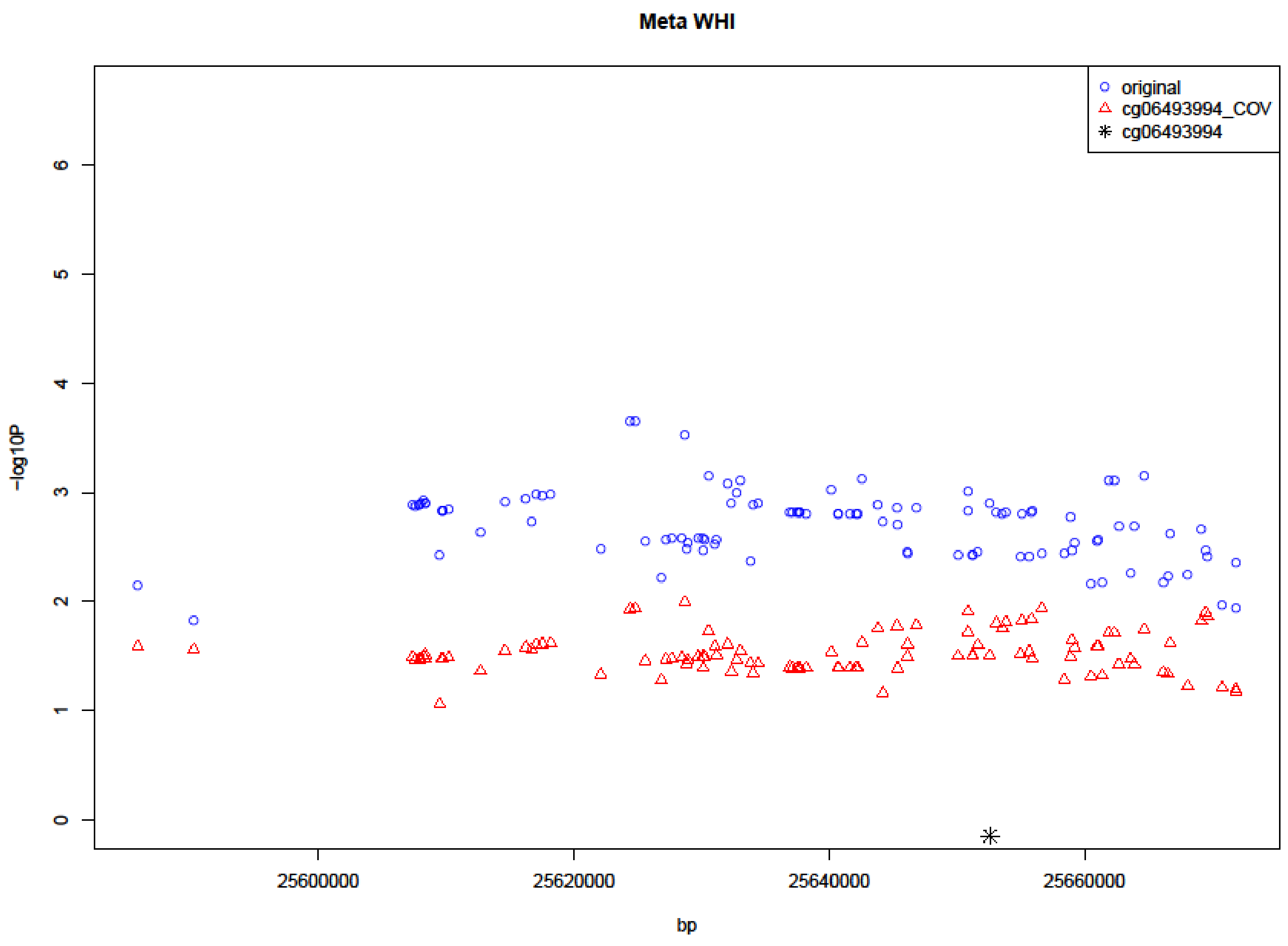

Supplementary Figure 11: Inspection of pleiotropic effect between IEAA and LTL in 5p15.33 *TERT* locus

To inspect the SNPs on TERT locus affecting IEAA independent of LTL, we re-conducted the association analysis in a subset of 785 individuals for whom both IEAA and LTL were available. All the individuals were collected from the WHI cohort including 457 individuals (from studies 3 & 5) with European (EUR) ancestry and 328 individuals (from study 12) with Africans ancestry (AFR). Panel (a) depicts the scatter plot of IEAA and LTL, stratified by ancestry group, indicating no association between these two aging measures. Panel (b) displays the association results at *TERT* locus for IEAA and IEAA adjusted LTL. It indicates that the SNP association for IEAA is independent of LTL at the *TERT* locus.

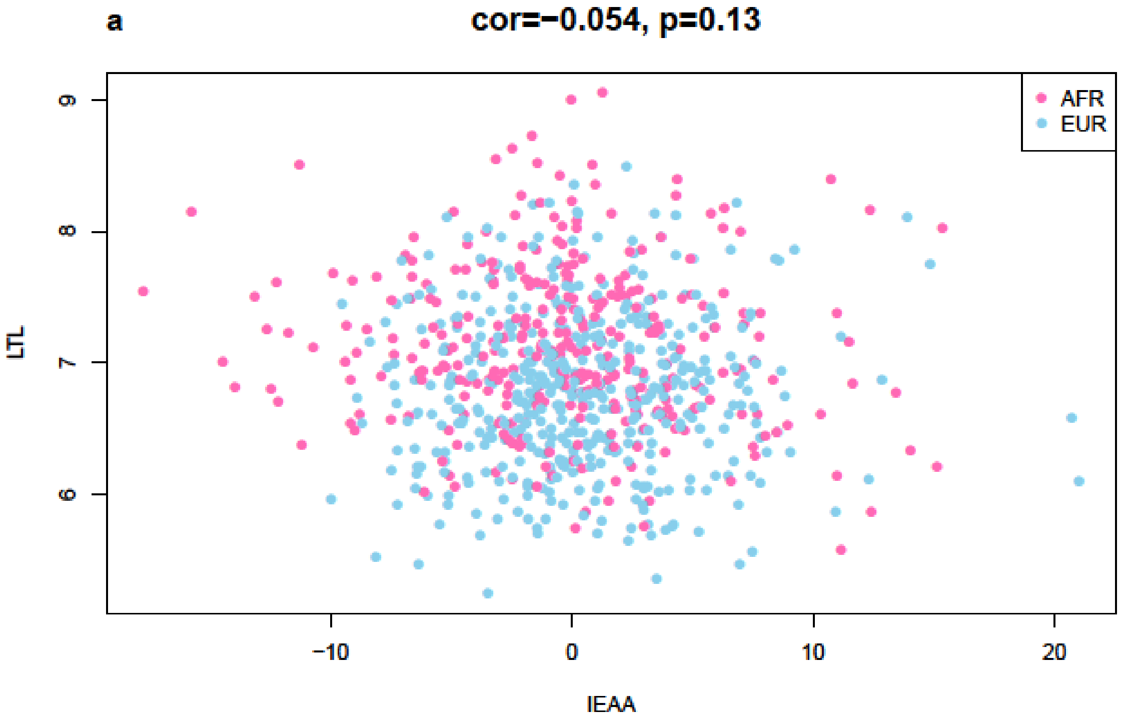
a
cor=−0.054, p=0.13
AFR
EUR
LTL
IEAA
9
8
7
6
−10
0
10
20

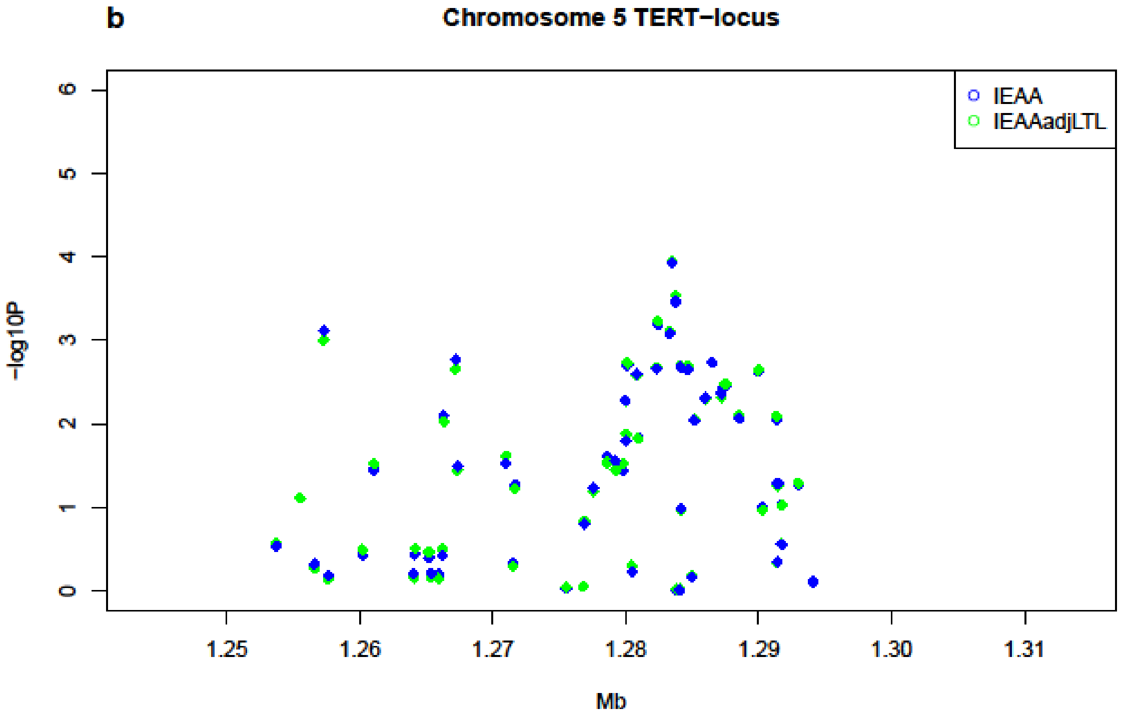
b
Chromosome 5 TERT−locus
IEAA
IEAAadjLTL
−log10P
Mb
6
5
4
3
2
1
0
1.25
1.26
1.27
1.28
1.29
1.30
1.31

Supplementary Figure 12: Diagrams under SMR analysis

The diagrams depict different scenarios among causal variant(s) (presented by *cis*-SNPs), the test exposure and phenotype in SMR analysis in conjunction with a HEIDI test[2]. The models under a significant SMR P value indicate two scenarios (a) causality association and (b) pleiotropy association. We perform a HEIDI test after observing a significant SMR P value. The model (c) indicates the alternative hypothesis that the test exposure and outcome variables are affected by two different causal variants in linkage disequilibrium (LD), according to a significant HEIDI P value.

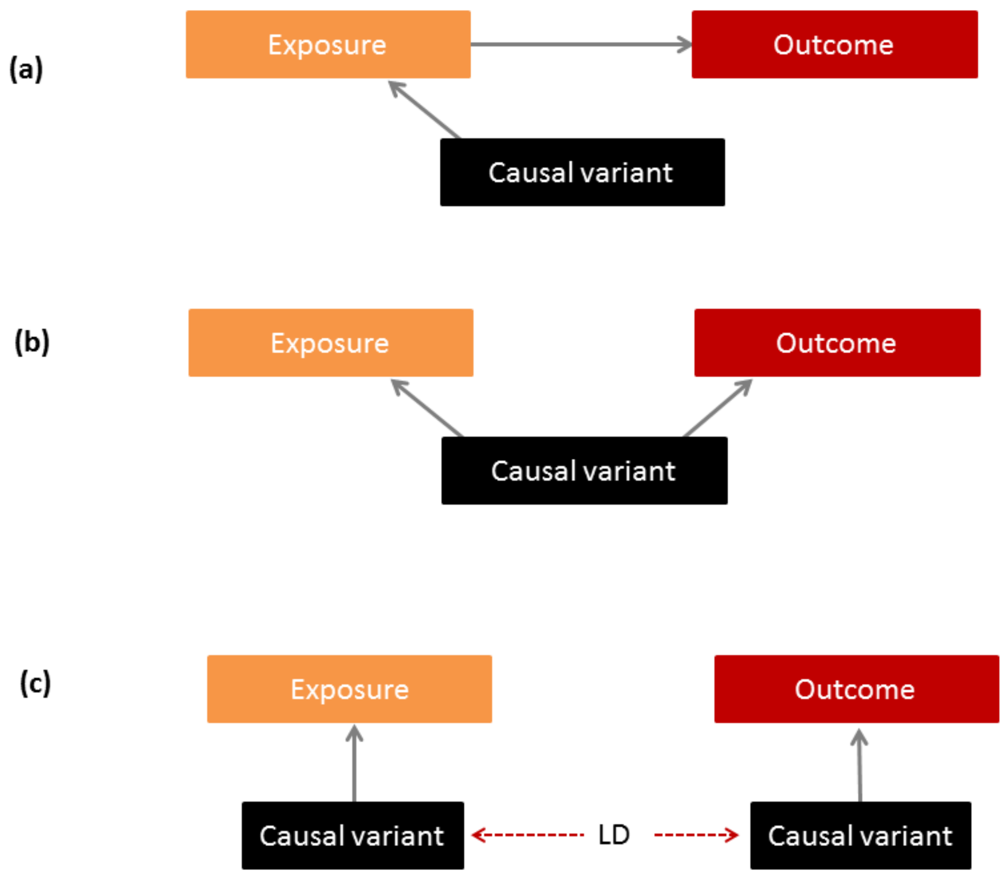

Supplementary Figure 13: Scatter plot of IEAA-association versus LTL-association in 5p15.33 *TERT* locus

The figure presents the association results of IEAA from our meta-analysis versus the association results of leukocyte telomere length (LTL) from Bojesen et al. using 53,724 individuals with European ancestry[3]. We pruned 13 SNPs associated with LTL at P < 1.0×10^{-5}, which are not in close proximity of the LTL-associated leading SNP rs7705526 (0.02 < LD r^2 < 0.2). The markers with positive (negative) effect sizes associated with LTL are marked in blue (red). Our analysis shows that there is a strong positive correlation between the two association patterns (Pearson' correlation coefficient =0.884and P=3.3×10^{-4}). More importantly, it indicates that the risk alleles associated with IEAA are all positively associated with LTL.

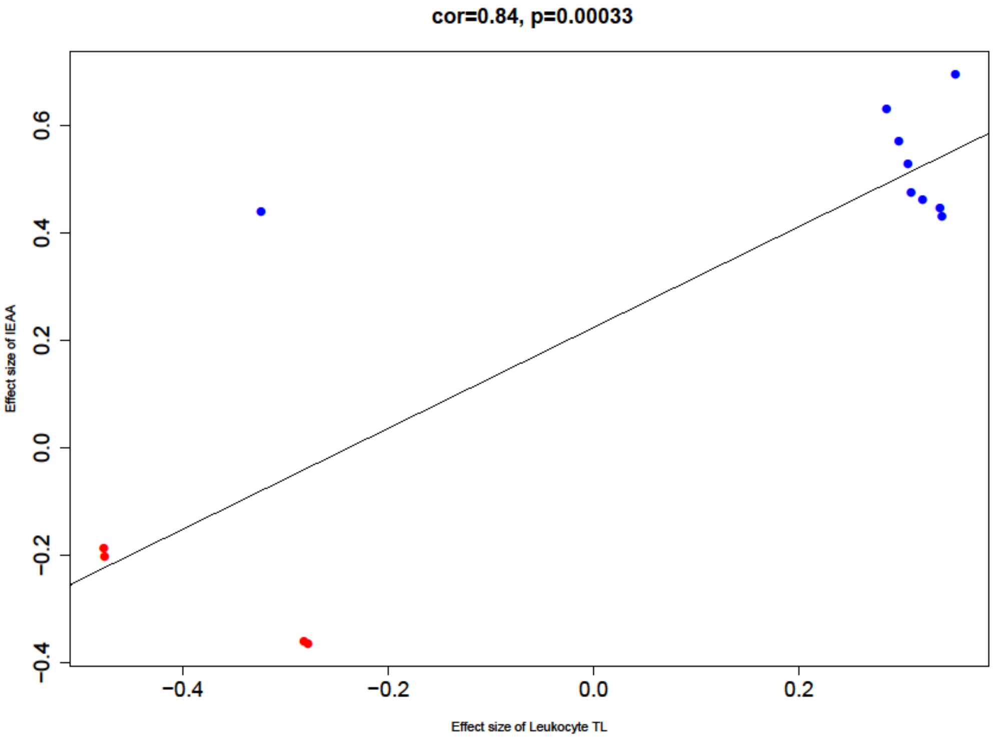

Supplementary Figure 14: Scatter plot of telomere length versus imputed cell counts in blood

The plot displays correlation analysis between LTL and a total of eight imputed cell counts. The analysis was performed on a subset (N=785) form the WHI cohort, including 457 individuals with European ancestry (marked in blue) and 328 individuals with Africans ancestry (marked in pink) that have both IEAA and LTL measures. Each cell count variable was adjusted for chronological age. Abbreviations for cell counts are listed in the following: nature killer (NK), monocyte (MONO) and granulocyte (Gran).

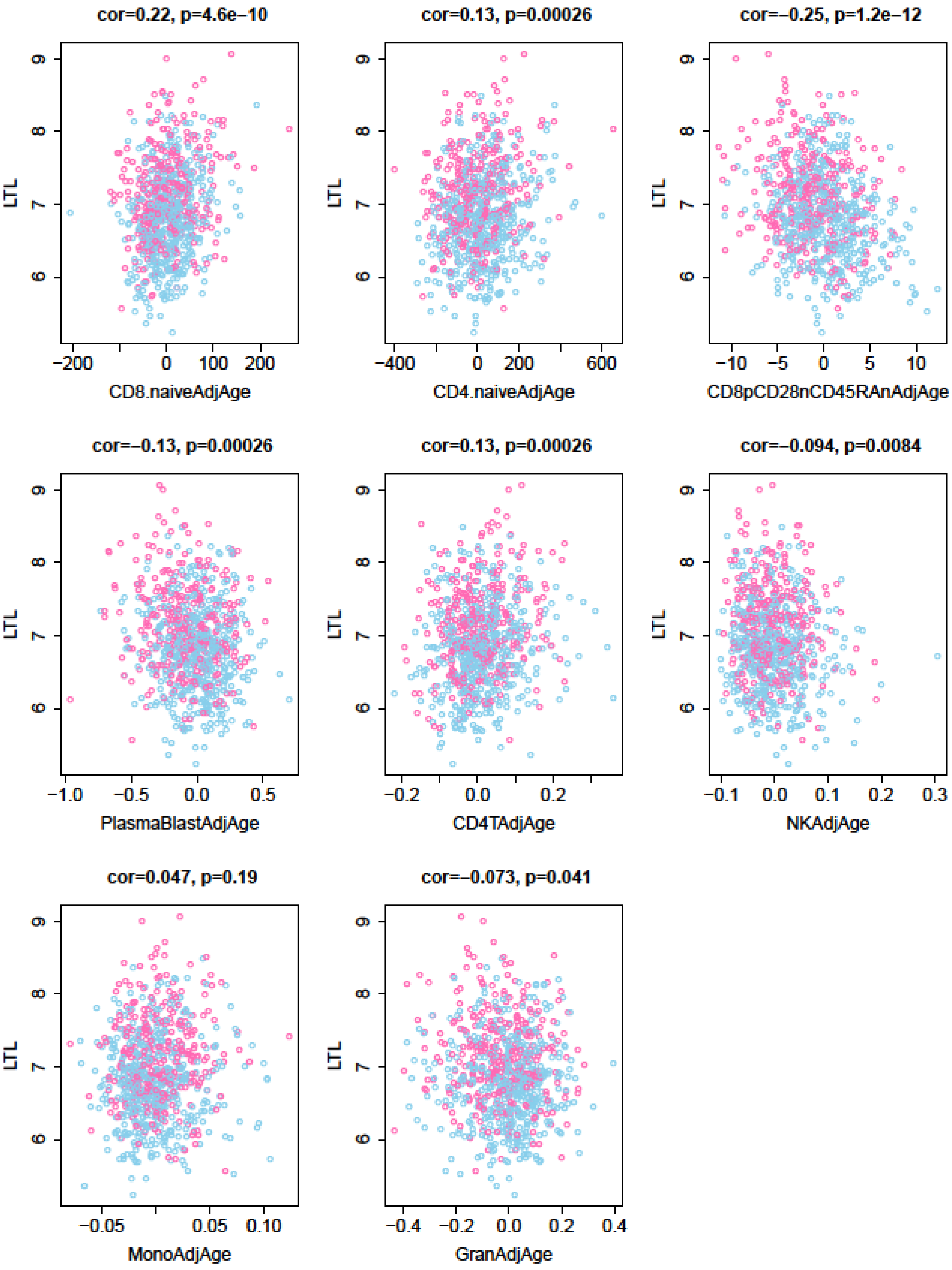
cor=0.22, p=4.6e−10
LTL
CD8.naiveAdjAge
cor=0.13, p=0.00026
LTL
CD4.naiveAdjAge
cor=−0.25, p=1.2e−12
LTL
CD8pCD28nCD45RAnAdjAge
cor=−0.13, p=0.00026
LTL
PlasmaBlastAdjAge
cor=0.13, p=0.00026
LTL
CD4TAdjAge
cor=−0.094, p=0.0084
LTL
NKAdjAge
cor=0.047, p=0.19
LTL
MonoAdjAge
cor=−0.073, p=0.041
LTL
GranAdjAge

Supplementary Figure 15: Scatter plot of IEAA-association versus CellCounts-association in 5p15.33 *TERT* locus

The figure displays the scatter plots of IEAA-SNP versus CellCounts-SNP association results based on effect sizes in the *TERT* locus. A total of eight imputed cell counts were analyzed. Abbreviations for cell counts are listed in the following: nature killer (NK), monocyte (MONO) and granulocyte (Gran).

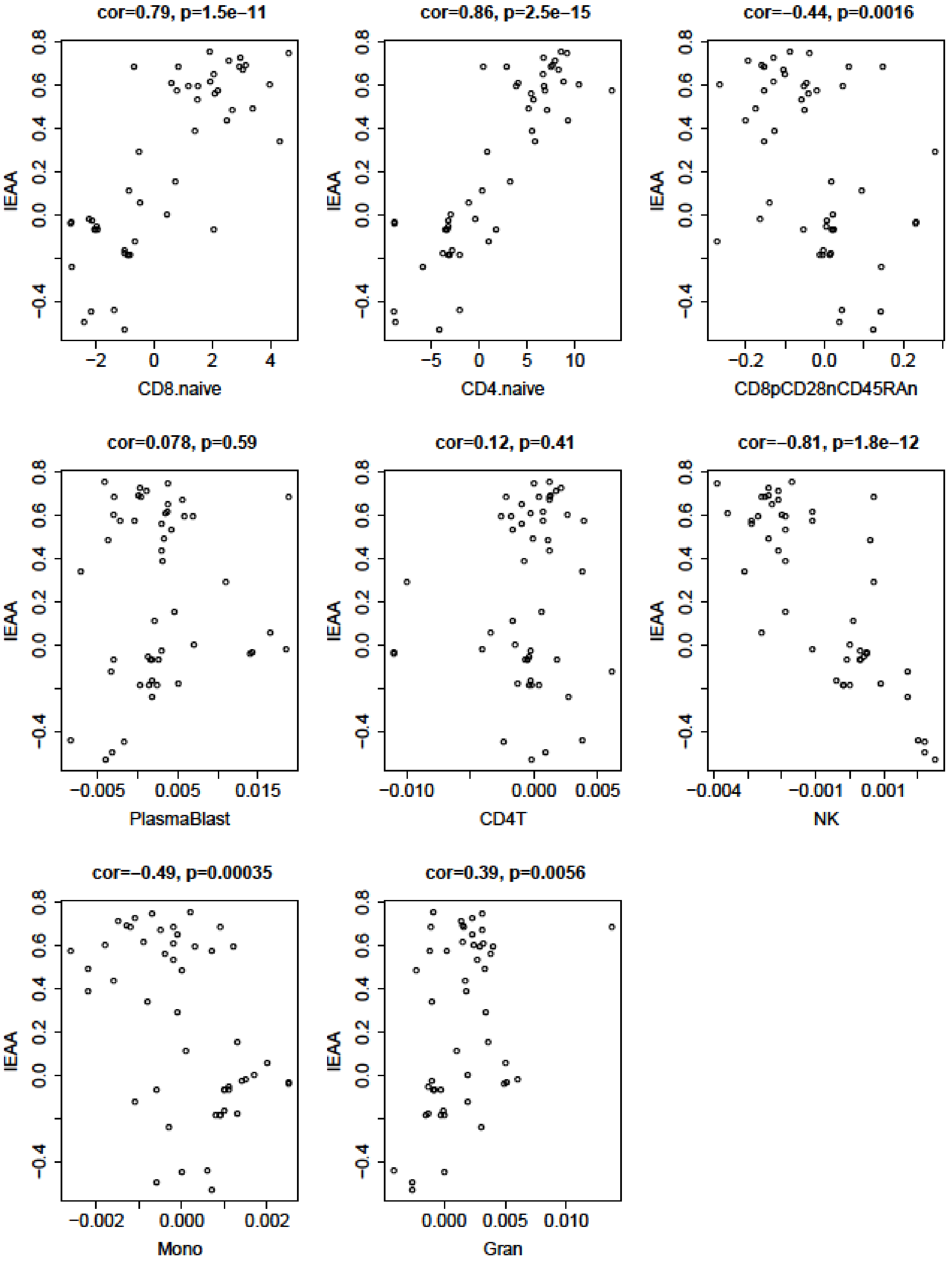
cor=0.79, p=1.5e−11
cor=0.86, p=2.5e−15
cor=−0.44, p=0.0016
cor=0.078, p=0.59
cor=0.12, p=0.41
cor=−0.81, p=1.8e−12
cor=−0.49, p=0.00035
cor=0.39, p=0.0056
IEAA
CD8.naive
CD4.naive
CD8pCD28nCD45RAn
PlasmaBlast
CD4T
NK
Mono
Gran

Supplementary Figure 16: Scatter plot of EEAA-association versus CellCounts-association in 5p15.33 *TERT* locus

The figure displays the scatter plots of EEAA-SNP versus CellCounts-SNP association results base on effect sizes, in *TERT* locus. A total of eight imputed cell counts were analyzed. Abbreviations for cell counts are listed in the following: nature killer (NK), monocyte (MONO) and granulocyte (Gran).

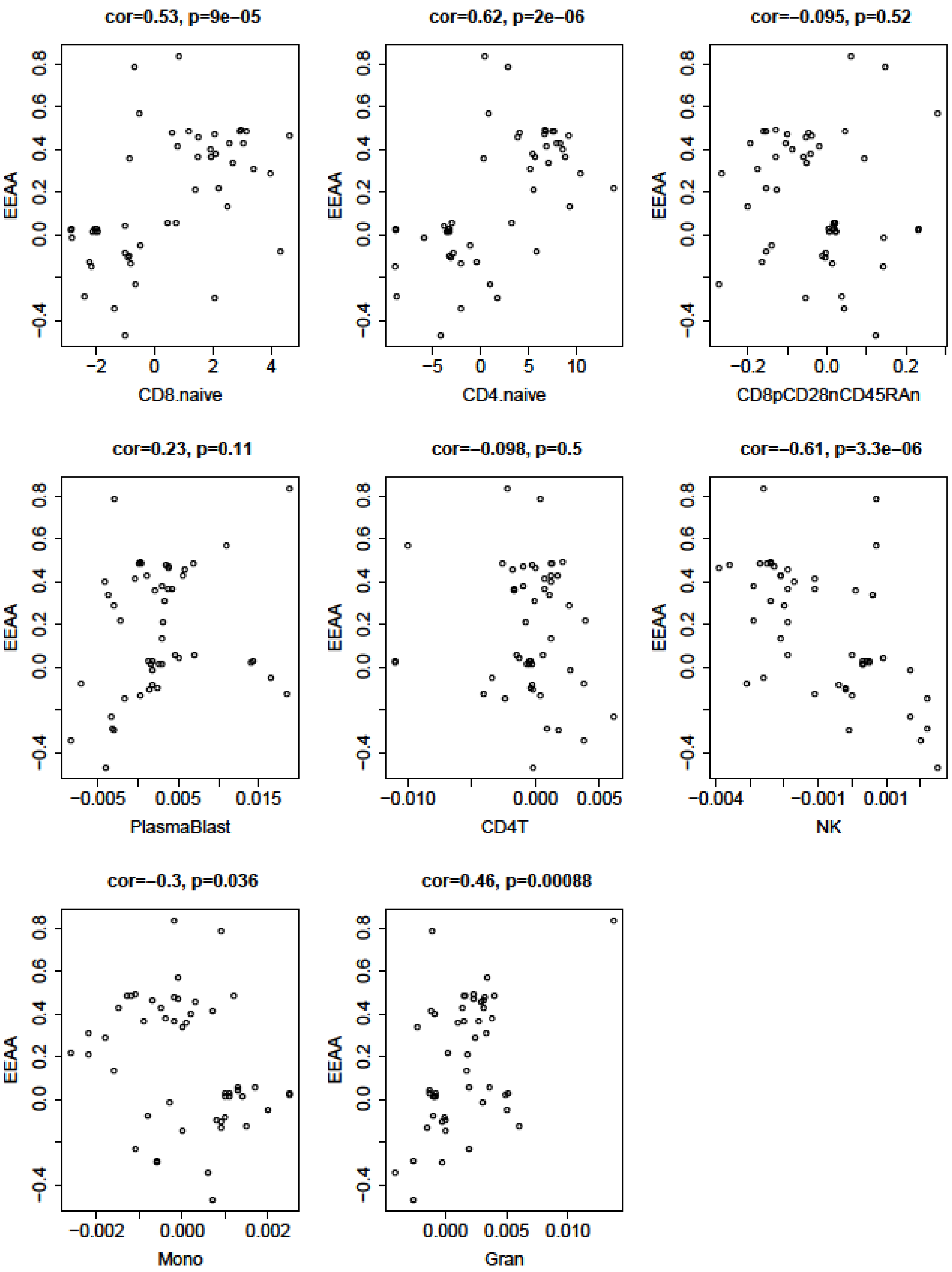
cor=0.53, p=9e−05
EEAA
CD8.naive
cor=0.62, p=2e−06
EEAA
CD4.naive
cor=−0.095, p=0.52
EEAA
CD8pCD28nCD45RAn
cor=0.23, p=0.11
EEAA
PlasmaBlast
cor=−0.098, p=0.5
EEAA
CD4T
cor=−0.61, p=3.3e−06
EEAA
NK
cor=−0.3, p=0.036
EEAA
Mono
cor=0.46, p=0.00088
EEAA
Gran

Supplementary Tables

Supplementary Table 1: Overview of study datasets

Description of the 15 study data sets (based on 9 cohorts) that were used in our GWAS of epigenetic age acceleration in blood. Both SNP array and DNA methylation array measurements in blood were available for each of the *N*=9,907 individuals. The WHI cohort corresponds to 6 genetic studies distinguished by study site (EMPC and BA23), sub-study and genetic ancestry. The Lothian Birth Cohorts corresponds to two distinct studies (distinguished by birth year). The last three columns report pairwise Pearson's correlation coefficients between chronological age and two estimates of DNAm age (Horvath[4] and Hannum[5]).

Study Index	Cohort	sub-study[a]	Genetic ancestry	N	Prop. Female	Age	Correlation (Age, DNAm Age) Horvath	Hannum	Correlation DNAm Ages Horvath vs Hannum
Stage 1 European									
**1**	**FHS**	--	EUR	2471	54.6%	66.4±9.0 [40,92]	0.79	0.83	0.83
**2**	**TwinsUK**	--	EUR	492	100%	56.0±9.0 [19, 79]	0.80	0.80	0.88
**3**	**WHI**	GARNET[b]	EUR	571	100%	67.1±6.7 [51,80]	0.69	0.76	0.75
**4**	**WHI**	GARNET[c]	EUR	420	100%	65.1±6.9 [49, 82]	0.77	0.78	0.82
**5**	**WHI**	WHIMS[b]	EUR	397	100%	70.1±5.2 [52, 80]	0.63	0.68	0.68
**6**	**EPIC-NORFOLK**	--	EUR	1117	49.7%	60.3±8.7 [40, 78]	0.74	0.83	0.74
**7**	**BLSA**	--	EUR	501	45.9%	70.9±14.1 [26, 98]	0.89	0.92	0.91
**8**	**inCHIANTI**	--	EUR	444	54.3%	62.4±15.9 [21, 91]	0.92	0.93	0.93
**9**	**BSGS**	--	EUR	614	49%	21.3±14.1 [10,75]	0.98	0.97	0.97
**10**	**LBC1921**	1921 cohort	EUR	446	60.5%	79.1 ±0.6 [78, 81]	0.11[d]	0.09[d]	0.60
**11**	**LBC1936**	1936 cohort	EUR	920	49.5%	69.6 ±0.8 [68, 71]	0.14[d]	0.15[d]	0.50
Stage 2 Non-European									
**12**	**WHI**	SHARe[b]	AFR	657	100%	62.9±6.6 [50, 80]	0.68	0.74	0.80
**13**	**WHI**	SHARe[c]	AFR	366	100%	62.6±6.9 [50, 81]	0.77	0.77	0.82
**14**	**WHI**	SHARe[b]	AMR	328	100%	62.4±6.9 [50, 80]	0.77	0.79	0.79
**15**	**WHI**	SHARe[c]	AMR	163	100%	61.2±6.1 [50,78]	0.70	0.71	0.79

[a] Substudy refers to separate genetic studies carried out within the WHI BA23 and EMPC respectively.
[b] WHI BA23 study.
[C] WHI EMPC study.
[d] The low correlation reflect that all subjects of the Lothian *Birth* Cohort had similar ages (within 3 years).
AFR=African, AMR=Americas including Hispanic ancestry, EUR=European.

Supplementary Table 2: Heritability analysis of intrinsic and extrinsic age acceleration

We used three software tools to estimate the heritability of IEAA and EEAA: (1) SOLAR [6], (2) LD Score regression (LDSC) [7], and (3) GCTA-REML[8]. **SOLAR –** The polygenic model implemented in SOLAR (Sequential Oligogenic Linkage Analysis Routine) software [6] was used to estimate heritability of IEAA and EEAA in the FHS pedigree cohort based on the known relationships. Heritability is defined as the total proportion of phenotypic variance attributable to genetic variation in the polygenic model. Both IEAA and EEAA measures were adjusted for sex prior to estimation of heritability. **LDSC** – We performed the LD Score regression analysis [7] to estimate the heritability of IEAA and EEAA using all European individuals in our study cohorts. As the approach only requires GWAS summary statistics rather than genotype type and we used our genome-wide meta-analysis results for estimation (studies 1-11, N=8393). LD scores were calculated based on the 1000 Genome European data (phase I) downloaded from LDSC for specifying independent variables and weights used in the regression. **GCTA** -We performed the REML analysis [9,10] to estimate the heritability of IEAA and EEAA using the postmenopausal women of the WHI cohort with non-European ancestry to estimate heritability of age acceleration in the populations of African (AFR) and America (AMR including Hispanic) ancestry. The WHI sub-studies were genotyped on different platforms. In order to combine the genotype data across the studies from WHI EMPC and WHI BA23, we converted the MaCH dosage format into PLINK format with best guess genotypes and used both genotyped and imputed markers for analysis (as detailed in "Imputation" below). We only used the overlapped markers existing in all studies (such that SNP missing rate=0) and controlled the quality of SNPs based on MAF > 0.05, Hardy Weinberg equilibrium (HWE) P > 0.0001, and MaCH $r^2 > 0.8$, yielding approximately 4M markers available for analysis. In particular, we increased the threshold of MAF to 10% in estimating the heritability of the AMR group in order to ensure the convergence of the REML estimation procedure. All analyses were adjusted for 4 principal components (PC).

We note of the relatively low h^2 estimates in the population of WHI Hispanics. According to a GCTA-GREML power analysis[10], at least 5,900 samples are needed to reach a statistical power of 80% for detecting a heritability of 10% at a 0.05 significance level. The low estimates could be biased by the small sample size in addition to the true heritability is small.

In particular, the intercept is 1.004 for the LDSC analysis for IEAA and the intercept is 1.011 for the LDSC analysis for EEAA. The intercept estimate minus one is an estimate for genomic inflation, indicating our GWAS results are not biased by the inflation.

		**IEAA**		**EEAA**	
**Cohort**	**Genetic ancestry**	h^2**(SE)**	***P***	h^2 **(SE)**	***P***
**FHS**	**EUR (n=2,478)**	0.37 (0.01)	$6.5x10^{-9}$	0.33 (0.01)	$4.0x10^{-7}$
**ALL EUR**	**EUR (n=8,393)**	0.19 (0.06)	$1.6x10^{-3}$	0.19 (0.06)	$2.8x10^{-3}$
**WHI**	**AFR(n=1,023)**	0.70 (0.27)	$7.0x10^{-3}$	0.27 (0.26)	0.17
**WHI**	**AMR[a] (n=491)**	0.12 (0.43)	0.44	0.05 (0.43)	0.40

[a] based on common SNPs (minor allele frequency ≥ 0.10). AMR denotes Americas.

Supplementary Table 3: Genomic platforms in the different studies

Study Index	Cohort	subgroup	SNP platform (accession number)	DNA methylation Platform (accession number)
**1**	**FHS**	--	Affymetrix 500k and MiPs 50K gene-centered chip (phs000342)	Illumina 450K (phs000724)
**2**	**UK twins**	--	Illumina HumanHap300, HumanHap610Q, 1M-Duo and 1.2MDuo 1M chips	Illumina 450K (GSE62992)
**3, 4**	**WHI**	GARNET	Illumina HumanOmni1-Quad v1-0 B (phs000200.v10.p3)	Illumina 450K (phs000200.v10.p3)
**5**	**WHI**	WHIMS	Illumina HumanOmniExpressExome-8v1_B (phs000200.v10.p3)	Illumina 450K (phs000200.v10.p3)
**6**	**EPIC-Norfolk**	--	Affymetrix Axiom UKBiobank Chip	Illumina 450K
**7**	**BLSA**	--	Illumina 550K	Illumina 450K
**8**	**inChianti**	--	Illumina 550K	Illumina 450K
**9**	**BSGS**	--	Illumina Human610-Quad	Illumina 450K (GSE56105)
**10**	**LBC**	1921 cohort	Illumina Human610-Quad	Illumina 450K (phs000821.v1.p1)
**11**	**LBC**	1936 cohort	Illumina Human610-Quad	Illumina 450K (phs000821.v1.p1)
**12, 13**	**WHI**	SHARE AFR	Affymetrix 6.0 (phs000200.v10.p3)	Illumina 450K (phs000200.v10.p3)
**14, 15**	**WHI**	SHARE AMR	Affymetrix 6.0 (phs000200.v10.p3)	Illumina 450K (phs000200.v10.p3)

AFR=Africans, AMR=Americas; accession number refers dbGaP study accession index or NCBI Gene Expression Omnibus.

Supplementary Table 4: SNPs used for imputation and GWAS

QC prior imputation

Study Index	Cohort	subgroup	#SNPs	MAF	HWE *P*	Minimal missing sample call rate	Genotyping rate
**1**	**FHS**	--	41,052	0.01	1e-06	3%	97%
**2**	**UK twins**	--	NA	0.01	1e-06	5%	98%
**3, 4**	**WHI**	GARNET	NA	None	1e-04	2%	98%
**5**	**WHI**	WHIMS	NA	0.01	1e-04	3%	97%
**6**	**EPIC-Norfolk**	--	705199	>0	1e-06	5%	95%
**7**	**BLSA**	--	51,4027	0.01	1e-04	1.5%	99%
**8**	**inChianti**	--	49,5343	0.01	1e-06	3%	99%
**9**	**BSGS**	--	530,922	0.01	1e-06	5%	95%
**10**	**LBC**	1921 cohort	535,709	0.01	1e-03	5%	98%
**11**	**LBC**	1936 cohort	535,709	0.01	1e-03	5%	98%
**12, 13**	**WHI**	SHARE AFR	NA	0.01	1e-06	5%	90%
**14, 15**	**WHI**	SHARE AMR	NA	0.01	1e-06	5%	90%

NA=not available.
HWE= Hardy-Weinberg equilibrium, MAF= minor allele frequency.

SNP imputation and GWAS analysis

	Imputation		GWAS QC and model					GWAS assessment[c]		
**Data**	**software**	**Ref**	**Info / R^2**	**genotype method**	**MAF**	**# PCA**	**GWAS software**	**#SNPs (common)**	**IEAA** $\lambda_{GC}/\lambda_{GC,c}$	**EEAA** $\lambda_{GC}/\lambda_{GC,c}$
Stage 1 European										
**Study 1 FHS**	Minimac/ MaCH	2012 March	0.4	Expected dosage	0.006	3	R KINSHIP	8,381,780 (5,992,597)	1.00/1.00	1.01/1.01
**Study 2 UK twins**	IMPUTE2	2011 June	0.4	Threshold at 0.90[a]	0.03	0	Plink	6,502,712 (5,757,213)	0.99/0.99	1.01/1.02
**Study 3 WHI GARNET BA23**	BEAGLE/ Minimac	2011 June	0.3	Expected dosage	0.026	0	MACH2qtl	7,538,309 (6,449,435)	1.01/1.01	1.00/1.00
**Study 4 WHI GARNET EMPC**	BEAGLE/ Minimac	2011 June	0.3	Expected dosage	0.036	2	MACH2qtl	7,175,106 (6,569,837)	1.00/1.00	1.01/1.01
**Study 5 WHI WHIMS BA23**	BEAGLE/ Minimac	2011 June	0.3	Expected dosage	0.038	0	MACH2qtl	6,848,760 (6,379,302)	1.00/1.00	1.02/1.02
**Study 6 EPIC-Norfolk**	IMPUTE2	2014 Dec	0.4	Expected dosage	0.013	5	SNPTEST v2.5	9,242,040 (6,972,383)	0.99/0.99	1.01/1.01
**Study 7 BLSA**	Minimac/ MaCH	2011 June	0.3	Expected dosage	0.03	0	MACH2qtl	7,159,920 (6,334,360)	1.06/1.06	1.06/1.07
**Study 8 inChianti**	Minimac/ MaCH	2011 June	0.3	Expected dosage	0.034	0	MACH2qtl	6927160 (6304233)	1.00/1.00	1.02/1.02
**Study 9* BSGS**	IMPUTE2	2012 March	0.8	Best guess[b]	0.024	0	MERLIN/ QTDT	6,325,157 (5,499,748)	1.01/1.01	1.02/1.03
**Study 10 LBC 1921**	Minimac/ Mach	2012 March	0.3	Dosage	0.036	4	MACH2qtl	6,898,072 (6,265,129)	1.00/1.00	1.01/1.01

**Study 11 LBC 1936**	Minimac/ Mach	2012 March	0.3	Dosage	0.016	4	MACH2qtl	7,973,627 (6,269,234)	1.00/1.00	0.99/0.99
Stage 2 non-European										
**Study 12 WHI SHARE AFR (BA23)**	BEAGLE/ MaCH	2011 June	0.3	Expected dosage	0.023	2	MACH2qtl	10,886,445 (8,063,329)	--	--
**Study 13 WHI SHARE AFR (EMPC)**	BEAGLE/ MaCH	2011 June	0.3	Expected dosage	0.041	2	MACH2qtl	8,838,152 (8,157,491)	--	--
**Study 14 WHI SHARE AMR(BA23)**	BEAGLE/ MaCH	2011 June	0.3	Expected dosage	0.046	2	MACH2qtl	8,355,615 (8,059,799)	--	--
**Study 15 WHI SHARE AMR (EMPC)**	BEAGLE/ MaCH	2011 June	0.3	Expected dosage	0.09	2	MACH2qtl	6,186,837 (6,186,837)	--	--

PCA= principal component.

[a] Minimum number of samples per marker level was required at 30 and threshold of HWE *P* value was set at 1.0 x 10^{-6} for conducting association analysis.

[b]Threshold of HWE *P* value was set at 1.0 x 10^{-6} for conducting association analysis.

Genomic inflation was only estimated for stage 1 analysis, which were involved genome-wide association tests.

Studies 1 and 7 are family based datasets.

Ref.=Dates of 1000 genome haplotypes released.

Info/R^2 refers to IMPUTE2 info measure and MaCH/Minimac R^2, respectively.

Thresholds of MAF were determined accordingly to study sample sizes that allow numbers of minor allele ≥ 30 in association analysis.

#SNPs (common) =total number of markers (limited to common variants MAF ≥ 0.05).

λ_{GC}=genomic inflation estimated based on all SNPs.

$\lambda_{GC,c}$=genomic inflation estimated based on all SNPs with common variants.

Supplementary Table 5: Summary of SMR analysis on *c*is-genes associated with epigenetic age acceleration in blood

The tables below present SMR analysis results using our meta GWAS scores based on all studies. The SMR analysis in conjunction with HEIDI (heterogeneity in dependent instruments) test was performed on using our GWAS summary data and the eQTL analysis results from FHS, GTEx, and LSMeta studies, respectively. The null hypothesis under HEIDI ($P_{HEIDI} \geq 0.01$) supports that the test exposure and outcome variables are affected the same causal variant. We list the summary statistic Z and P_{SMR} for the SMR analysis, P_{HEIDI}, **EUR** P_{HEIDI}, and **N**$_{SNP}$ for the HEIDI analysis. The HEIDI analysis show the four genes: *KPNA4*, *STXBP4*, *RNF4* and *HSD17B7P2* at $P_{HEIDI} > 0.01$. As LD pattern involved in HEIDI estimation, we re-performed the HEIDI analysis using the GWAS scores based on the studies of European ancestry (studies 1-11) for sensitivity check, referred as to **EUR** P_{HEIDI}. The **EUR** P_{HEIDI} values overall agree with the P_{HEIDI} values. The column **N**$_{SNP}$ lists the number of SNPs used in the HEIDI analysis.

		FHS					GTEx					LSMeta				
**CHR**	**Gene**	**Z**	P_{SMR}	P_{HEIDI}	**EUR** P_{HEIDI}	**N**$_{SNP}$	**Z**	P_{SMR}	P_{HEIDI}	**EUR** P_{HEIDI}	**N**$_{SNP}$	**Z**	P_{SMR}	P_{HEIDI}	**EUR** P_{HEIDI}	**N**$_{SNP}$
**IEAA**																
3	*KPNA4*	4	5.1E-05	6.5E-02	1.6E-01	199	--	--	--	--	--	--	--	--	--	--
6	*TPMT*	5.3	1.1E-07	6.7E-06	1.6E-05	79	4.7	2.2E-06	2.8E-10	6.5E-11	32	5.1	3.5E-07	2.2E-03	1.0E-03	89
17	*STXBP4*	3.4	6.2E-04	1.9E-02	3.0E-02	647	--	--	--	--	--	--	--	--	--	--
**EEAA**																
4	*RNF4*	--	--	--	--	--	--	--	--	--	--	3.1	2.0E-03	3.1E-02	3.0E-02	59
10	*ZNF25*	3	2.8E-03	3.2E-04	2.9E-04	588	--	--	--	--	--	--	--	--	--	--
10	*HSD17B7P2*	--	--	--	--	--	4.5	6.9E-06	3.9E-01	3.2E-01	843	--	--	--	--	--

-- denotes not available.

Supplementary Table 6: Summary of SMR analysis of telomere length effect on IEAA

The table below presents SMR analysis for testing the pleiotropic associaiton of SNPs in 5p15.33 *TERT* -> telomere length -> IEAA. We used the summary data from two large-scale meta analysis studies for association of leukocyte telomere length (LTL). The first study was performed by Bojesen et al. using 53,724 individuals[3] and the second study was performed by Codd et al. using 37,684 individuals[11]. Both study populations are with European ancestry. We selected the SNPs (located in 5p15.33 *TERT*) associated with LTL at P< 1.0E-05 as instrumental variables in SMR analysis. We list the summary statistic β, SE, Z and P_{SMR} for the SMR analysis as well as P_{HEIDI}, and **EUR** P_{HEIDI} for the HEIDI analysis.

Both studies indicate significant associations between LTL and IEAA with consistent directions, yielding a Stouffer's Meta P value at 1.7E-13. The positive signs of effect size (β) indicate that telomere length-increasing alleles (indicative of younger cell age) are associated with increased IEAA (indicative of older cell age). The HEIDI analysis further validates that both LTL and IEAA are affected by the same causal variant according to a non-significant P_{HEIDI} ($\geq$ 0.01). As LD pattern involved in HEITI estimation, we re-performed the HEIDI analysis for sensitivity check using the GWAS scores of IEAA based on the studies of European ancestry (studies 1-11), referred as to **EUR** P_{HEIDI}. In the second study, the sensitivity analysis shows a moderate difference between the two P values (P_{HEIDI}, and **EUR** P_{HEIDI}) but still supports the null hypothesis that both LTL and IEAA are affected by the same causal variants.

Study	β	SE	Z	P_{SMR}	P_{HEIDI}	EUR P_{HEIDI}
Bojesen et al.	1.19	0.23	5.07	3.9E-07	0.18	0.17
Codd et al.	6.22	1.12	5.35	8.9E-08	0.12	0.04

Supplementary Table 7: SNPs in 5p15.33 *TERT* associated with IEAA and leukocyte telomere length both at genome-wide significance

The table presents six SNPs associated with IEAA and leukocyte telomere length (LTL) both at P< 5.0E-08. The association results for IEAA are based on the combined phase across all our 15 studies while the association results for LTL are queried from a large-scale meta-analysis performed by Bojesen et al (N=53,724)[3].

Annotation				LTL ($x10^{-3}$)			IEAA		
**SNP**	**bp**	**Test allele**	**MAF**	**Beta***	**SE**	**P**	**Beta***	**SE**	**P**
rs4449583	1284135	T	0.33	0.488	0.066	1.9E-13	0.685	0.099	4.1E-12
rs4975538	1280830	C	0.37	0.374	0.065	7.1E-09	0.605	0.103	3.6E-09
rs7705526	1285974	A	0.33	0.509	0.067	2.3E-14	0.606	0.089	1.0E-11
rs7725218	1282414	A	0.35	0.419	0.065	1.1E-10	0.632	0.093	1.0E-11
rs7726159	1282319	A	0.34	0.474	0.066	4.5E-13	0.672	0.099	9.5E-12
rs7734992	1280128	C	0.43	0.377	0.063	2.2E-09	0.588	0.098	1.8E-09

*with respect to test alleles.

Supplementary Table 8: MR-Egger regression analysis for the associations of IEAA with leukocyte telomere length

The table below presents the genome-wide significant association results of IEAA aligned with the association results of leukocyte telomere length (LTL) conducted by Codd et al.[11]. Each SNP is a distinct leading variant in the corresponding band, as listed in **Table 1** in manuscript. We performed MR-Egger regression analysis using the 6 SNPs as instrumental variables and did not observe a causal effect of IEAA on LTL (causal (SE) = 0.02 (0.07) with P= 0.8 and pleiotropy (SE) = 0.003 (0.004) with P=0.9)

					Effect size (β)		Meta P value	
**Band**	**SNP**	**Gene**	**A1**	**MAF**	**IEAA**	**LTL**	**IEAA**	**LTL**
3q25.33	rs11706810	*TRIM59*	C	0.45	0.41	-0.01	1.6E-09	0.34
5p15.33	rs2736100	*TERT*	C	0.49	0.49	0.08	2.7E-11	4.1E-19
6p22.3	rs1142345*	*TPMT*	C	0.04	-1.61	-0.03	2.9E-21	0.20
	rs6915893	near *NHLRC1*	C	0.39	-0.52	-0.01	1.6E-13	9.4E-02
6p22.2	rs9467538	near *SCGN*	A	0.29	-0.46	0.001	2.6E-10	0.90
17q22	rs17817448	*SBXBP4*	T	0.22	-0.47	-0.0003	8.1E-09	0.97

*a surrogate of the leading SNP rs193093668, which is absent in the summary data of LTL.
A1: reference allele; MAF=minor allele frequency; Effect sizes corresponding additive model.

Supplementary Table 9: Multivariable regression analysis of hTERT expression implicated in DNAm age

We conducted multivariable regression analysis to study the effect of experimental-induced hTERT expression on Horvath DNAm age. In the experiment, we introduced a *TERT*-expressing vector or empty vector (as control) into primary fibroblasts isolated from human neonatal foreskin. In model I, we incorporated the effect of cell population doubling on DNAm age and observed that the association was highly modified by hTERT (interaction P=1.3E-05). Similarly, in model II, the association of cell passage number with DNAm age can be highly modified by hTERT (interaction P=1.6E-06).

Model	Variable	Estimate (SE)	P-value
	Intercept	32.4 (2.3)	2.0E-08
	hTERT	-8.1 (3.3)	0.03
	Population doubling	0.19 (0.08)	0.04
	hTERT x population doubling	0.65 (0.10)	1.3E-05
	Intercept	33.4 (2.0)	3.6E-09
	hTERT	-6.7 (3.0)	0.05
	Passage number	0.36 (0.16)	0.04
	hTERT x Passage number	2.1 (0.23)	1.6E-06

Supplementary Table 10: Functional enrichment study of SNP sets associated with IEAA/EEAA

Here we used the MAGENTA software to evaluate what is known about the set of SNPs associated with IEAA and EEAA, respectively. We present the MAGENTA analysis results stratified by cutoffs set at the GSEA algorithm (0.95 and 0.75). Each row corresponds to the significant results thresholded at a **FDR < 0.20** from the MAGENTA analysis.

**GSEA**	**GWAS**	**Data base**	**Pathway/Gene set**	**Size**	**Nominal $P_{MAGENTA}$**	**FDR***
**0.75**	**IEAA**	GO	mesoderm formation	22	2.0E-04	9.7E-02
		KEGG	Fc epsilon RI signaling pathway	71	3.0E-04	**2.7E-02**
		KEGG	Colorectal cancer	61	3.0E-04	**4.2E-02**
		PANTHER	5HT4 type receptor mediated signaling pathway	10	3.5E-03	1.1E-01
	**EEAA**	KEGG	Neurotrophin signaling pathway	119	2.0E-04	**4.2E-02**
		PANTHER BIO	Chromatin packaging and remodeling	147	5.0E-04	9.7E-02
		PANTHER MOL	Histone	34	1.3E-03	1.8E-01
		PANTHER	Ras Pathway	13	4.9E-03	1.9E-01
**0.95**	**IEAA**	PANTHER BIO	Nuclear transport	74	5.7E-05	**1.7E-02**
		PANTHER BIO	mRNA transcription elongation	24	6.2E-03	1.9E-01
	**EEAA**	PANTHER BIO	mRNA transcription elongation	25	1.0E-04	**1.1E-02**
		PANTHER BIO	mRNA transcription termination	7	3.3E-03	**1.8E-02**

*resulted from the MAGENTA algorithm and marked in bold when < 0.05.
PANTHER BIO=Panther biological process; PATHER MOL= Panther molecular function.

Supplementary Table 11: Telomere maintenance genes is enriched for IEAA associated genes

While our IEAA association overlaps with leukocyte telomere length association in *TERT* locus, our MAGENTA enrichment analysis with threshold set at 0.75 highlighted the gene set implicated in telomere maintenance at P=0.04. The gene set of telomere maintenance includes 20 genes. We list the detailed results of the 20 genes in ascending order based on their gene P values. In the MAGETA algorithm, the gene P values are determined by the best SNP with the most significant association P value in our GWAS among the markers within the gene. While a number of 5 genes was expected to have an observed P value beyond the 75th percentile of the P values for the entire set of human autosomal genes, we observed a total of 9 genes (marked in bold) beyond the threshold.

**Rank**	**Chr**	**Gene**	**Gene P value***	**Gene size (kb)**	**Number of SNPs**	**Best SNP**	**Best SNP P value**
1	5	***TERT***	<1.0E-16	42	437	rs2736099	**4.67E-12**
2	8	***PRKDC***	1.70E-02	187	150	rs200642161	**3.80E-04**
3	20	***RTEL1***	2.12E-02	38	480	rs201926957	**2.46E-04**
4	5	***RAD50***	3.30E-02	88	267	rs60153262	**5.84E-04**
5	6	***HSPA1L***	5.91E-02	5	215	rs201942311	**1.40E-03**
6	12	***PTGES3***	6.14E-02	25	171	rs199638911	**1.51E-03**
7	15	***BLM***	1.13E-01	98	557	rs199920634	**1.01E-03**
8	22	***XRCC6***	1.30E-01	43	172	rs67820209	**3.47E-03**
9	10	***OBFC1***	2.18E-01	41	390	rs35393286	**3.09E-03**
10	16	*TERF2*	3.50E-01	30	269	rs73564497	7.45E-03
11	17	*SMG6*	4.58E-01	244	837	rs2957925	6.06E-03
12	16	*ERCC4*	4.76E-01	32	379	rs113508033	7.06E-03
13	8	*NBN*	4.88E-01	51	324	rs80268568	1.20E-02
14	1	*PARP1*	5.44E-01	47	437	rs200562350	1.02E-02
15	10	*DCLRE1C*	5.92E-01	47	472	rs35141448	1.31E-02
16	16	*ACD*	5.98E-01	3	102	rs201413638	2.57E-02
17	2	*XRCC5*	6.85E-01	97	459	rs28565088	2.14E-02
18	8	*WRN*	7.83E-01	141	507	rs55851717	2.48E-02
19	16	*TERF2IP*	8.00E-01	10	323	rs199548999	4.23E-02
20	17	*C17orf68*	8.02E-01	23	270	rs67787905	4.56E-02

*reference P values adjusted for gene size, number of SNPs within a gene, and other confounders.

Supplementary Table 12: LDSC genetic correlation between epigenetic age acceleration and a broad category of other GWAS studies

The table reports the results from cross-trait LD Score regression (LDSC) [7] analysis with a broad category of large scale GWAS results for age-related outcomes or complex diseases using our GWAS results for IEAA/EEAA. For each test trait, we displays sample size, study population (genetic ancestry), genetic correlation estimate (r_g (SE)) and it P value. Abbreviations for genetic ancestry are listed in the following: Asians (ASN), Europeans (EUR) and ALL denotes more than two ancestry population. We order the traits according to category (I) GWAS of anthropometric traits conducted by GIANT consortium, (II) GWAS of lipid, metabolic, and inflammatory outcomes and diseases, (III) GWAS of neurodegenerative and neuropsychiatric disorders, (IV) cognitive functioning and educational attainment traits, and (V) longevity, reproductive ageing and mitotic clock related traits. Of the test traits, we constrained the intercepts with –intercept-h2 and intercept-gencov flags when a test trait with GC correction that we input the estimates of intercepts obtained from the heritability analysis implemented under LDSC. Constrain parameters are listed in the format, trait (constrain), as the following: waist circumference (0.7608), waist to hip ratio (0.8359), high density lipoprotein (0.9724), low density lipoprotein (0.9582), total cholesterol (0.9529), triglyceride (0.959), AMD (0.9469), AMD geographic atrophy (0.9671), AMD neovascular (0.9652), educational attainment (0.93), age at menopause (0.99), and leukocyte telomere length (0.9802). The intercepts for IEAA and EEAA were set at one when we constrained the intercept for the test trait.

Trait	N	POP	IEAA r_g (SE)	IEAA P	EEAA r_g (SE)	EEAA P
**Category (I)**						
Waist circumference	232101	EUR	0.10 (0.04)	**2.2E-02**	0.15 (0.047)	**9.0E-04**
Waist to hip ratio	212243	EUR	0.06 (0.05)	2.0E-01	0.14 (0.048)	**2.7E-03**
BMI*	339224	ALL	-0.01 (0.065)	8.8E-01	0.08 (0.071)	2.6E-01
Height	133858	EUR	-0.02 (0.052)	7.0E-01	0.13 (0.043)	**2.8E-03**
**Category (II)**						
High density lipoprotein	188577	EUR	-0.08 (0.05)	1.1E-01	-0.12 (0.05)	**1.7E-02**
Low density lipoprotein	188577	EUR	-0.049 (0.054)	3.7E-01	-0.017 (0.048)	7.2E-01
Total cholesterol	188577	EUR	-0.034 (0.05)	4.9E-01	-0.035 (0.05)	4.8E-01
Triglyceride	188577	EUR	0.1 (0.047)	**3.4E-02**	0.16 (0.04)	**1.0E-04**
Type 2 diabetes	69033	EUR	0.16 (0.076)	**3.5E-02**	0.09 (0.067)	1.8E-01
Fasting Glucose	58074	EUR	0.03 (0.078)	7.0E-01	0.01 (0.061)	8.7E-01
Fasting Insulin	51750	EUR	0.01 (0.088)	9.1E-01	-0.03 (0.062)	6.3E-01
IBD	34652	EUR	0.12 (0.054)	**2.5E-02**	0.08 (0.051)	1.2E-01
IBD Crohn's disease	20883	EUR	0.12 (0.06)	**4.7E-02**	0.1 (0.055)	6.9E-02
IBD Ulcerative colitis	27432	EUR	0.07 (0.058)	2.3E-01	0.03 (0.064)	6.4E-01
**Category (III)**						
AMD*	59462	EUR+ASN	0.01 (0.019)	6.0E-01	0.03 (0.016)	6.7E-02
AMD Geographic Atrophy	48518	EUR+ASN	0.03 (0.023)	2.0E-01	0.05 (0.022)	**2.1E-02**
AMD Neovascular	54938	EUR+ASN	-0.01 (0.036)	7.8E-01	0.0006 (0.015)	9.7E-01
Alzheimer's disease	54162	EUR	0.12 (0.073)	1.0E-01	0.06 (0.062)	3.3E-01
ADHD	5415	ALL	-0.09 (0.13)	4.9E-01	-0.13 (0.123)	2.9E-01
Bipolar disorder	16731	ALL	-0.12 (0.069)	8.0E-02	-0.002 (0.063)	9.8E-01
Major depression disorder	18759	EUR	-0.06 (0.096)	5.3E-01	-0.08 (0.086)	3.5E-01
Schizophrenia	83550	ALL	-0.03 (0.042)	4.8E-01	-0.04 (0.034)	2.4E-01
**Category (IV)**						
Educational attainment	328917	EUR	-0.01 (0.05)	8.4E-01	-0.13 (0.04)	**1.0E-03**
**Category (V)**						
Age at menarche	370000	EUR	-0.02 (0.06)	7.7E-01	-0.03 (0.064)	7.0E-01
Age at menopause	69360	EUR	-0.12 (0.062)	5.4E-02	-0.17 (0.055)	**2.0E-03**
Longevity > 90	23850	EUR	0.21 (0.229)	3.6E-01	0.3 (0.193)	1.2E-01
Leukocyte telomere length	37684	EUR	0.18 (0.108)	9.7E-02	-0.16 (0.108)	1.4E-01

*The majority of individuals are with European decent (> 94%). P values < 0.05 are marked in bold. AMD=age-related macular degeneration. ADHD=attention deficit hyperactivity disorder. IBD=inflammatory bowel disease.

Supplementary Table 13: MR-Egger regression for causal determinates of IEAA

The table presents the results from Mendelian randomization Egger (MR-Egger) regression analysis[12] to test the causal effects of complex traits on IEAA. The first two columns list the test trait and number of GWAS SNPs ($P < 5.0E-08$) as instrumental variables. The macro column of "Causal" lists the bias-reduced estimate of causal effect (exposure -> IEAA) that already removes the bias due to pleiotropic effect. The macro column of "Pleiotropy" lists the estimate of average pleiotropic effect from SNPs directly affecting IEAA. A significant P value indicates directional pleiotropy that pleiotropic effects over the SNPs are not balanced about the null.

The MR-Egger analysis was conducted on a trait with the number of independent leading variants greater than 2 as well as the effect size (SE) statistics was available in the GWAS summary data. As a result, the analysis was not conducted for attention deficit hyperactivity disorder, major depression and two subtypes of age-related macular degeneration. The traits are ordered by category (I) GWAS of anthropometric traits conducted by GIANT consortium, (II) GWAS of lipid, metabolic, and inflammatory outcomes and diseases, (III) GWAS of neurodegenerative and neuropsychiatric disorders, (IV) cognitive functioning and educational attainment traits, and (V) longevity, reproductive ageing and mitotic clock related traits.

Due to our low sample size and the fact that we had only few genome wide significant SNPs associated with IEAA, our MR analysis could only evaluate the following causal model: instrumental variables/SNPs -> test trait -> epigenetic age acceleration. Since two IEAA-associated loci (6p22.3 and 6p22.2) co-locate with CpGs that contribute to the Horvath estimate of DNAm age, we carried out a sensitivity analysis that evaluated whether any of the instrumental variables (i.e. published GWAS hits) located in the IEAA-associated region 6p22.3 (genome coordinates 18,101,894 to 18,190,957 bp) or 6p22.2 (genome coordinates 25,585,844 to 25,671,873 bp). None of the test SNPs turned out to be located in these two regions, i.e. our MR analysis was not confounded by possible direct effects of SNPs acting on IEAA. Further, we repeated the MR analysis after extending the two regions to a buffer of 1 Mb. At most 2 SNPs were removed from the following test traits: waist circumference, height, low density lipoprotein total

cholesterol, and education. The level of significance for causal or pleiotropic p values remained largely the same in the sensitivity check. For example, the causal effect of LDL on IEAA had an effect size (SE) of 0.435 (0.191) and a p value of 0.023 based on all instrumental variables. The effect size (SE) became 0.437 (0.191) and p value became 0.022 after we dropped two SNPs in the buffer region from the analysis.

Trait	#SNPs	Causal		Pleiotropy	
		β_E (SE)	P	β_{0E} (SE)	P
**Category (I)**					
Waist circumference	58	1.992 (1.196)	9.6E-02	-0.05 (0.034)	1.4E-01
Waist to hip ratio	37	-0.308 (2.06)	8.8E-01	0.012 (0.053)	8.3E-01
BMI	95	1.403 (0.734)	5.6E-02	-0.03 (0.018)	8.9E-02
Height	188	0.004 (0.002)	1.3E-01	-0.026 (0.018)	1.6E-01
**Category (II)**					
High density lipoprotein	244	0.375 (0.241)	1.2E-01	-0.029 (0.011)	**6.7E-03**
Low density lipoprotein	202	0.435 (0.191)	**2.3E-02**	-0.023 (0.011)	**3.1E-02**
Total cholesterol	255	0.473 (0.234)	**4.4E-02**	-0.014 (0.011)	1.8E-01
Triglyceride	167	0.613 (0.283)	**3.0E-02**	-0.018 (0.012)	1.4E-01
Type 2 diabetes	50	0.075 (0.262)	7.7E-01	-0.015 (0.026)	5.6E-01
Fasting Glucose	28	-0.017 (0.024)	4.7E-01	-0.012 (0.019)	5.1E-01
Fasting Insulin	15	0.093 (0.083)	2.7E-01	0.008 (0.024)	7.5E-01
IBD	114	0.125 (0.18)	4.9E-01	-0.006 (0.027)	8.3E-01
IBD Crohn's disease	98	0.101 (0.119)	4.0E-01	-0.006 (0.025)	8.2E-01
IBD Ulcerative colitis	78	0.163 (0.189)	3.9E-01	-0.024 (0.036)	5.1E-01
**Category (III)**					
AMD	15	-0.053 (0.092)	5.6E-01	0.023 (0.036)	5.2E-01
AMD Geographic Atrophy		--	--	--	--
AMD Neovascular					
Alzheimer's disease	19	-0.384 (0.547)	4.8E-01	0.017 (0.061)	7.8E-01
ADHD	0	--	--	--	--
Bipolar disorder	4	-0.998 (1.258)	4.3E-01	0.102 (0.211)	6.3E-01
Major depression disorder	0	--	--	--	--
Schizophrenia	95	-0.991 (0.567)	8.1E-02	0.068 (0.044)	1.3E-01
**Category (IV)**					
Educational attainment	114	2.348 (2.043)	2.5E-01	-0.039 (0.037)	2.9E-01
**Category (V)**					
Age at menarche	367	-1.026 (0.358)	**4.1E-03**	0.034 (0.012)	**5.5E-03**
Age at menopause	54	-0.433 (0.148)	**3.5E-03**	0.051 (0.027)	5.5E-02
Longevity > 90	1	--	--	--	--
Leukocyte telomere length	8	1.773 (4.65)	7.0E-01	0.01 (0.32)	9.8E-01

P values < 0.05 are marked in bold. AMD=age-related macular degeneration. ADHD=attention deficit hyperactivity disorder. IBD=inflammatory bowel disease. -- denotes not available.

Supplementary Table 14: MR-Egger regression analysis for causative determinants of EEAA

The table presents the results from Mendelian randomization Egger (MR-Egger) regression analysis[12] to test the causal effects of complex traits on EEAA. The first two columns list the test trait and number of GWAS SNPs (P < 5.0E-08) as instrumental variables. The macro column of "Causal" lists the bias-reduced estimate of causal effect (exposure -> EEAA) that already removes the bias due to pleiotropic effect. The macro column of "Pleiotropy" lists the estimate of average pleiotropic effect from SNPs directly affecting EEAA. A significant P value indicates directional pleiotropy that pleiotropic effects over the SNPs are not balanced about the null.

The MR-Egger analysis was conducted on a trait with the number of independent leading variants greater than 2 as well as the effect size (SE) statistics was available in the GWAS summary data. As a result, the analysis was not conducted for attention deficit hyperactivity disorder, major depression, and two subtypes of age-related macular degeneration.

The traits are ordered by category (I) GWAS of anthropometric traits conducted by GIANT consortium, (II) GWAS of lipid, metabolic, and inflammatory outcomes and diseases, (III) GWAS of neurodegenerative and neuropsychiatric disorders, (IV) cognitive functioning and educational attainment traits, and (V) longevity, reproductive ageing and mitotic clock related traits.

Trait	#SNPs	Causal		Pleiotropy	
		β_E (SE)	P	β_{0E} (SE)	P
**Category (I)**					
Waist circumference	58	-1.477 (1.487)	3.2E-01	0.058 (0.042)	1.7E-01
Waist to hip ratio	37	-3.361 (3.151)	2.9E-01	0.084 (0.081)	3.0E-01
BMI	95	0.095 (0.967)	9.2E-01	0.015 (0.025)	5.6E-01
Height	188	0.004 (0.003)	1.6E-01	-0.03 (0.024)	2.0E-01
**Category (II)**					
High density lipoprotein	244	0.112 (0.305)	7.1E-01	-0.011 (0.013)	4.1E-01
Low density lipoprotein	202	0.509 (0.237)	**3.2E-02**	-0.022 (0.013)	1.1E-01
Total cholesterol	255	0.719 (0.327)	**2.8E-02**	-0.025 (0.015)	8.8E-02
Triglyceride	167	0.639 (0.371)	8.5E-02	-0.025 (0.016)	1.1E-01
Type 2 diabetes	50	0.067 (0.341)	8.4E-01	-0.029 (0.033)	3.8E-01
Fasting Glucose	28	-0.005 (0.027)	8.6E-01	-0.013 (0.021)	5.4E-01
Fasting Insulin	15	0.09 (0.109)	4.1E-01	0.043 (0.028)	1.2E-01
IBD	114	-0.345 (0.238)	1.5E-01	0.08 (0.036)	**2.5E-02**
IBD Crohn's disease	98	-0.29 (0.163)	7.6E-02	0.087 (0.035)	**1.2E-02**
IBD Ulcerative colitis	77	-0.026 (0.26)	9.2E-01	0.024 (0.049)	6.3E-01
**Category (III)**					
AMD	15	-0.035 (0.091)	7.0E-01	-0.001 (0.034)	9.8E-01
AMD Geographic Atrophy		--	--	--	--
AMD Neovascular					
Alzheimer's disease	19	-1.204 (0.715)	9.2E-02	0.127 (0.065)	5.2E-02
ADHD	0	--	--	--	--
Bipolar disorder	4	-0.273 (1.14)	8.1E-01	-0.019 (0.098)	8.4E-01
Major depression disorder	0	--	--	--	--
Schizophrenia	95	-1.09 (0.663)	1.0E-01	0.085 (0.047)	7.1E-02
**Category (IV)**					
Educational attainment	114	-4.629 (2.626)	7.8E-02	0.078 (0.047)	1.0E-01
**Category (V)**					
Age at menarche	367	-0.171 (0.476)	7.2E-01	0.013 (0.016)	4.2E-01
Age at menopause	54	-0.186 (0.192)	3.3E-01	0.022 (0.034)	5.2E-01
Longevity > 90	1	--	--	--	--
Leukocyte telomere length	8	4.246 (4.008)	2.9E-01	-0.248 (0.282)	3.8E-01

P values < 0.05 are marked in bold. AMD=age-related macular degeneration, ADHD=attention deficit hyperactivity disorder. -- denotes not available.

Supplementary Table 15: Sensitivity analysis for significant MR Egger regression

For those traits showing significant causal effects on IEAA (or EEAA), we performed sensitivity analysis to check if the significance dominated from the instrumental variables (IV) that co-located with CpG sites from the DNAm age predictors. We conducted stratified analysis that we classified the IV according to their positions from the DNAm age predictors. The table below lists three results for each test trait, according to the set of IV SNPs: (1) All includes all the IV SNPs, (2) set I excludes the IV SNPs that co-locate with the CpG sites (+/- 1Mb), and (3) set II only includes the IV SNPs that co-locate with the CpG sites. Overall, the causal P values remain the same level of significance using the IV SNPs from set I. The causal effect of age at menarche on IEAA and the causal effect of triglyceride on IEAA exhibited slightly less significant P values after removing the IV SNPs co-locating the CpG sites from the DNAm age predictors.

			Causal		Pleiotropy	
**Trait**	**IV**	**#SNPs**	**β_E (SE)**	**P**	**β_{0E} (SE)**	**P**
IEAA						
Age at menarche	All	367	-1.026 (0.358)	**4.1E-03**	0.034 (0.012)	5.5E-03
	set I	292	-1.014 (0.399)	**1.1E-02**	0.038 (0.014)	6.5E-03
	set II	75	-1.152 (0.829)	1.6E-01	0.023 (0.027)	3.9E-01
Age at menopause	All	54	-0.433 (0.148)	**3.5E-03**	0.051 (0.027)	5.5E-02
	set I	34	-0.499 (0.166)	**2.7E-03**	0.069 (0.029)	1.6E-02
	set II	20	-0.184 (0.315)	5.6E-01	-0.006 (0.059)	9.2E-01
Low density lipoprotein	All	377	0.343 (0.173)	**4.7E-02**	-0.016 (0.008)	3.2E-02
	set I	288	0.439 (0.188)	**2.0E-02**	-0.016 (0.008)	6.2E-02
	set II	89	-0.141 (0.429)	7.4E-01	-0.012 (0.018)	4.9E-01
Triglyceride	All	314	0.544 (0.242)	**2.5E-02**	-0.013 (0.008)	1.3E-01
	set I	220	0.551 (0.282)	5.1E-02	-0.018 (0.01)	6.1E-02
	set II	94	0.553 (0.499)	2.7E-01	<0.001 (0.017)	1.0E+00
EEAA						
Low density lipoprotein	All	377	0.429 (0.217)	**4.8E-02**	-0.018 (0.01)	6.5E-02
	set I	366	0.438 (0.212)	**3.9E-02**	-0.016 (0.009)	9.5E-02
	set II	11	-8.046 (5.088)	1.1E-01	0.103 (0.148)	4.9E-01
Total cholesterol	All	441	0.664 (0.289)	**2.2E-02**	-0.022 (0.011)	4.4E-02
	set I	418	0.676 (0.281)	**1.6E-02**	-0.019 (0.011)	7.1E-02
	set II	23	-4.533 (4.32)	2.9E-01	0.052 (0.124)	6.7E-01

Causal P values < 0.05 marked in bold.

Supplementary Table 16: MR-Egger regression for causal effect of IEAA on fathers age at death

We performed an Ad-hoc MR-Egger regression to test the causal effect of IEAA on fathers age at death. We found a nominal evidence for the causal relationship (β_{causal} (SE) =0.04 (0.02) with P_{causal}=1.9x10^{-2} , β_{causal}(SE) = -0.02 (0.01) with $P_{pleiotropy}$= 2.2x10^{-2}). In the Egger regression, we used a total of 8 leading variants associated with IEAA as instrumental variables, selected by LD-based clumping procedure in PLINK with a threshold of r^2 set at 0.1 in a window size of 250kb. The table below summarizes the GWAS results associated with the 8 SNPs. In addition, we did not test the other direction of causality, fathers age at death -> IEAA, as there was no any SNPs associated with fathers age at death at genome-wide significance[13].

				IEAA			Fathers		
**Chr**	**SNP**	**MAF**	**Effect allele**	**Beta***	**SE**	**P**	**Beta***	**SE**	**P**
3	rs11706810	0.45	T	-0.41	0.07	1.63E-09	0.011	0.005	1.80E-02
5	rs7734992	0.44	T	-0.59	0.10	1.78E-09	0.002	0.005	7.10E-01
5	rs2736099	0.36	A	0.63	0.09	1.29E-12	0.012	0.005	1.60E-02
6	rs56267547	0.10	G	0.90	0.11	3.30E-15	0.008	0.008	3.30E-01
6	rs11758925	0.39	G	-0.53	0.07	1.16E-13	-0.006	0.005	1.90E-01
6	rs138907444	0.12	C	0.64	0.10	9.74E-10	0.000	0.006	9.90E-01
6	rs73397619	0.29	T	0.46	0.07	2.30E-10	-0.007	0.005	1.80E-01
17	rs78781855	0.22	T	0.48	0.08	5.59E-09	-0.001	0.006	8.10E-01

*in units of year

Supplementary Notes

Supplementary Note 1: Description of datasets

We used 8 cohorts comprising 15 studies for association analysis with IEAA and EEAA, respectively. We estimated DNAm ages and epigenetic age acceleration (IEAA/EEAA) variables using our software, which is freely available online (https://dnamage.genetics.ucla.edu). Below, we briefly describe general information of each cohort/study and the process for DNA methylation quantification. Genomic platforms and quality controls for SNPs prior imputation, imputed genotypes and GWAS analysis are summarized in **Supplementary Tables 1&3**.

Study 1: Framingham Heart Study Cohort (FHS)

The Framingham Heart Study (FHS)[14] is a large-scale longitudinal study started in 1948, initially investigating the common factors of characteristics that contribute to cardiovascular disease (CVD), https://www.framinghamheartstudy.org/index.php. The study at first enrolled participants living in the town of Framingham, Massachusetts, who were free of overt symptoms of CVD, heart attack or stroke at enrollment. In 1971, the study started FHS Offspring Cohort to enroll a second generation of the original participants' adult children and their spouses (n= 5124) for conducting similar examinations[15]. Participants from the FHS Offspring Cohort were eligible for our GWAS study if they attended the eighth examination cycle (2005-2008) and consented to having their DNA to be used for genetic research. There are a total of 2,471 participants available for both DNA methylation and SNP array data, used in the current study. All participants provided written informed consent at the time of each examination visit. The study protocol was approved by the Institutional Review Board at Boston University Medical Center (Boston, MA). The FHS data are available in dbGaP (accession number: phs000342.v17.p10 and phs000724.v2.p9). ***DNA methylation quantification*** Peripheral blood samples were collected at the 8th examination. Genomic DNA was extracted from buffy coat using the Gentra Puregene DNA extraction kit (Qiagen) and bisulfite converted using EZ DNA Methylation kit (Zymo Research Corporation). DNA methylation quantification was

conducted in two laboratory batches using the Illumina Infinium HumanMethylation450 array (Illumina). Methylation beta values were generated using the Bioconductor *minfi* package with background correction. Sample exclusion criteria included poor SNP matching of control positions, missing rate >1%, outliers from principal components of the genetic relationship matrix, and sex mismatch. In addition, we excluded individuals with leukemia or received chemotherapy. Additional sample exclusions included those with mismatches in their reported sex and methylation-predicted sex as well as methylation predicted tissues that were not blood. Lastly, samples whose correlation with our reference population was r<0.80 were excluded.

Study 2: TwinsUK

TwinsUK is the biggest UK registry of volunteer twins, http://www.twinsuk.ac.uk/, started in 1992 to study the genetic and environmental aetiology associated with age-related diseases and health aging[16]. The study comprises ~ 12,000 monozygotic and dizygotic twins across the UK with ages between sixteen and ninety eight, predominantly female twins. SNP array data are available for ~6000 individuals. Prior imputation, population stratification assessment was based on principle component analysis (PCA) to exclude genetic outlier individuals. Individuals in our study were requested free of sever diseases (e.g. cancer) and available for both SNP array and DNA methylation, leaving 492 female individuals (no twin pairs) remained in analysis. ***DNA methylation quantification*** DNA samples were extracted from whole blood using DNeasy kit (Qiagen, Inc) and bisulfite converted using EZ DNA methylation kit (Zymo Research Corporation). DNA methylation levels were detected using the Illumina Infinium HumanMethylation450 array (Illumina) and the methylation betas were generated using the R package *minfi* with background correction. Raw beta levels were first applied the beta mixture quantile dilation (*BMIQ*) method to correct for the technical issues. Probe exclusion criteria including probes mapped to multiple locations to the reference sequence, and probes where more than 1% of subjects had detection p-value > 0.05. Individuals with over 5% missing probes, with mismatched sex, and with mismatched genotypes were excluded.

Studies 3-5, 12-15: Women's Health Initiative (WHI)

The Women's Health Initiative (WHI) study is a national study that began in 1993 which enrolled postmenopausal women between the ages of 50-79 years into either one of two three randomized clinical trials (RCTs), http://www.nhlbi.nih.gov/whi/. We included 2,902 WHI participants available for both SNP and DNA methylation array data including (1) 949 women from the study EMPC/AS315 and (2) 1,953 women from the study BA23. The study EMPC aims to investigate the epigenetic mechanism underlying cardiovascular disease mediated by ambient particular matter (PM), https://www.whi.org/researchers/data/WHIStudies/StudySites/AS315/Pages/home.aspx/. The other study BA23 focuses on identifying miRNA and genomic biomarkers of congenital heart disease (CHD), integrating the biomarkers into diagnostic and prognostic predictors of CHD and other related phenotypes, and other objectives can be found in https://www.whi.org/researchers/data/WHIStudies/StudySites/BA23/Pages/home.aspx.

The EMPC and BA23 study populations are across three sub WHI cohorts including (a) GARNET, (b) WHIMS and (c) SHARe. All the three sub cohorts involve large-scale sample sizes (N > 4,800) but only ~ 10% women are available for both DNA methylation and SNP array data. The WHI data are available in dbGAP (study accession: phs000746.v1.p3). ***DNA methylation quantification for EMPC*** Illumina Infinium HumanMethylation450 BeadChip data from the Northwestern University Genomics Core Facility for WHI-EMPC participants sampled in stages 1a, 1b, and 2 were quality controlled and batch adjusted. Batch adjustment involved applying empirical Bayes methods of adjusting for stage and plate as implemented in ComBat [17]. ***DNA methylation quantification for BA23*** In brief, bisulfite conversion using the Zymo EZ DNA Methylation Kit (Zymo Research, Orange, CA, USA) as well as subsequent hybridization of the HumanMethylation450k Bead Chip (Illumina, San Diego, CA), and scanning (iScan, Illumina) were performed according to the manufacturers protocols by applying standard settings. DNA methylation levels (β values) were determined by calculating the ratio of intensities between methylated (signal A) and un-methylated (signal B) sites. Specifically, the β value was calculated from the intensity of the methylated (M corresponding to signal A) and un-methylated (U corresponding to signal B) sites, as the ratio of fluorescent signals β

= Max(M,0)/[Max(M,0)+Max(U,0)+100]. Thus, β values range from 0 (completely un-methylated) to 1 (completely methylated).

GARNET (study 3, 4)

The GARNET (Genomics and Randomized Trials Network) study involves case-control design including 4 case groups: type 2 diabetes, myocardial infarction, stroke and venous thrombosis. The study aims to identify genetic variants associated with response of treatments related to clinical conditions or public health significance, more details listed in https://www.genome.gov/27541119/genomics-and-randomized-trials-network-garnet/. There are 589 women available for both DNA methylation and SNP array data. Most of the participants are Caucasians. We removed 18 women identified as genetic outliers identified from PCA in conjunction with multidimensional scaling (MDS) analysis, leaving 571 individuals remained in analysis.

WHIMS (study 5)

The WHIMS (Women's Health Initiative Memory Study) is a large, randomized, double-blind, placebo-controlled clinical trial cohort that investigate the effect of postmenopausal hormone therapy on dementia and mild cognitive functioning[18]. The participants were 65 and 79 years of age at baseline and free of probable dementia. There are 397 non- Hispanic Whites women available for DNA methylation and SNP array data, with genetic ancestry confirmed by PCA-MDS analysis.

SHARe (studies 12-15)

The SHARe (SNP Health Association Resource) study comprises participants enrolled in WHI whose self-reported ethnicity was African American or Hispanic. We separated the participants into two studies according to their ethnicity: study 10 for African American and study 11 for Hispanic participants (as listed in **Table 1** in main article). Of the 665 self-reported African Americans, there are 7 women identified as mismatched genetic ancestry from PCA-MDS analysis and one identified as outlier in terms of IEAA type age acceleration (IEAA > 40 years) removed from analysis, leaving 657 women remained in GWAS in study. Of the 415 self-reported Hispanic participants, there are 87 women identified as mismatched genetic ancestry from PCA-MDS analysis, leaving 328 remained in analysis.

Study 6: EPIC-Norfolk

European Prospective Investigation into Cancer (EPIC) -Norfolk [19] is a prospective population-based cohort study as part of the European-wide programme EPIC; http://www.srl.cam.ac.uk/epic/. EPIC-Norfolk recruited 25,000 men and women aged 40-79 years at baseline between 1993 and 1997 from 35 participating general practices in Norfolk. Individuals provided information about lifestyle behavioural factors including diet and activity and attended for a baseline health check including the provision of blood samples for concurrent and future analysis. EPIC-Norfolk is currently in its fifth health check phase. In this study 1,117 individuals that have both SNP array and DNA methylation data were used. ***DNA methylation quantification*** DNA methylation was measured in DNA samples in whole blood samples collected at baseline. Bisulfite conversion of DNA was performed using the EZ DNA methylation kit (Zymo Research, Orange, CA, USA). Converted DNA was assayed by PCR (Polymerase Chain Reaction) and gel electrophoresis. DNA methylation levels were then quantified using the Illumina Infinium HumanMethylation450 array according to manufacturer's instructions. To ensure that each plate performed well, each 96 well DNA sample plate contained two duplicate samples and the correlation between the duplicates was checked to meet Illumina's recommendations. The average correlation between the duplicate samples is 98%. Background correction of the Illumina HM450 k data was performed using the R package *minfi* [20] and methylation beta values were then generated. The sample exclusion criteria was based on analyses performed on the autosomal and X and Y chromosomes separately. The proportion of missing data in each sample (sample call rate) was calculated and samples with a call rate ≤ 0.99 were excluded. For duplicate samples, the sample with the least CpG detection percentage was excluded. Furthermore, to ensure that the methylation intensity distributions of the samples follow the same pattern, the distribution of the autosome, X and Y chromosome methylation markers was plotted separately for female and male individuals. One additional sample was excluded based on its X chromosome distribution, and another additional sample was excluded based on its autosomal distribution since their distribution did not follow the expected pattern.

Study 7: Baltimore Longitudinal Study of Aging (BLSA)

The Baltimore Longitudinal Study of Aging (BLSA) is American's longest-running scientific study of aging established in 1958[21], https://www.blsa.nih.gov/. The study involves longitudinal design for investigating physical and cognitive changes associated with normal aging, free of disease. The BLSA has a rigours screening process for potential participants. All the participants are in good health at time of enrolment and aged 20 years and older.

In BLSA, blood samples were collected for DNA extraction, and genome-wide genotyping was completed for 1231 subjects using Illumina 550K. The analysis was restricted to subjects with European ancestry and each analysis was further adjusted for the top two principal components derived from an EIGENSTRAT analysis[22], leaving genotyping completed for 848 participants of European ancestry. The BLSA data are available in dbGAP (study accession: phs000215.v2.p1). Information about SNP array data including imputed makers can be found in [23]. Of the 848 BLSA individuals, 444 participants are available for DNA methylation data remained in analysis. ***DNA methylation quantification*** DNA was quantified using Quant-iT Picogreen Reagent (Invitrogen, Grand Island, NY, USA) according to the manufacturer's instructions. 1 ug of DNA was bisulfite treated using the EZ-96 DNA methylation kit (Zymo Research, Irvine, CA, USA) according to the manufacturer's specifications for the 450k array. Converted genomic DNA was eluted in 22 µl of elution buffer. DNA methylation level was measured using Illumina Infinium HD Methylation Assay (Illumina) according to the manufacturer's instructions. Background subtraction was applied using the *preprocessIllumina* command in the *minfi* Bioconductor package[20]

There are a total 501 participants available for both DNA methylation and SNP array data remained in analysis.

Study 8: Invecchiare in Chianti, aging in the Chianti area (InChianti)

The InChianti (Invecchiare in Chianti, aging in the Chianti area) cohort is a representative population-based study of older persons enrolling individuals aged 20 years and older from two areas in the Chianti region of Tuscany, Italy, http://inchiantistudy.net/wp/ . One major goal of the study is to translate epidemiological research into geriatric clinical tools, ultimately advancing clinical applications in older persons. Genotyping were performed on ~1200

participants. Individuals less than 97% of genotyped completeness (n=12), heterozygosity rate of less than 30% (n=5) and misspecified sex based on heterozygosity of the X chromosome SNPs (n=1) were excluded, as listed in [24]. European ancestry of 1210 Chianti individuals was confirmed by the first two principal components derived from an EIGENSTRAT analysis[22]. The InChianti data are available in dbGAP (study accession: phs000215.v2.p1). Of the 1210 individuals, 444 participants are available for DNA methylation data remained in analysis. All participants provided written informed consent to participate in this study. The study complied with the Declaration of Helsinki. The Italian National Institute of Research and Care on Aging Institutional Review Board approved the study protocol.

DNA methylation quantification Genomic DNA was extracted from buffy coat samples using an AutoGen Flex and quantified on a Nanodrop1000 spectrophotometer prior to bisulfite conversion. Genomic DNA was bisulfite converted using Zymo EZ-96 DNA Methylation Kit (Zymo Research Corp., Irvine, CA) as per the manufacturer's protocol. CpG methylation status of 485,577 CpG sites was determined using the Illumina Infinium HumanMethylation450 BeadChip (Illumina Inc., San Diego, CA) as per the manufacturer's protocol and as previously described[25]. Initial data analysis was performed using GenomeStudio 2011.1 (Model M Version 1.9.0, Illumina Inc.). Threshold call rate for inclusion of samples was 95%. Quality control of sample handling included comparison of clinically reported sex versus sex of the same samples determined by analysis of methylation levels of CpG sites on the X chromosome[25]. Background subtraction was applied using the *preprocessIllumina* command in the *minfi* Bioconductor package[20].

Study 9: Brisbane Systems Genetics Study (BSGS)

The Brisbane Systems Genetics Study (BSGS) [26,27] is a family-based study for elucidating genetic factors mediating endophenotypes and complex diseases. BSGS comprises genotype and phenotype data from 314 families (n=962 individuals)[26], collected at Queensland Institute of Medical Research (QIMR, http://www.qimrberghofer.edu.au/qtwin/). The BSGS study was approved by the Queensland Institute for Medical Research Human Research Ethics Committee. All participants gave informed written consent.

We obtained SNP array and DNA methylation data for 614 individuals from 177 families recruited as part of the BSGS[26,27]. Families consist of adolescent monozygotic or dizygotic twins, their siblings and parents. Full details of individual exclusion criteria (including verification of European ancestry) and the quality control of SNP genotypes are given in Medland et al[28]. ***DNA methylation quantification*** As described in[29], DNA was extracted from peripheral blood lymphocytes from samples that were time matched to sample collection of PAXgene tubes for gene expression studies in BSGS. Bisulphite converted DNA samples were hybridized to the 12 sample Illumina HumanMethylation450 BeadChips using the Infinium HD Methylation protocol and Tecan robotics (Illumina, San Diego, CA, USA). Samples were randomly placed with respect to the chip they were measured on and to the position on that chip in order to avoid any confounding with family. Box-plots of the red and green intensity levels and their ratio were used to ensure that no chip position was under- or over-exposed, with any outlying samples repeated. Similarly, the proportion of probes with detection P value less than 0.01 was examined to confirm strong binding of the sample to the array. Raw intensity values were background corrected using the Genome Studio software performing normalization to internal controls and background subtraction.

Studies 10 & 11: Lothian Birth Cohorts (LBC) of 1921 and 1936

The Lothian Birth Cohorts (LBC)[30] of 1921 and 1936 are surveyed based longitudinal studies to examine distribution and causes of cognitive functioning changes across most of the human life course, http://www.lothianbirthcohort.ed.ac.uk/. The participants in LBC1921 (born in 1921) were enrolled for those undertook Scottish Mental Survey in 1932 while the participants in LBC1936 (born in 1936) were enrolled for those undertook the survey in 1947. Both surveys were associated with general intelligence tests for children at age 11 years and were carried out by the Scottish Council for Reach in Education. The LBC1921 (n=550) begun in 1999 had been examined 4 waves at mean ages 79, 83, 97 and 90 years while the LBC 1936 (n=1091) begun in 2004 had been examined 2 waves at mean ages 70 and 73 years[30].

We obtained DNA methylation data used in the earlier study for predicting all-cause mortality[29] in which SNP array data were also available for the study individuals. The LBC1921 comprising 446 wave 1 individuals (n_{deaths}=292) referred as study 8 and the LBC1936 comprising 920 wave 1 individuals (n_{deaths}=106) referred as study 9 in our analysis. All participants were of White Scottish ancestry. Following informed consent, venesected whole blood was collected for DNA extraction in both LBC1921 and LBC1936. Ethics permission for the LBC1921 was obtained from the Lothian Research Ethics Committee (Wave 1: LREC/1998/4/183). Ethics permission for the LBC1936 was obtained from the Multi-Centre Research Ethics Committee for Scotland (Wave 1: MREC/01/0/56), the Lothian Research Ethics Committee (Wave 1: LREC/2003/2/29). Written informed consent was obtained from all individuals. LBC methylation data have been submitted to the European Genome-phenome Archive under accession number EGAS00001000910. ***DNA methylation quantification*** As described in[29], DNA was extracted from 514 whole blood samples in LBC1921 and from 1,004 samples in LBC1936. Raw intensity data were backgroundcorrected and methylation beta-values generated using the R minfi package[20]. Quality control analysis was performed to remove probes with a low (<95%) detection rate at P <0.01. Manual inspection of the array control probe signals was used to identify and remove low quality samples. The Illumina-recommended threshold was used to eliminate samples with a low call rate (samples with <450,000 probes detected at P <0.01). As SNP genotyping was previously performed on LBC samples, genotypes derived from the 65 SNP control probes on the methylation array using the wateRmelon package[31] were compared to those obtained from the genotyping array to ensure sample integrity. Samples with a low match of genotypes with SNP control probes, which could indicate sample contamination or mix-up, were excluded (n = 9). Moreover, eight subjects whose predicted sex, based on XY probes, did not match reported sex were also excluded.

Supplementary Note 2: Co-localization of clock CpGs in 6p22.3 and 6p22.2

The leading variants in 6p22.3 and 6p22.2 are close to two of the 353 epigenetic markers (CpGs) that are used in the Horvath estimate of DNAm age: cg22736354 in *NHLRC1* (~8.5 kb) and cg06493994 in *SCGN* (~27.8 kb, **Table 2**), respectively. To test whether the observed SNP associations in these two loci might be technical artifacts resulting from confounding between CpG probes and SNPs, we carried out a sensitivity analysis of all the genome-wide significant SNPs in 6p22.3 and in 6p22.2, in which the association models were adjusted for cg22736354 and cg06493994, respectively.

Sensitivity analysis for genetic loci that co-locate with CpGs

Two CpGs from the Horvath age estimate co-located with IEAA related genetic loci. Specifically, CpG cg22736354 is located near (-21kb ~68kb) 104 genome-wide significant SNPs in 6p22.3. Similarly, CpG cg06493994 is located near (-67kb ~ 19kb) 108 genome-wide significant SNPs in 6p22.2 (**Table 2 and Supplementary Table 4**). We performed a sensitivity analysis of our GWAS study of IEAA, which adjusted the regression model for covariate cg22736354 (cg06493994) in 6p22.3 (6p22.2). The sensitivity analysis involved WHI cohorts (stratified by study, i.e. studies 3, 4, 12 and 14). Next, the results from each WHI cohort study were combined using a fixed effect models weighted by inverse variance

After the adjustment, the median GWAS meta-analysis p value in the WHI studies declined from 3.3×10^{-3} to 0.02 for the SNPs in 6p22.3 and from 1.6×10^{-3} to 0.03 for the SNPs in 6p22.2 (**Supplementary Figs. 9 & 10**). Thus, we cannot rule out that the parts of the association signals in 6p22.3 (near *NHLRC1*) and 6p22.2 (near *SCGN*) arise from their close proximity to two CpGs used in the DNAm age estimate.

Supplementary Note 3: Detailed summary of Transcriptomic studies in blood

Our large-scale transcriptomic studies identified a total of 11 significant cis-eQTLs with IEAA or EEAA. Integrating our GWAS and eQTL summary data, summary data-based Mendelian randomization (SMR) analysis identified 6 out of 11 cis-eQTLs exhibiting pleiotropic associations with our epigenetic age acceleration measures. Here we provide a detailed summary stratified by locus.

IEAA locus at 3q25.33

The IEAA locus at 3q25.33 spans 300 kb across six genes comprising 23 SNPs with genome-wide significant associations (**Supplementary Table 4**). The leading SNP rs11706810 exhibits a highly significant association with the expression levels of *KPNA4* (cis-eQTL $P=4.2x10^{-23}$ in the FHS study, see **Table 4**). Similarly, expression levels of *KPNA4* exhibit a significant pleiotropic association with IEAA (SMR: $P=5.1x10^{-5}$, **Table 4** and **Supplementary Table 13**). Overall, our results suggest that higher expression of *KPNA4*, which encodes a protein subunit that imports karyophilic proteins to the nuclear pore complex, is associated with increasing IEAA.

TPMT expression linked to IEAA at 6p22.3

IEAA exhibits genome-wide significant associations with 104 SNPs in 6p22.3 which are located near two genes *NHLRC1* (NHL repeat containing E3 ubiquitin protein ligase 1) and *TPMT* (thiopurine S-methyltransferase) including the missense variant rs10949383 in *NHLRC1* and the missense variants (rs1142345) in *TPMT* (**Supplementary Table 4**). Our cis-eQTL analysis show that 6p22.3 harbors a gene *TPMT* whose expression levels are robustly associated with SNPs in 6p22.3 (Stouffer's meta-analysis $P=2.0x10^{-62}$ combined FHS, GTEx and LSMeta studies listed in **Supplementary Table 11**). IEAA-increasing alleles are also positively associated with the expression levels of *TPMT*. Similarly, our SMR analysis revealed a highly significant pleiotropic association between IEAA and *TPMT* expression levels (Stouffer's meta-analysis $P=2.8x10^{-18}$combined SMR FHS, GTEx, and LSMeta studies listed in **Table 4**). Compared to *TPMT*, the second gene *NHLRC1* in the 6p22.3 locus exhibits a much weaker cis-eQTL effect (unadjusted $P=2.2x10^{-5}$ in the FHS, FDR $q=2.9x10^{-4}$ in NTR twin study, FDR $q=3.6x10^{-5}$ in the NESDA study,

Supplementary Table 11). Overall, our transcriptomic studies suggest that there is a pleiotropic association between IEAA and *TPMT* expression levels or that *TPMT* has a direct causal effect on IEAA.

IEAA locus at 6p22.2

IEAA exhibits genome-wide significant associations with 108 SNPs in 6p22.2 near several genes including *LRRC116A*, *SCGN*, *HIST1H2AC*, and *BTN3A2*. An eQTL analysis in the FHS revealed that more than 100 GWAS SNPs are associated with the expression levels of *HIST1H2AC* and *BTN3A2* but only one SNP is associated with *LPRC16A* (**Supplementary Table 10**). However, our SMR analysis failed to detect significant pleiotropic associations between IEAA and gene expression levels.

STXBP4 expression linked to IEAA at 17q22

IEAA exhibits genome-wide significant associations with 18 SNPs in the 17q22 locus near *STXBP4* (syntaxin binding protein 4). The lead SNP rs78781855 and other neighboring SNPs are highly robust cis-eQTLs for *STXBP4* (in FHS: $P=1.0x10^{-88}$, **Supplementary Tables 10 & 11)**. Similarly, our SMR analysis detects a significant pleiotropic association between IEAA and *STXBP4* ($P = 6.2x10^{-4}$, **Table 4**). Overall, these results demonstrate that higher expression of *STXBP4* is associated with higher IEAA.

4p16.3 associated with EEAA

EEAA exhibits genome-wide significant associations with 58 SNPs in the 4p16.3 locus (**Table 2, Supplementary Figs. 5a & 7a** and **Supplementary Table 4**). While the leading SNP rs10937913 is located in gene *TNIP2*, the remaining genome-wide significant SNPs are located near *FAM193A* and *RFN4* (**Supplementary Fig. 5a**).

A surrogate of the leading SNP, rss2341303, has a highly significant association with *RFN4* (cis-eQTL P=$1.6x10^{-10}$, **Table 4**) which can also be observed for several other GWAS SNPs (**Supplementary Table 10)**. Similarly, we find a significant pleiotropic association between *RFN4* and EEAA (SMR $P= 2.0x10^{-3}$, **Table 4**). Overall, these results suggest that high expression values of *RNF4* are associated with increased EEAA.

<u>10p11.21-10p11.1 associated with EEAA</u>

EEAA exhibits genome-wide associations with 381 SNPs in the two consecutive loci 10p11.21-10p11.1 which span across 1Mb and harbors many zinc finger genes (*ZNF248*, *ZNF33BP1*,*ZNF25*, *ZNF33A*, *ZNF37A*, **Supplementary Table 4** and **Supplementary Figs. 5 b&c)** and contains a relatively high proportion of INDEL variants (12%, 46 out of 381 SNPs).

Our cis-eQTL analysis shows that the GWAS SNPs in 10p11.21-10p11.1 are significantly associated with the expression levels of the pseudo-gene *HSD17B7P2* ($P=2.3x10^{-21}$ in the GTEx study) and gene *ZNF25* ($P=3.0x10^{-8}$ in the FHS study) and ZNF248 ($P=3.6x10^{-6}$ in the LSMeta study, **Supplementary Table 10)**. The SMR analysis suggests that EEAA could have a positive pleiotropic association with both HSD17B7P2 ($P=6.9x10^{-6}$) and ZNF25 ($P=2.8x10^{-3}$, **Table 4**).

Single causal variants in most cis-eQTLs

The cis-acting SNPs related to the expression levels of *KPNA4, STXBP4, RNF4* and *HSD17B7P2* exhibit non-significant HEIDI test P values, which suggests that a single causal variant confers the pleiotropic association between the respective gene expression levels and epigenetic age acceleration at the respective loci (**Methods** and **Supplementary Table 11**). However, this is not the case for *ZNF25* in 10p11.1 and *TPMT* in 6p22.3.

Rather, 6p22.3 harbors multiple causal variants (**Table 2**) that could give rise to the pleiotropic association between *TPMT* and IEAA.

Supplementary Note 4: Age-related or complex trait studies used in analysis of shared genetic relationships with IEAA/EEAA

Below, we briefly describe large-scale GWAS studies that were cross referenced to our GWAS study of epigenetic age acceleration. The GWAS results are corresponding to previously published articles, except cognitive functioning traits using the Health and Retirement Study (HRS) data. We performed two GWAS for two cognitive functioning traits our previous study[32]. We performed overlap and genetic correlation analysis to relate our epigenetic age acceleration traits to other complex traits. We only used stage 1 data for analysis except for the GWAS summary data for education attainment, which was based on a meta-analysis of discovery and cohorts. We did not perform the genetic correlation analysis for some test traits if the entire population ancestry was of non-European ancestry or heritability was not detectable ($h^2 < 0$ yielded from the LD regression analysis).

Age at Huntington's Disease (HD) motor onset (PMID:26232222)

The GWAS meta-analysis was performed for 4,082 HD subjects collected from the Massachusetts HD Center Without Wall (MAHDC) and the Genetics Modifies of HD (GeM-HD) [33]. All the HD subjects are with European ancestry. Age at onsite motor was adjusted for the influence of *HTT* CAG repeat then the residual values were used as phenotypes for quantitative association analysis. We downloaded the summary data from the Genetic Modifies of Motor Onset Age (GeM-MOA, http://chgr.partners.org/cgi-bin/gem.moa/gem.moa.py), with access granted by the GeM-MOA consortium.

Age at menarche (PMID:28436984)

A large-scale GWAS meta-analysis was performed on ~370,000 women of European descent, comprising 40 studies from ReproGen consortium, in addition to 23andMe and UK Biobank studies[34]. We used the summary results of the analysis of ReproGen and UK Biobank participants (N=252,514) markers for the analysis.

Age at menopause (PMID: 26414677)

The study performed a meta-analysis of GWAS for age at natural menopausing using a total of 33 studies comprising 69,360 individuals[35]. All individuals are of European decent. The summary results of ~ 2.6 million markers can be downloaded from http://www.reprogen.org/research.html.

Age-related macular degeneration (AMD: 23455636)

A large-scale GWAS meta-analysis was performed in the study including >17,100 advanced AMD cases and >60,000 controls of European and Asian ancestry in the analysis[36], conducted by AMD Gene Consortium Study of Age Related Macular Degeneration. We downloaded three summary results of ~ 2.4 million markers at discovery stage for studying advanced AMD versus control subjects, including two AMD subtypes: geographic atrophy and neovascular, and AMD with any of subtype, from http://www.sph.umich.edu/csg/abecasis/public/amdgene2012/.

Alzheimer's disease (PMID: 24162737)

The IGAP consortium performed a GWAS meta-analysis on 74,046 individuals of European ancestry[37]. We downloaded the summary results of GWAS from http://www.pasteur-lille.fr/en/recherche/u744/igap/igap_download.php. Two sets of association results are available. The first set includes the GWAS results of meta-analysis based upon 17,008 Alzheimer's disease cases and 37,154 controls at stage 1 analysis. A total of 11,632 SNPs exhibited moderate evidence of association ($P < 1.0 \times 10^{-3}$) at stage 1. The second set includes the P values of the 11,632 SNPs from the final meta-analysis that combined stages 1 &2 results.

DIAGRAM Type 2 diabetes (PMID: 22885922)

The DIAbetes Genetics Replication and Meta-analysis (DIAGRAM) consortium performed a GWAS meta-analysis for type 2 diabetes (T2D) in [38]. The first set includes the GWAS results of meta-analysis based upon 22,669 cases and 58,119 controls of European decent at stage 1 analysis. The stage 2 analysis was performed on 1,178 cases and 2,472 controls of Pakistani decent. A subsequent meta-analysis was performed to combine both stages 1 and 2 results.

Stage 1 and the combined meta-analyses results are available for download from http://diagram-consortium.org/downloads.html.

Education attainment (PMID: 27225129)

The study presents the meta-analysis of GWAS for educational attainments using 293,723 individuals at discovery phase and 111,349 at replication phase[39]. All the study individuals were collected restricted to with European ancestry, at cohort level. Education years of all individuals were assessed at or above age 30. GWAS summary data are provided for the results of a meta-analysis of all discovery and replication cohorts (N=328,917) except 23andMe due to IRB restrictions. The summary data involves three GWAS results stratified by gender (males, females and both). We downloaded the summary results for GWAS from https://www.thessgac.org/data.

GIANT body fat distribution (PMID: 25673412)

The genetic investigation of anthropometric trait (GIANT) consortium performed a GWAS meta-analysis for body fat distribution traits on 22,459 individuals of European (the majority), East Asian, South Asia, and African American ancestry[40]. We used the GWAS results using the individuals of European for waist circumference and waist-to-hip ratio. Each involved three GWAS results stratified by gender (males, females and both). We downloaded the summary results of GWAS from https://www.broadinstitute.org/collaboration/giant/index.php/GIANT_consortium_data_files.

GIANT BMI (PMID: 25673413)

The GIANT consortium performed a GWAS meta-analysis for BMI on 339,224 individuals including 322,154 (95%) with European descent and 17,072 with non-European decent[41]. The GWAS results can be downloaded from,https://www.broadinstitute.org/collaboration/giant/index.php/GIANT_consortium_data_files.

GIANT Height (PMID: 20881960)

The GIANT consortium performed a GWAS meta-analysis for height on 183,727 individuals of Europe ancestry[42]. More than 99.8% of the individuals were adults. Genome-wide meta-analysis were conducted using 133,653 individuals for stage 1 analysis, yielding 207 loci reaching Meta P < 5.0E-06. In stage 2 analysis, at least one SNP from each of the 207 loci were selected for additional GWAS using the other 50,074 individuals. GWAS results can be downloaded from, https://www.broadinstitute.org/collaboration/giant/index.php/GIANT_consortium_data_files

IIBDGC Inflammatory bowel disease (PMID: 26192919)

The International Inflammatory Bowel Disease Genetics Consortium (IIBDGC) performed a large-scale GWAS meta-analysis for studying inflammatory bowel disease (IBD) on 86,640 European individuals at discovery phase and 9,846 individuals of East Asian, Indian or Iranian decent at replication phase[43]. Three traits were analyzed including two forms of IBD: ulcerative colitis and Crohn's disease, as well as IBD in either form. As the results for replication analysis performed by Bayesian trans-ancestry meta-analysis that reports Bays factor rather than *P* values, we only conducted the overlap analysis using the GWAS results at discovery phase for the three IBD forms, respectively. At discovery phase, 34652 individuals were available for genome-wide genotype data and the other 51988 individuals were only available for Immnuochip genotype data. The GWAS results using the genotypes of 34,652 individuals can be downloaded from http://www.ibdgenetics.org/.

The Health and Retirement Study (HRS, PMID: 26830004)

The Health and Retirement Study (1992-2012) is a longitudinal panel study of a representative sample of Americans over age 50 (and their spouses), collected every two years. We previously performed GWAS of two cognitive functioning traits on ~ 12,450 individuals[32]. The first one, cognitive aging slope, is a longitudinal measure of age-related cognitive decline and the second one, dementia, is an overall binary dementia status that combined the assessments for dementia at wave 8 (diagnosed in year 2006) and wave 9 (diagnosed in year 2008), respectively. We

either restricted the GWAS analysis to a specific ethnic group (European, African American, and Hispanic ancestry) or used all individuals.

Lipoproteins study (PMID: 24097068)

We downloaded the GWAS meta-analysis results[44] from http://csg.sph.umich.edu//abecasis/public/lipids2013/. The GWAS was performed 188,577 individuals of European ancestry including 94,595 from 23 studies genotyped with GWAS arrays and 93,982 individuals from 37 studies genotyped with Metabochip array.

Longevity study (PMID: 24688116)

The GWAS meta-analyses study was performed in 98,066 individuals of European ancestry[45], including discovery, replication and joint analyses. The summary results at discovery phase analysis (N=23,850) can be downloaded from http://hmg.oxfordjournals.org/content/23/16/4420/suppl/DC1. The results include two types of association *P* values with respect to (1) individuals with longevity > 85 versus < 65 and (2) individuals with longevity > 90 versus < 65. We only report the results for comparison (2), i.e. longevity >90 versus <65. The results for the first comparison were similar.

MAGIC Glycemic measure (PMID: 22581228)

The Meta-Analyses of Glucose and Insulin-related traits (MAGIC) consortium performed a GWAS meta-analysis on (a) 58,074 non-diabetic individuals for fasting glucose trait and (b) 51,750 non-diabetic individuals for fasting insulin trait, respectively. All study individuals are of European descent. BMI variation was accounted for GWAS in both traits. Data on glycemic traits have been contributed by MAGIC investigators and have been downloaded from www.magicinvestigators.org and the relevant article can be found in [46].

Parkinson's disease study (PMID: 19915575)

A two stage genome wide association study for Parkinson's disease was conducted in 13,625 individuals of European ancestry[47]. For our analysis, we utilized the results of 463187 SNPs at stage 1 phase released in dbGAP with accession: pha002868.1 downloaded from, http://www.ncbi.nlm.nih.gov/projects/gap/cgibin/analysis.cgi?study_id=phs000501.v1.p1&pha=2868.

PGC Attention-deficit/ hyperactivity disorder (PMID: 20732625)

The Psychiatric Genomics Consortium (PGC) performed a GWAS meta-analysis for studying attention-deficit/hyperactivity disorder (ADHD)[48]. The study individuals were across pedigree data (2,064 trios) and case-control (896 cases and 2,455 controls) data sets, with the majority of European ancestry. The GWAS results can be downloaded from http://www.med.unc.edu/pgc/downloads.

PGC bipolar disorder (PMID: 21926972)

The PGC performed a combined GWAS meta-analysis for studying bipolar disorder[49]. Association analysis was performed on a primary analysis (7,481 cases and 9,250 controls), followed by a replication analysis tested on the top 34 SNPs (4,496 independent cases and 42,422 independent controls). Ancestry of individuals identified by multi-dimensional scaling (MDS) analysis was used to identify ancestry of study individuals and was used to correct population stratification in association analysis. The GWAS results of the primary stage can be downloaded from http://www.med.unc.edu/pgc/downloads.

PGC major depression disorder (PMID: 22472876)

The PGC performed a combined GWAS meta-analysis for studying major depression disorder[50]. Association analysis was performed on discovery phased (9,240 cases and 9,519 controls of European ancestry), replication phase (6,783 cases and 50,695 controls), and mega-analysis (9,238 major depression disorder cases/8,039 controls, and 6,998

bipolar disorder cases and 7775 controls) for cross-disorder trait, the last two only involved a small number of SNPs ($m \leq 819$). Only the GWAS results for discovery phase are available for download from http://www.med.unc.edu/pgc/downloads.

PGC Schizophrenia (PMID: 25056061)

The PGC performed a multi-stage large-scale GWAS meta-analysis for studying schizophrenia up to 36,989 cases and 113,075 controls[51]. The majority of individuals are of European ancestry. A primary GWAS meta-analysis was performed on 49 case-control samples (46 of European and 3 of East Asian ancestry, 34,241 cases and 45,604 controls) and 3 family-based samples of European ancestry (1,235 parent affected-offspring trios). The overlap analysis was based on the primary results downloaded from http://www.med.unc.edu/pgc/downloads.

Supplementary Reference

1. Roadmap Epigenomics, C. *et al.* Integrative analysis of 111 reference human epigenomes. *Nature* **518**, 317-30 (2015).
2. Zhu, Z. *et al.* Integration of summary data from GWAS and eQTL studies predicts complex trait gene targets. *Nat Genet* **48**, 481-7 (2016).
3. Bojesen, S.E. *et al.* Multiple independent variants at the TERT locus are associated with telomere length and risks of breast and ovarian cancer. *Nat Genet* **45**, 371-84, 384e1-2 (2013).
4. Horvath, S. DNA methylation age of human tissues and cell types. *Genome Biol* **14**, R115 (2013).
5. Hannum, G. *et al.* Genome-wide methylation profiles reveal quantitative views of human aging rates. *Mol Cell* **49**, 359-67 (2013).
6. Almasy, L. & Blangero, J. Multipoint quantitative-trait linkage analysis in general pedigrees. *Am J Hum Genet* **62**, 1198-211 (1998).
7. Bulik-Sullivan, B.K. *et al.* LD Score regression distinguishes confounding from polygenicity in genome-wide association studies. *Nat Genet* **47**, 291-5 (2015).
8. Yang, J., Lee, S.H., Goddard, M.E. & Visscher, P.M. GCTA: a tool for genome-wide complex trait analysis. *Am J Hum Genet* **88**, 76-82 (2011).
9. Lee, S.H., Wray, N.R., Goddard, M.E. & Visscher, P.M. Estimating missing heritability for disease from genome-wide association studies. *Am J Hum Genet* **88**, 294-305 (2011).
10. Visscher, P.M. *et al.* Statistical power to detect genetic (co)variance of complex traits using SNP data in unrelated samples. *PLoS Genet* **10**, e1004269 (2014).
11. Codd, V. *et al.* Identification of seven loci affecting mean telomere length and their association with disease. *Nat Genet* **45**, 422-7, 427e1-2 (2013).
12. Bowden, J., Davey Smith, G. & Burgess, S. Mendelian randomization with invalid instruments: effect estimation and bias detection through Egger regression. *Int J Epidemiol* **44**, 512-25 (2015).
13. Pilling, L.C. *et al.* Human longevity is influenced by many genetic variants: evidence from 75,000 UK Biobank participants. *Aging (Albany NY)* **8**, 547-60 (2016).
14. Dawber, T.R., Meadors, G.F. & Moore, F.E., Jr. Epidemiological approaches to heart disease: the Framingham Study. *Am J Public Health Nations Health* **41**, 279-81 (1951).
15. Kannel, W.B., Feinleib, M., McNamara, P.M., Garrison, R.J. & Castelli, W.P. An investigation of coronary heart disease in families. The Framingham offspring study. *Am J Epidemiol* **110**, 281-90 (1979).
16. Moayyeri, A., Hammond, C.J., Hart, D.J. & Spector, T.D. The UK Adult Twin Registry (TwinsUK Resource). *Twin Res Hum Genet* **16**, 144-9 (2013).
17. Johnson, W.E., Rabinovic, A. & Li, C. Adjusting batch effects in microarray expression data using Empirical Bayes methods. *Biostatistics* **8**, 118-127 (2007).
18. Shumaker, S.A. *et al.* Conjugated equine estrogens and incidence of probable dementia and mild cognitive impairment in postmenopausal women: Women's Health Initiative Memory Study. *JAMA* **291**, 2947-58 (2004).
19. Day, N. *et al.* EPIC-Norfolk: study design and characteristics of the cohort. European Prospective Investigation of Cancer. *Br J Cancer* **80 Suppl 1**, 95-103 (1999).
20. Aryee, M.J. *et al.* Minfi: a flexible and comprehensive Bioconductor package for the analysis of Infinium DNA methylation microarrays. *Bioinformatics* **30**, 1363-9 (2014).
21. Ferrucci, L. The Baltimore Longitudinal Study of Aging (BLSA): a 50-year-long journey and plans for the future. *J Gerontol A Biol Sci Med Sci* **63**, 1416-9 (2008).

22. Price, A.L. *et al.* Principal components analysis corrects for stratification in genome-wide association studies. *Nat Genet* **38**, 904-9 (2006).
23. Tanaka, T. *et al.* Genome-wide association study of vitamin B6, vitamin B12, folate, and homocysteine blood concentrations. *Am J Hum Genet* **84**, 477-82 (2009).
24. Melzer, D. *et al.* A genome-wide association study identifies protein quantitative trait loci (pQTLs). *PLoS Genet* **4**, e1000072 (2008).
25. Moore, A.Z. *et al.* Change in Epigenome-Wide DNA Methylation Over 9 Years and Subsequent Mortality: Results From the InCHIANTI Study. *J Gerontol A Biol Sci Med Sci* (2015).
26. Powell, J.E. *et al.* The Brisbane Systems Genetics Study: genetical genomics meets complex trait genetics. *PLoS One* **7**, e35430 (2012).
27. McRae, A.F. *et al.* Contribution of genetic variation to transgenerational inheritance of DNA methylation. *Genome Biol* **15**, R73 (2014).
28. Medland, S.E. *et al.* Common variants in the trichohyalin gene are associated with straight hair in Europeans. *Am J Hum Genet* **85**, 750-5 (2009).
29. Marioni, R.E. *et al.* DNA methylation age of blood predicts all-cause mortality in later life. *Genome Biol* **16**, 25 (2015).
30. Deary, I.J., Gow, A.J., Pattie, A. & Starr, J.M. Cohort profile: the Lothian Birth Cohorts of 1921 and 1936. *Int J Epidemiol* **41**, 1576-84 (2012).
31. Pidsley, R. *et al.* A data-driven approach to preprocessing Illumina 450K methylation array data. *BMC Genomics* **14**, 293 (2013).
32. Lu, A.T. *et al.* Genetic architecture of epigenetic and neuronal ageing rates in human brain regions. *Nat Commun* **8**, 15353 (2017).
33. Genetic Modifiers of Huntington's Disease, C. Identification of Genetic Factors that Modify Clinical Onset of Huntington's Disease. *Cell* **162**, 516-26 (2015).
34. Day, F.R. *et al.* Genomic analyses identify hundreds of variants associated with age at menarche and support a role for puberty timing in cancer risk. *Nat Genet* (2017).
35. Day, F.R. *et al.* Large-scale genomic analyses link reproductive aging to hypothalamic signaling, breast cancer susceptibility and BRCA1-mediated DNA repair. *Nat Genet* **47**, 1294-303 (2015).
36. Fritsche, L.G. *et al.* Seven new loci associated with age-related macular degeneration. *Nat Genet* **45**, 433-9, 439e1-2 (2013).
37. Lambert, J.C. *et al.* Meta-analysis of 74,046 individuals identifies 11 new susceptibility loci for Alzheimer's disease. *Nat Genet* **45**, 1452-8 (2013).
38. Morris, A.P. *et al.* Large-scale association analysis provides insights into the genetic architecture and pathophysiology of type 2 diabetes. *Nat Genet* **44**, 981-90 (2012).
39. Okbay, A. *et al.* Genome-wide association study identifies 74 loci associated with educational attainment. *Nature* **533**, 539-42 (2016).
40. Shungin, D. *et al.* New genetic loci link adipose and insulin biology to body fat distribution. *Nature* **518**, 187-96 (2015).
41. Locke, A.E. *et al.* Genetic studies of body mass index yield new insights for obesity biology. *Nature* **518**, 197-206 (2015).
42. Lango Allen, H. *et al.* Hundreds of variants clustered in genomic loci and biological pathways affect human height. *Nature* **467**, 832-8 (2010).
43. Liu, J.Z. *et al.* Association analyses identify 38 susceptibility loci for inflammatory bowel disease and highlight shared genetic risk across populations. *Nat Genet* **47**, 979-86 (2015).
44. Global Lipids Genetics, C. *et al.* Discovery and refinement of loci associated with lipid levels. *Nat Genet* **45**, 1274-83 (2013).

45. Deelen, J. *et al.* Genome-wide association meta-analysis of human longevity identifies a novel locus conferring survival beyond 90 years of age. *Hum Mol Genet* **23**, 4420-32 (2014).
46. Manning, A.K. *et al.* A genome-wide approach accounting for body mass index identifies genetic variants influencing fasting glycemic traits and insulin resistance. *Nat Genet* **44**, 659-69 (2012).
47. Simon-Sanchez, J. *et al.* Genome-wide association study reveals genetic risk underlying Parkinson's disease. *Nat Genet* **41**, 1308-12 (2009).
48. Neale, B.M. *et al.* Meta-analysis of genome-wide association studies of attention-deficit/hyperactivity disorder. *J Am Acad Child Adolesc Psychiatry* **49**, 884-97 (2010).
49. Psychiatric, G.C.B.D.W.G. Large-scale genome-wide association analysis of bipolar disorder identifies a new susceptibility locus near ODZ4. *Nat Genet* **43**, 977-83 (2011).
50. Major Depressive Disorder Working Group of the Psychiatric, G.C. *et al.* A mega-analysis of genome-wide association studies for major depressive disorder. *Mol Psychiatry* **18**, 497-511 (2013).
51. Schizophrenia Working Group of the Psychiatric Genomics, C. Biological insights from 108 schizophrenia-associated genetic loci. *Nature* **511**, 421-7 (2014).
