# Supplementary material for: GWAS of epigenetic aging rates in blood reveals a critical role for TERT
Source: Nat Commun. 2018 Jan 26;9:387. doi: 10.1038/s41467-017-02697-5 (PMC5786029; doi:10.1038/s41467-017-02697-5)
Supplement: Supplementary file 2 — Description of Additional Supplementary Files [file 41467_2017_2697_MOESM2_ESM.pdf]

Description of Additional Supplementary Files

File Name: Supplementary Data 1

Description: The Excel file lists the summary of 264 SNP or INDEL markers associated with IEAA and 440 variants associated with EEAA at a genomewide significance level. For each SNP, we report summary data from our fixed effects meta-analysis, the Bayesian meta-analysis and various annotations HaploReg 4.1, http://www.broadinstitute.org/mammals/haploreg/haploreg.php. The annotations include conserved regions by GERP (PMID:21152010) and SiPhy(PMID: 19478016) scores, DNase tracks, involved proteins and motifs, GWAS hits listed in NHGRI/EBI and functional annotation listed in dbSNP database. The first sheet lists descriptions of each column. The second and third sheet report the variants associated with IEAA and EEAA, respectively.

File Name: Supplementary Data 2

Description: Summary of the top variants associated with IEAA/EEAA Our meta GWAS analysis identified 5 loci for IEAA and 3 loci for EEAA, which can be represented by 9 leading variants. For each leading variant, the Excel table reports the meta-analysis statistics with respect to both IEAA and EEAA.

File Name: Supplementary Data 3

Description: A large-scale cis-eQTL analysis in blood identifies 11 candidate functional genes for epigenetic age acceleration Our cis-eQTL analysis identified 11 candidate functional genes underlying the SNP association with IEAA or EEAA. The Excel file reports the SNP with the most significant cis-eQTL P values for each significant cis gene. The eQTL results were obtained from the following databases: (1) Framingham Heart Study (N=5,257), (2) GTEx (n=338), (3) LSMeta (N=5,311), (4) NTR (2,494 twins), and (5) NESDA (N=1,895). The column "No.Signif." (number significant) reports the total number of SNP-gene expression pairs whose local false discovery rate satisfied (FDR q < 0.05 for FHS, GTEx and LSMeta, FDR q < 0.01 for NTR and NESDA). At each cis-gene, the SNP index with the most significant ciseffect is presented stratified by database, followed by nominal eQTL P values (or FDR q values), Meta GWAS P values (associated with epigenetic age acceleration) and LD [illegible] with respect to the leading SNP of each genomewide significant locus.

File Name: Supplementary Data 4

Description: Blood cell counts versus variants at the TERT locus The table summarizes SNP association results between variants in the TERT locus and imputed blood cell count estimates (based on DNA methylation levels). Estimated cell types include naïve CD4+ T, naïve CD8+ T, exhausted CD8+ T cells (defined as CD28-negative CD45R-negative), plasmablasts, CD4+ T, nature killer cells, monocytes, and granulocytes. The estimation of the blood cell counts is described in (Horvath and Levine 2015, PMCID: PMC4621253). The association analysis for IEAA and EEAA involved the same n=5,373 individuals that were part of our study individuals for GWAS.
.Supplementary Data 5: LDSC genetic correlation between epigenetic age acceleration and 233 complex traits Relating GWAS results for IEAA/EEAA to those of 233 complex traits using the LD Hub software (Zheng et al 2016, PMCID: PMC5542030). The Excel file reports genetic correlation coefficients for all summary-level GWAS results currently available in the LD Hub central database.

File Name: Supplementary Data 5
Description: LDSC genetic correlation between epigenetic age acceleration and 233 complex traits We related our GWAS results for IEAA/EEAA to those of 233 complex traits using the LD Hub software (Zheng et al 2016, PMCID: PMC5542030). The Excel file reports genetic correlation coefficients for all summary-level GWAS results currently available in the LD Hub central database.

File Name: Supplementary Data 6
Description: GWAS-based overlap analysis between epigenetic age acceleration and a broad category of other complex traits The excel file summarizes the results from GWAS-based overlap analysis with a broad category of large scale GWAS results for age-related outcomes or complex diseases using our GWAS results for 3 IEAA/EEAA. To define lists of genes that relate to a given trait (disease status or epigenetic age acceleration) we used a MAGENTA gene score cut-off at the top k percentile of the autosomal genes. Next, we assessed which of these genes is also located in the top k percentile list of blood epigenetic aging. For a few studies, we were not able to conduct the genetic correlation analysis due to small sample size (N < 5000), negative heritability estimates. The first sheet provides explanations. The second (for IEAA) and third sheet (for EEAA) report various statistics from the overlap analysis including Hypergeometric P-value, false discovery rate, and the proportion of overlapping genes using gene lists based on a cut off value of 2.5% or 10%, respectively. The P and FDR values are marked in bold if < 0.05. Abbreviations for genetic ancestry are listed in the following: Asians (ASN), Africans (AFR), Americans (AMR), and Europeans (EUR). Genes that relate both to epigenetic age acceleration in blood and an age related disease, according to the "overlap analysis" of GWA studies, are listed in sheets 4:5 with respect to IEAA and sheets 6:7 with respect to EEAA.
